# Sex-Specific Glucose Homeostasis and Anthropometric Responses to Sleeve Gastrectomy in Obese Patients

**DOI:** 10.3390/nu11102408

**Published:** 2019-10-09

**Authors:** Mark A. Taylor, Lukasz Szczerbinski, Anna Citko, Magdalena Niemira, Maria Gorska, Hady Razak Hady, Adam Kretowski

**Affiliations:** 1 School of Medicine, University of California, San Francisco 505 Parnassus Ave, San Francisco, CA 94143, USA; Mark.Taylor@ucsf.edu; 2 Department of Endocrinology, Diabetology and Internal Medicine, Medical University of Bialystok, Sklodowskiej-Curie 24A, 15-276 Bialystok, Poland; mgorska25@wp.pl (M.G.); adamkretowski@wp.pl (A.K.); 3 Clinical Research Centre, Medical University of Bialystok, Sklodowskiej-Curie 24A, 15-276 Bialystok, Poland; anna.citko@umb.edu.pl (A.C.); magdalena.niemira@umb.edu.pl (M.N.); 4 1st Clinical Department of General and Endocrine Surgery, Medical University of Bialystok, Sklodowskiej-Curie 24A, 15-276 Bialystok, Poland; hadyrazakh@wp.pl

**Keywords:** bariatric surgery, diabetes, gender, glucose homeostasis, sex, sleeve gastrectomy

## Abstract

Bariatric surgery rapidly and effectively treats obesity and its comorbidities like dysregulated glucose homeostasis. Despite the sex-balanced incidence of obesity in most human populations, women have sought this intervention more frequently than men. However, as the number of bariatric surgeries rapidly rises, it is increasingly urgent to understand how sex-specific differences may emerge in metabolic and anthropometric parameters. Hundred fifty-four obese patients (47% men and 53% women) from the Bialystok Bariatric Surgery Study underwent sleeve gastrectomy and were measured for 25 parameters at baseline (immediately prior to surgery) and at four follow-up visits over one year. We used generalized linear mixed models to detect sex-specific differences in the time series of responses parameters. Unlike most previous studies with older cross-sections of men than women, our cohort was age-matched, and men were less healthy at baseline. Of parameters that showed a significant cohort-wide (across-sex) response, 14 (56%) also showed sex-specific responses with men improving more than women. In particular, men remitted in diabetes symptoms more strongly, rapidly, and durably than women. Taken together, our results indicate that men may benefit more from sleeve gastrectomy and that this difference in improvement may be related to more progressed morbidity prior to surgery independent of age.

## 1. Introduction

Obesity poses a major challenge for modern medicine, exacerbated by the rapid global spread of Western-pattern lifestyles characterized by overnutrition and low physical activity [1]. Among potential treatments for obesity, bariatric surgery is the single most effective, leading to rapid and durable amelioration of both obesity and its comorbidities such as type 2 diabetes (T2D) [2,3]. Over the past decade, sleeve gastrectomy has become one of the most popular and effective bariatric surgical techniques, and its central mechanism of action relies upon calorie restriction [4,5]. Although a growing body of evidence has demonstrated bariatric surgery’s broad efficacy across patient populations, it has also introduced uncertainty with respect to sex-specific effects [6]. Historically, women have sought this intervention more frequently than men with 80% of bariatric surgeries performed on female patients, and this imbalance has persisted for the past decade [7,8,9]. However, obesity incidence is nearly equal in both sexes [10], and as the number of bariatric surgeries continues to accelerate [11], it is increasingly urgent to understand whether and how differential responses may occur. Previous studies have yielded conflicting results: Some find significant differences in particular parameters while others either fail to replicate these results or find opposite response profiles [2,6,12,13,14]. Most such studies examine restricted sets of metabolic or anthropometric parameters, often, for example, measuring only the concentration of glycated hemoglobin (HbA1c) as a metric of glycemic health. However, potential sex-specific responses to bariatric surgery likely emerge from complex interactions among hormone signaling axes, anatomical, and physiological differences between the sexes. This implies that many traits must be simultaneously measured to understand systemic, sex-specific responses. Here, we performed deep, longitudinal phenotyping for one year on a balanced cohort of men and women to understand how they differed in response to bariatric surgery.

Of previous studies that have detected significant differences in bariatric outcomes between sexes, the majority have found that males respond more favorably than females. This difference occurs in diverse parameters, such as heart rate variability [15], serum leptin levels [16], fat-free mass loss [17], and absolute and relative weight loss [15,16,18,19,20,21,22]. However, these studies are relatively small (n ≈ 10^2^), and fewer but much larger studies (n ≈ 10^4^) have uncovered conflicting evidence showing that women improve more in cardiovascular and metabolic responses [13,14]. The majority of these studies consist of retrospective or prospective cohort observations, and thus likely suffer from confounding between the sexes [23]. Indeed, several confounding variables have been widely detected and replicated in independent patient cohorts such as the greater age, alcohol consumption, smoking history, and severity of pre-existing conditions in men seeking bariatric surgery [8,24]. The greater age of men is likely an extremely important mediating variable in explaining men’s superior responses since many obesity-related morbidities covary with age [25,26]. Thus, there is an outstanding need to examine sex-specific responses to bariatric surgery in an age-matched cohort.

Two primary ways to account for potential mediating variables and to control for confounders have been implemented: (1) Increased sample size, assuming that a random distribution of confounding variables is more likely to be captured in a larger cohort, and (2) adjusting estimates of response effects by explicitly modeling confounders [23,27,28]. The first method is limited by the accuracy of its underlying assumption—in the case of sex, confounding variables may covary as strongly in large samples as in small. The second method is limited by the comprehensiveness of phenotyping so that sufficient variables must be modeled to ensure that the effects of the predictor of interest (in our case, sex) can be isolated from covariates. In evaluating sex-specific responses to bariatric surgery, an important covariate is differences in nutritional habits between men and women. These differences emerge from complex psychosocial processes, emerging in the twin trends that women show greater concern about body image [29] and greater anxiety about bodyweight [30]. This has been specifically confirmed in bariatric surgical candidates with women showing lower psychological well-being and more depressive symptoms related to body image and diet [14]. This, in turn, can lead to more frequent and extreme alterations in dietary habits in women to control weight (for example, more frequent low-carbohydrate diet attempts [31]), which may impact the effect of bariatric surgery [32,33]. Assessing these dietary and nutritional habits is an important aspect of a comprehensive analysis of sex-specific responses to bariatric surgery, and we carefully track and model these habits in this study.

Finally, the mechanistic causes of sex-specific differences in bariatric surgical treatment remain controversial since most investigations of bariatric surgery’s effects have been exclusively conducted in male animal models despite the predominance of female bariatric patients [34]. However, recent sex-inclusive studies have traced causal differences between male and female subjects to the gene-expression level, finding major differences in metabolic regulatory pathways [35,36]. Together, these findings highlight the importance of understanding systemic metabolism in gauging the efficacy of bariatric surgery. We use deep metabolic phenotyping to accomplish this goal, focusing on metrics of dysglycemia. This is especially relevant in bariatric surgical candidates since T2D is a severe and prevalent comorbidity of obesity, occurring in 28% of obese patients [37]. Bariatric surgery generally, and sleeve gastrectomy specifically, provides rapid, highly effective relief for most diabetes features with complete remission of 78% of T2D patients within two years of surgery [2,37]. However, few studies examine multiple metabolic parameters that change upon bariatric surgery, how they differ between men and women, and how they affect dysglycemia status.

We answer four questions in this study: (1) How does sleeve gastrectomy affect metabolic and anthropometric parameters across sexes? (2) What is the most informative set of covariates to isolate predictor effects? (3) Do responses to bariatric surgery depend upon sex, and if so, do men or women respond better? (4) Are there sex-specific trends in dysglycemia resolution?

## 2. Materials and Methods

### 2.1. Study Design

The anthropometric and metabolic data analyzed in this study were generated by the Bialystok Bariatric Surgery Study (BBSS), a longitudinal prospective cohort study of patients who underwent bariatric surgery in the First Clinical Department of General and Endocrine Surgery of the Medical University of Bialystok, Poland [38]. This is the main bariatric surgery center in the province of Podlaskie Voivodeship, performing the most bariatric and metabolic surgeries in northeastern Poland. This center performs several types of bariatric surgery including Roux-en-Y gastric bypass (RYGB), adjustable gastric banding (AGB), and sleeve gastrectomy (SG) in patients referred to the clinic by general practitioners and endocrinologists and who were qualified for the surgery according to the clinical physicians. Only patients who agreed to participate in the research project, specified in detail during written and oral patients consent, were included. In this study, we analyzed patients who underwent sleeve gastrectomy since it represents the vast majority (85%) of all bariatric surgeries performed at the center and in order to eliminate confounding variation in surgery type. The BBSS began enrolling patients in 2016 who underwent a battery of tests one month prior to the intervention and repeated at 1-, 3-, 6-, and 12-month follow-up visits. At each visit, subjects underwent a physical examination, body composition analysis, oral glucose tolerance test (OGTT), and blood testing. Patients also completed self-reported diet and physical activity questionnaires. All subjects gave informed consent to be included in the study prior to their participation, and were informed that all blood tests performed in the Clinical Research Centre of Medical University of Bialystok would be used only for research and would be anonymized. All the patients have access to their results and can withdraw consent at any time. This study was conducted in accordance with the Declaration of Helsinki, and received approval from the Ethics Committee of the Medical University of Bialystok (Project identification code: R-I-002/546/2015).

### 2.2. Study Population

**Figure 1:**
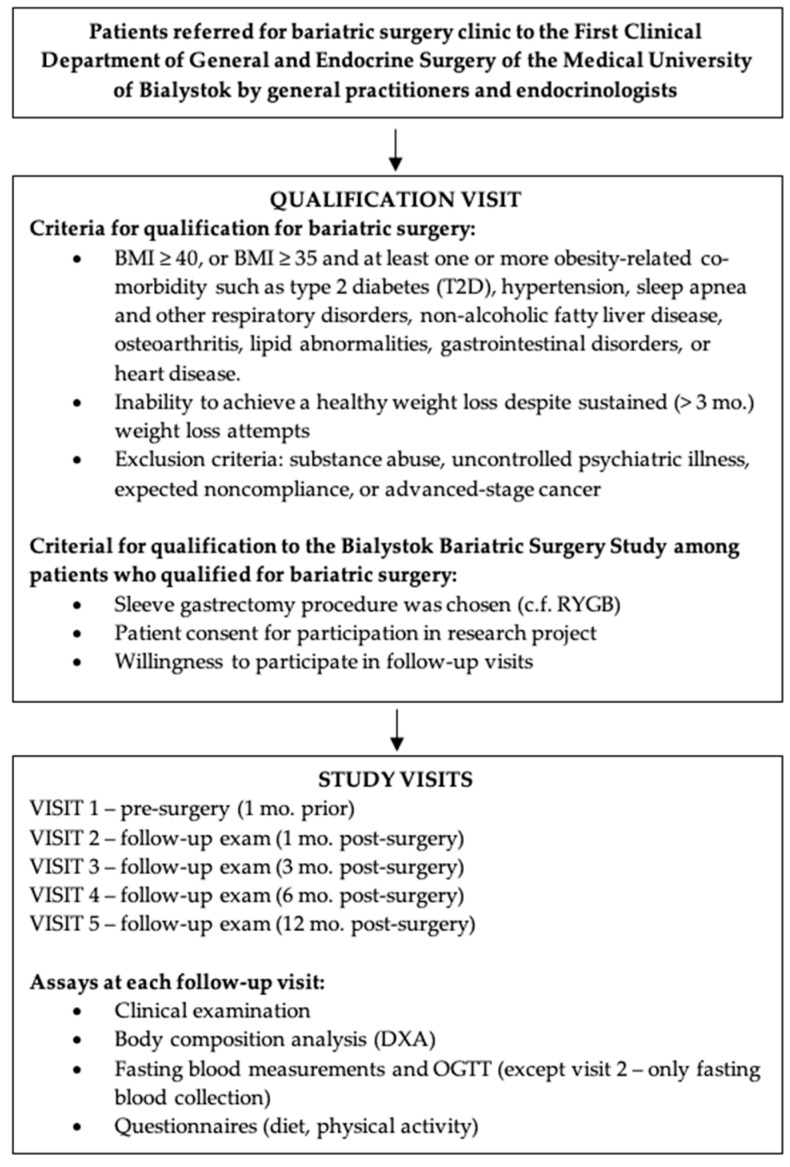
Qualification procedure and criteria for this study.

Among patients undergoing bariatric surgery, 186 patients were enrolled between 2016 and 2019, and 154 (82.8%) who enrolled between 2016 and 2018 were retained for analysis since they had at least three of four follow-up visits including the 12-month follow-up. Inclusion criteria were taken from the National Institutes of Health guidelines for bariatric surgery (BMI ≥ 40 kg/m^2^ or BMI ≥ 35 kg/m^2^ with comorbidities) [39]. Exclusion criteria included any prior bariatric surgery, prior gastrectomy, substance abuse, uncontrolled psychiatric illness, expected lack of compliance, or advanced-stage cancer. Diabetes diagnoses were based on the Diabetes Poland’s criteria (PTD) [40] using glucose concentration measurements during OGTT at 0- and 120-min. Diabetes remission was defined by the American Society for Metabolic and Bariatric Surgery’s guidelines [41]: HbA1c < 6% and fasting blood glucose (FBG) < 100 mg/dL with no antidiabetic medication therapy. Qualification criteria and procedures are summarized in Figure 1.

### 2.3. Assay Protocols and Measurements

Metabolic and anthropometric parameters were measured at baseline and each follow-up exam (individual measurements detailed in Table 1). The OGTT procedure was conducted in accordance with the American Diabetes Association’s (ADA’s) guidelines, beginning between 7:30 and 8:00 a.m. [42]. Prior to the OGTT, patients were instructed to fast for 8–10 h and to avoid physical activity for 24 h, including bicycle or stair use. Patients were further instructed not to take any medications the day of the test, including oral antidiabetic agents for 24 h prior to the visit. OGTTs commenced with baseline blood collection (0 min), followed by oral consumption of the solution of 75 g of glucose dissolved in 300 mL of room-temperature water. Subsequent blood collections were taken at 30, 60, and 120 min following glucose administration. We used these four-point time series to construct OGTT response curves from which we calculated glucose area under the curve (AUC), insulin AUC, Matsuda index, mean insulin concentration, and mean glucose concentration.

Nutritional data were taken from dietary records maintained by patients for three days prior to each visit. In order to construct accurate diet diaries, patients were instructed to record their typical dietary habits for at least three days prior to each visit (including at least one weekend day). These data included the time of consumption, amount of food (mass), and name of the food. These data were standardized into carbohydrate, fat, and protein weighted amounts by trained dieticians using Dieta 4.0 (National Food and Nutrition Institute, Warsaw, Poland), which collated and summarized food consumption. From this, average daily total energy (kcal/day), protein, carbohydrates, and fat intake were calculated. Concomitant with food diaries, physical activity was also assessed using the Polish version of the International Physical Activity Questionnaire-Long Form (IPAQ) [43]. Whole-body dual-energy X-ray absorptiometry (DXA) scans were performed for body composition analysis, using Lunar iDXA (GE Healthcare, Chicago, IL, USA). The total amount of lean body mass (LBM), fat mass (FM) and visceral adipose tissue (VAT) mass were measured and expressed in kilograms. Body composition was also measured using multi-frequency bioelectrical impedance (InBody 220, Biospace, Seoul, Korea) to estimate body fat mass and skeletal muscle mass. Plasma insulin level was measured in duplicate with an immunoradiometric assay (DIAsource ImmunoAssays SA, Nivelles, Belgium). Plasma glucose, serum triglycerides (TG), total cholesterol (CHOL), high-density lipoprotein cholesterol (HDL), low-density lipoprotein cholesterol (LDL), aspartate transaminase (AST), alanine transaminase (ALT) concentrations were measured using the colorimetric methods of Cobas c111 (Roche Diagnostics, Basel, Switzerland). The concentration of glycated hemoglobin A1c (HbA1c) was measured by high-performance liquid chromatography (Bio-Rad VARIANT, Bio-Rad Laboratories, Hercules, CA, USA).

### 2.4. Statistical Analysis

**Baseline cross-sectional analyses**: All analyses were performed in R version 3.6.0 [44]. We tested all continuous response parameters for normality with Shapiro–Wilk’s tests as well as visual inspections of residuals where appropriate. Most response variables failed these tests despite standard transformations, so non-parametric tests were used. Categorical parameters (smoking status, diagnosis, sex, and family history) were presented as frequencies (percentages) and continuous parameters as medians (interquartile range [IQR]). To compare whether medians differed significantly between sexes, we implemented non-parametric median tests from Conover [45]. To test sensitivity in these estimates to possible confounding by age, smoking, diet, physical activity and BMI, we implemented three of five possible quantile regression models to estimate median and IQR adjusted for: (1) Age by model 1, (2) age and smoking status by model 2, (3) age, smoking status, baseline diet (total kcal intake), and baseline physical activity by model 3, (4) age, smoking status, and baseline BMI by my model 4, and (5) age, smoking status, baseline BMI, baseline diet, and baseline physical activity by model 5. Models 1, 2, and 3 were used for continuous variables based upon or highly correlated to BMI (such as weight), while models 1, 4, and 5 were used for all others (Table S1).

**Cohort-wide and cross-sectional time series**: For each response parameter, we fit generalized linear mixed models under gamma distributions. Time was fit as both linear and quadratic fixed effects; age, physical activity (MET-minutes/week), diet (total kcal/day), and sex as fixed effects, and patient ID as a random effect. Model selection was conducted by calculating and comparing restricted estimated maximum likelihoods (REMLs) for each model. Lower REMLs signify more informative models, and we confirmed our selections with likelihood ratio tests between the two models with the lowest scoring REMLs. To determine whether a response parameter was significantly altered during the study across sexes, we examined the significance of time parameters. We then asked whether responses were significantly different between sexes and examined the significance of the time × sex interaction term, fitting sex a fixed instead of a random effect. Finally, we asked whether sex-specific effects may be dependent upon dysglycemia diagnosis and examined the significance of the three-way time × sex × diagnosis interaction term. We compared scaled changes in individual patients of anthropometric, metabolic, and nutritional parameters ([final 12-month follow-up–baseline]/baseline), calculating sex-specific model-adjusted means.

## 3. Results

### 3.1. Cohort-Wide Characteristics

Of 154 analyzed patients at baseline, 16 (10%) were non-diabetic, 58 (38%) showed impaired fasting glucose (IFG) without impaired glucose tolerance (IGT), 26 (17%) showed both, 20 (13%) were newly diagnosed, untreated diabetics, and 27 (18%) were previously diagnosed diabetics undergoing pharmacotherapy. Seven patients (4.5%) presented with prediabetes, a strongly positive family history of diabetes, and were taking prophylactic antidiabetic medication, but they had never received a diagnosis of diabetes and their OGTT tests were negative for T2D. Since definitive diabetes diagnoses through history or OGTT could not be established, these patients’ diagnoses were coded as missing and not included in analyses of diagnosis-specific effects. The median age (IQR) was 46.5 years (38–55 years) and median BMI at baseline was 45.3 kg/m^2^ (41.7–51.0 kg/m^2^). Sixty-two (40%) participants reported no history of smoking, 69 (45%) previously smoked but quit before enrollment, and 23 (15%) smoked during the study period. Median baseline physical activity reported by IPAQ was 5055 (2389.5–10344.0) MET-min/week, and average daily calorie intake at baseline was 1661.0 kcal (1312.2–2211.7 kcal). Food diaries showed median daily intake of 73.0 g of protein (56.5–97.8 g), 54.2 g of mixed fats (38.1–74.2 g), and 228.1 g of simple and complex carbohydrates (173.8–302.1 g).

### 3.2. Baseline Sex-Specific Differences

Cross-sectional clinical characteristics of the study cohort’s 73 males and 81 females are described in Table 2. Although a one-way Mann–Whitney test showed that male age was significantly less than the female age ($\bar{x}$*_male_* = 44.3 < $\bar{x}$*_female_* = 47.8, *p* = 0.034), the average difference of 3.5 years was clinically slight, and the median test showed no significant difference (Table 2). Among anthropometric parameters at baseline, men had considerably greater total body mass, visceral adipose tissue mass (VAT), lean body mass, and muscle mass. However, there was no difference in BMI between the sexes, indicating that body size scaling (in the case of BMI, scaling by height) may diminish ostensible diffences between the sexes prior to surgery. There was also no difference in fat mass between men and women. Encouragingly, estimates of anthropometric parameters measured by DXA concorded with those measured by bioimpedence. At baseline, men consumed 40.3% more in total daily kcal than women in a diet that was 62.6% carbohydrate, 15.8% fat, and 21.6% protein (% total calories consumed). Although men consumed more of each nutrient category, women’s diet consisted of nearly identical prorpotions of carbohydrates, fat, and protein (64.2%, 15.3%, and 20.5%, respectively).

Among metabolic parameters, men had greater insulin concentration at the beginning of OGTTs as well as total insulin concentration throughout (insulin AUC), but no difference in glucose concentrations. To estimate beta-cell function and insulin resistance, we measured HOMA and Matsuda index parameters. Men had considerably higher HOMA- *β* and HOMA-IR, whereas women had greater Matsuda index values. Men also had considerably higher aspartate transaminase (AST) and alanine transaminase (ALT). There were no differences in lipid or cholesterol measurements except that men had 20.4% less HDL cholesterol than women. Model-adjusted estimates with quantile regression (Table S1) were not more than 15% different from arithmetic solutions in all cases.

### 3.3. Model Comparison

To test whether response curves to bariatric surgery occurred linearly or quadratically, we compared REMLs from our mixed models with and without quadratic terms, confirming these comparisons with nested hierarchical likelihood ratio tests (Supplementary Materials S1 and S2). In general, quadratic models were more likely, so were retained in subsequent analyses. These quadratic terms indicate that there is significant acceleration or deceleration in the response of most metabolic and anthropometric parameters over the 12-month follow-up period of this study. This implies that the length of our follow-up period is likely sufficient long to capture the bulk of the response to bariatric surgery.

Within quadratic models, we examined whether the inclusion of covariates (age, physical activity, diet, and sex) was more informative than their exclusion. In general, fully parameterized models including all covariates had lower REMLs than models without covariates. Since these covariates are known to affect most anthropometric and metabolic parameters, we retained fully parameterized models in downstream analyses so that we were confident that potential confounding by covariates was controlled for.

### 3.4. Cohort-Wide (Across-Sex) Responses to Bariatric Surgery

To understand how metabolic and anthropometric parameters changed under the sleeve gastrectomy across the entire cohort, we examined the magnitude and significance of time terms from our mixed models controlling for sex, age, diet, and physical activity (Table 3). For metabolic parameters, there were significant reductions in glucose loads during three of four time points of the OGTT, with glucose concentration at 30′ remaining unchanged. The 30′ measurement occurred relatively quickly after ingestion of the glucose, so it may not have represented sufficient time for glucose-clearing phsysiological machinery to act. Interestingly, mean glucose concentration during OGTT was not significantly changed (likely due to this steady high glucose measurement at 30′), but glucose AUC significantly decreased. Furthermore, insulin concentration was only significantly reduced at 120′ during OGTT. Contrary to glucose’s summary statistics, insulin’s mean was significantly reduced but its AUC was not.

Among non-glucose homeosatasis metabolic parameters, TG, AST, and ALT were significantly reduced while there were no significant changes in total cholesterol or other lipid measurements. Finally, as expected there were striking responses in anthropometric parameters reflecting decreased absolute and scaled body mass. Interestingly, there were significant losses in muscle mass (lean mass from DXA and muscle mass from bioimpedance) as well as fat.

### 3.5. Cross-Sectional (Sex-Specific) Responses to Bariatric Surgery

We tested for sex-specific responses by evaluating the significance of time × sex interactions in GLMMs fit to each response variable (Table 4). For metabolic parameters, there was a significant difference in the response of glucose load at 30′ during OGTT with men reduced more than women. Across-sex, there was no significant change in glucose concentration at 30′ (Table 2). Together, this indicates that men’s and women’s 30′ glucose response was countercurrent so that responses appeared null when averaged over the entire cohort but emerge when examining each sex specifically. There were no significant differences in the sex-specific response in other glucose-homeostasis parameters except for insulin concentration at 120′ during OGTT. Furthermore, men decreased HOMA-*β*, HOMA-IR, and Matsuda index more than women. For non-glucose homeostasis metabolic parameters, men increased HDL less than women but reduced AST and ALT more. Finally, men lost significantly more total mass and fat mass than women, though there were no significant sex-specific responses in other anthropometric parameters. 

**Figure 2:**
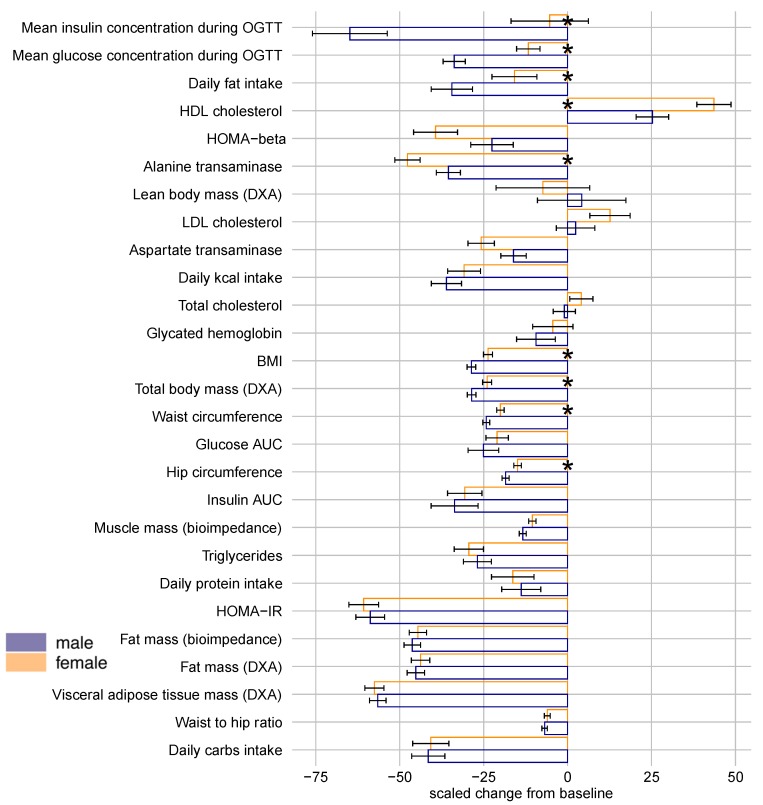
Model-adjusted means of responses scaled within individual patients from baseline to final follow-up visit (12 months post-surgery) adjusting for age and smoking status. Error bars are for standard errors. Asterisks indicate significant differences between sexes (*p* < 0.05). Ordered from the largest to the smallest difference between male and female mean response estimates. DXA—dual energy X-ray absorptiometry, HDL cholesterol—high-density lipoprotein cholesterol, LDL cholesterol—low-density lipoprotein cholesterol, OGTT—oral glucose tolerance test, BMI—body mass index, HOMA—homeostasis model assessment, AUC—area under the curve.

By examining model-adjusted means of scaled changes within individuals (Figure 2), we detected several additional parameters in which men improved more than women, specifically with greater reductions in BMI, hip circumference, and body weight. Furthermore, this analysis showed that the largest relative difference between male and female responses occurred in mean insulin and glucose concentrations during OGTT. This was followed by a greater reduction in fat consumption by men than women. Together, GLMMs and scaled responses indicate that men improved more than women in most anthropometric and metabolic parameters for which there was a significant difference between the sexes. 

**Figure 3:**
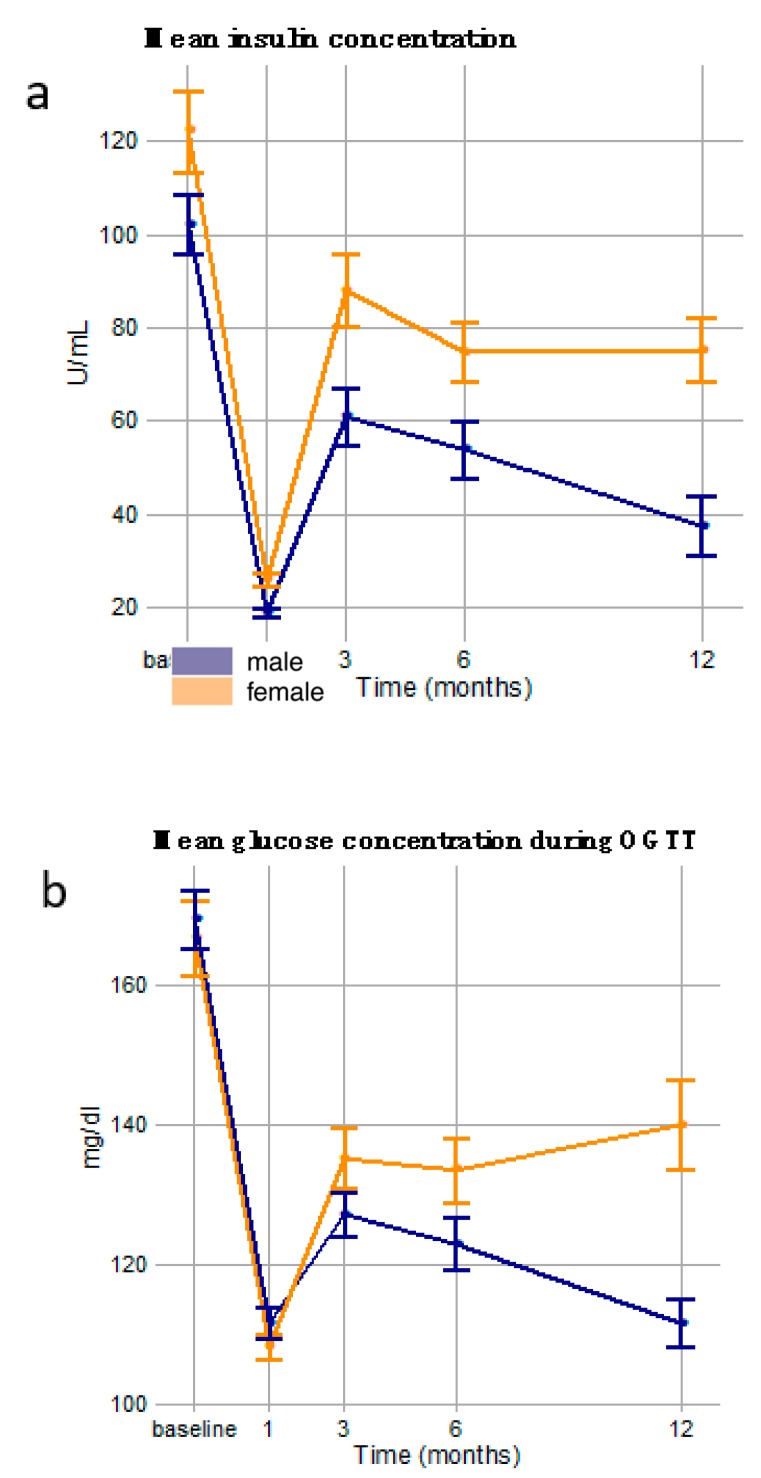
Two most sex-divergent long-term responses (i.e., with the largest difference in 12-month scaled responses between men and women), mean insulin (**a**) and glucose concentrations (**b**) during OGTT, from baseline to final follow-up exam. Points are model-adjusted means (see Methods). Dashed lines represent quadratic fits whose divergence is modeled by the generalized linear mixed models to test for significant time-by-sex interactions. Error bars are for standard errors.

Plotting values of the two most sex-divergent parameters (Figure 3) over the course of the study, mean insulin and mean glucose concentrations during OGTT, shows clear and sustained improvement in men, but much less improvement in women. Both show considerable improvements one month after surgery but rebound to glycemic derangement by three months. Although neither insulin nor glucose return to their original levels, the rebound is higher and more sustained for women but diminishes in men. In fact, the mean glucose concentration continues to rise in women whereas it falls in men.

### 3.6. Dysglycemia Diagnosis Differences in Sex-Specific Responses to Bariatric Surgery

**Figure 4:**
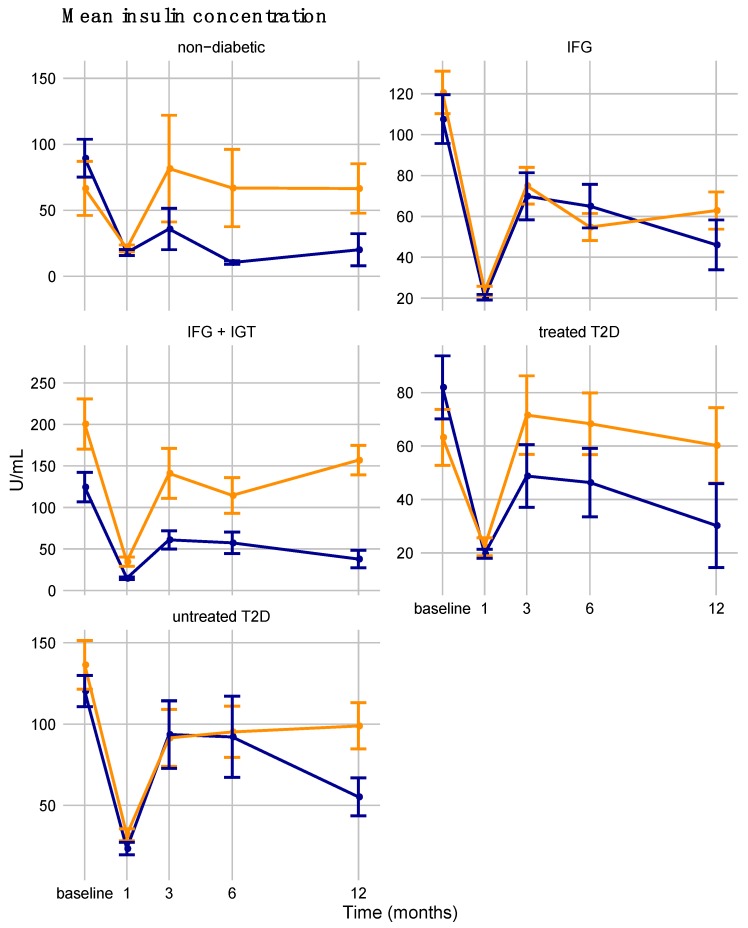
Cross-sectional representation of the sex-divergent long-term responses (i.e., with the largest difference in 12-month scaled responses between men and women) divided by dysglycemia diagnosis from baseline to final follow-up exam. IFG is for impaired fasting glucose, IGT for impaired glucose tolerance, and T2D for type 2 diabetes. Points are model-adjusted means (see Methods). Dashed lines represent quadratic fits. Error bars are for standard errors.

We were further interested in whether men and women with or without diabetes differed in their response to bariatric surgery. We tested for the significance of the three-way interaction among time, sex, and dysglycemia diagnosis for all parameters and none were significant. This is likely driven by the small sample size carried by each factor level combination when the cohort was stratified by both sex and diagnosis (e.g., only seven men were normoglycemic). We graphically examined the single most sex-divergent response, mean insulin concentration during OGTT, stratified by dysglycemia diagnosis (Figure 4) and observed that response patterns among the diagnosis groups were not strikingly different. Although intercepts differed considerably among diagnosis groups (for example, with untreated T2D being considerably higher than treated T2D), the responses to bariatric surgery among diagnosis groups were similar. Together with the failure of GLMMs to detect any significant three-way interactions, this indicates that sex did not noticeably impact the ameliorative effect of bariatric surgery depending on dysglycemia status. 

### 3.7. Sex-Spcific Trends in T2D Remission

**Figure 5:**
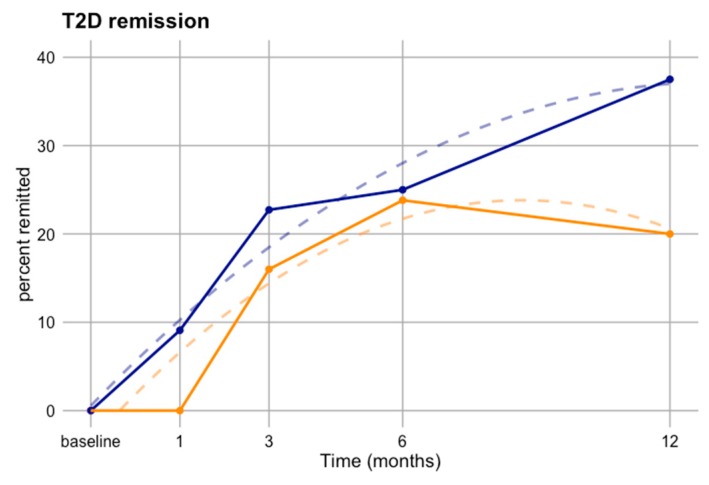
T2D remission percentage (number remitted/number with type 2 diabetes at baseline) in men and women from baseline to final follow-up exam. Dashed lines represent quadratic fits. T2D–type 2 diabetes.

At each follow-up visit, patients with diabetes were tested for T2D remission, and we asked whether remission occurred more strongly in men or women in response to bariatric surgery. Men remitted more quickly, more strongly, and more durably than women over the course of the study (Figure 5). In particular, by one month following surgery 9% of men with diabetes fully remitted while no women had. Although by three months 17.5% of women with diabetes had remitted, more men did at all subsequent time points. Women’s remission, on the other hand, peaked at six months post-surgery at 22% and then declined as several patients again showed diabetes symptoms. Thus, bariatric surgery appeared to induce remission more in men than women in our cohort.

## 4. Discussions

In this study, we evaluate sex-specific differences in the efficacy of sleeve gastrectomy to improve glucose homeostasis and anthropometric health. SG is the most frequently performed bariatric surgery worldwide, having surpassed RYGB in popularity, since its primary outcome (long-term weight loss) is equally efficacious, leads to fewer postoperative complications, and is technically less challenging and cheaper than RYGB [46,47,48,49]. This has been amply demonstrated by major randomized clinical trials including both the Swiss Multicenter Bypass or Sleeve Study [50] and the Finnish Sleeve versus Bypass Study [51]. These showed that total and excess weight loss at five years were not significantly different between SG and RYGB and that both procedures were similarly effective for diabetes resolution, sleep apnea and quality of life improvement. However, RYGB was also shown to be marginally more effective in patients with very high BMI (>50 kg/m^2^) and with poorly managed T2D, producing slightly greater weight loss and diabetes remission in these groups, respectively.

Furthermore, SG is an extremely safe surgical procedure, although not necessarily the safest bariatric procedure. For example, Chang et al. showed that adjustable gastric banding (AGB) was safest, followed by SG and RYGB (peri- and post-operative mortality rates, respectively: AGB = 0.07% and 0.21%, SG = 0.29% and 0.34% and RYGB = 0.38% and 0.72%, complications rates: AGB = 7.80%, SG = 8.90% and RYGB = 12.00% [11]). However, in our study, the complication rate calculated as the percentage of patients who experienced post-operative bleeding, stomal stenosis, leak, vomiting, reflux, gastrointestinal symptoms, or nutritional and electrolyte abnormalities was only 5.20%. This lower complication rate might be explained by our clinic’s strict implementation of guidelines to decrease post-operative morbidity, which have been shown to greatly reduce bariatric surgical complications [52]. For example, our patients were discharged with prescriptions for proton pump inhibitors (which reduce the risk of ulcer development and gastroesophageal reflux) so that these complications did not arise after the surgery. Taking together SG’s cost-effectiveness, procedural simplicity, and high rate of favorable outcomes, it is currently the first-line bariatric surgery, and this universality and popularity made it an ideal procedure to examine in this study.

The majority of previous studies that have sought to understand whether differences in SG efficacy are correlated with sex are based upon cohorts with unbalanced cross-sections of men and women (approximately 20% male and 80% female) [13,14,23,25,27]. Here, on the other hand, our sample is very close to sex balance (47% male and 53% female). Although this former proportion reflects the sex ratio of patients undergoing bariatric surgery in most clinics, it has little statistical utility when the goal is to estimate sex-specific effects—it simply allows more precise estimates of uncertainty about female effects [53]. Our balanced sample, on the other hand, eliminated bias that might arise from better estimates of uncertainty within subsamples of our cohort [54].

Furthermore, most studies examine cohorts in which men’s age is greater than women’s so that age may be an important mediating and potentially confounding variable in evaluations of sex-specific effects [13,14,23,25,27]. For example, a greater age is known to be associated with diminished weight loss after bariatric surgery [55]. In older people, adipose tissue deposition is increased and lipid metabolism is decreased, together constituting a metabolic sink which can be compensated by increasing food intake [56]. This increased consumption could greatly attenuate the benefit of bariatric surgery, and, if men are older, may appear to be related to sex. In this study, we decouple the potential confounding between age and sex since, although men consumed more total calories than women, they were not older. In fact, age was statistically significantly different only in a one-way non-parametric Mann–Whitney test which found that men were slightly younger than women (but no statistical difference was detected by the median test (Table 2). Furthermore, this difference was only 3.5 years (men = 44.3, women = 47.8), which likely has little clinical relevance. Since men and women in our cohort are largely matched for age and since we control for age in statistical analyses, we have a great deal of confidence that our sex-specific results are not confounded by age. This age matching was fortuitous since we did not implement any age filter in the inclusion or exclusion criteria, and we do not attempt to speculate as to why younger men sought bariatric surgery at our center than at many others.

Confounding, in general, is a significant issue in prospective studies and especially in studies that focus on sex since a constellation of cultural habits and physiological differences are correlated to sex and gender expression [57,58,59]. Previous studies have grappled with this issue by extensive exercises in model specification, adding and dropping terms and reporting how significance estimates change [13,14,23,25,27]. Here, we adopted a model selection approach by reporting all possible combinations of model covariates (see Supplementary Appendices S1 and S2), but our significance estimates were largely stable (e.g., time terms remained significant with different covariate combinations of age, diet, sex and physical activity). Although effect size magnitudes changed, their directions did not, and we base our conclusions upon the direction of change in response to bariatric surgery. Furthermore, we used formal model selection techniques (REML comparisons and likelihood ratio tests) to choose the most informative model, and this proved to be the most parameterized model for most response variables. This shows that covariates are vital to our analysis and that any inferences we draw from them must be, and were, adjusted for confounding differences between the sexes. 

By accounting for this confounding, we found that although men had greater absolute anthropometric metrics like total body mass and weight, there was no statistically significant difference in BMI. This implies that scaling by gross body size may largely equalize anthropometric morbidity in men and women. On the other hand, there was no statistically significant difference in fat mass between men and women. Since women were on average smaller than men (e.g., total body mass 23.5 kg less than men), women’s baseline fat content slightly higher than men’s (52.3% versus 45.6% fat mass as a percent of total body mass, respectively). Men also showed considerably greater derangement in glucose regulation with higher index values of insulin resistance and greater glucose load throughout OGTT, though no significant difference in HbA1c. This is in line with other studies that have found that men present with greater obesity-related comorbidities when undergoing surgery [23]. Men’s greater morbidity at baseline relative to women may account for previous findings that men improve more, since they have “further to go” when bariatric surgery normalizes deranged health axes.

To understand whether and how men and women responded differently to bariatric surgery, we tested for significant interactions between time and sex, examining both linear and quadratic time terms. A linear interaction term indicates that men increased or decreased more than women averaging over all follow-up visits, while the quadratic interaction term indicates whether there was significant acceleration or deceleration in this trend (i.e., is there significant curvature in the response). We detected 11 significant linear interactions with six also showing significant quadratic interactions (Table 3). All of these interactions indicated that men improved more than women over the study period with greater decreases in glucose and insulin loads, improved insulin sensitivity and beta-cell function and greater body mass and fat reduction. Significant quadratic interactions indicated that the deceleration or acceleration in response was more pronounced in men than women. For example, men’s response in mean insulin concentration curved more strongly (Figure 3a), implying that men’s downward trend may have continued more durably than the deceleration in women’s response. However, this is a tentative conclusion that requires further follow-up to determine whether men’s greater improvement is sustained over a period longer than one year.

Another way to examine responses is to scale within individual patients and evaluate means from scaled differences (Figure 2), and this showed remarkable divergence in the response of mean insulin and glucose concentration between men and women. Indeed, men showed much greater decreases in these means, although evaluating each timepoint individually within the four-point OGTT by the GLMM revealed a sex-specific effect only at 30′ (Table 3). This is the most proximate measurement to the introduction of the oral glucose bolus to the patient, representing the peak glucose load experienced by the patient. Following bariatric surgery, men’s ability to clear this peak glucose apparently improved more than women’s, and this translated into an overall greater decrease in mean glucose load. Interestingly, there was no significant difference in HbA1c, which gauges average blood glucose levels over three months. Together, this shows that bariatric surgery may resolve men’s acute glucose derangement (occurring on the scale of minutes after glucose exposure) more than women’s, but that their longer-term responses may not be as divergent.

There were several limitations to our study. Our patient cohort consisted entirely of Polish Caucasians, and was not large (*n* = 154). On the other hand, by limiting our sample to a specific national group we eliminated confounding variables that may covary with nationality. Similarly, there were relatively few patients with diabetes at baseline (*n* = 47, 31%), so that our analysis of sex-specific remission differences is not as robust as across-diagnosis analyses. Finally, our nutritional data are based upon self-reported food diaries analyzed by technicians and thus may suffer from the systematic biases of self-reported consumption. However, all subjects were educated by trained dieticians in how to maintain detailed, accurate, and unbiased diet diaries.

## 5. Conclusions

We found that sleeve gastrectomy improved glucose homeostasis and anthropometric parameters more in men than women, that this is likely related to men’s greater morbidity at baseline, and that this greater morbidity is independent of age. In particular, men decreased in fat and absolute body mass more than women and showed more pronounced decreases in metrics of dysglycemia, especially in scaled metrics like the Matsuda and HOMA indices. At baseline, men showed greater dysregulation in glucose homeostasis for scaled metrics, but their greater obesity-related morbidity did not endure scaling (e.g., men’s total body mass was greater than women’s, but not BMI). Together, this indicates that men’s greater improvement in anthropometric parameters may be related to men’s greater gross bodyweight but that their improvement in glucose homeostasis reflects size-independent physiological normalization. Finally, the follow-up period of this study occurred over one year. Although the significance of quadratic time terms indicated plateauing responses, these results point to the need for longer-term follow-up to determine whether the sex specificity of these results is durable.

## Figures and Tables

**Table 1:** Measurements gathered in this study divided into metabolic, anthropometric, and nutritional categories.

Metabolic	Anthropometric
4-point glucose, OGTT	Waist-to-hip ratio
4-point insulin, OGTT	Total body mass
Glycated hemoglobin (HbA1c)	Lean body mass (DXA)
HOMA-β	Visceral adipose tissue mass (DXA)
HOMA-IR	Fat mass (DXA)
Matsuda index	Fat mass (bioimpedance)
Total cholesterol	Skeletal muscle mass (bioimpedance)
Triglycerides	BMI
HDL cholesterol	**Nutritional/Lifestyle**
LDL cholesterol	Total Daily Calories
Aspartate transaminase (AST)	Carbohydrate mass-consumed
Alanine transaminase (ALT)	Fat mass-consumed
	Protein mass-consumed
	Physical activity (IPAQ)

Glucose and insulin concentrations were measured at four time points during the oral glucose tolerance test (0, 30, 60, and 120 min) and then used to calculate their area-under-the-curves. OGTT—oral glucose tolerance test, DXA—dual-energy X-ray absorptiometry, BMI—body mass index, IPAQ—international physical activity questionnaire.

**Table 2:** Baseline characteristics for the study cohort cross-sectioned by sex.

Parameter (Unit)	Male	Female	*p*
N	73 (47)	81 (53)	NA
Age	44 (34–54)	48 (40–55)	0.109
Never Smoked	25 (34)	37 (46)	NA
Positive History of Smoking	37 (51)	32 (40)	NA
Currently Smoking	11 (15)	12 (15)	NA
FH T2D	27 (37)	28 (35)	NA
FH Obesity	52 (71)	70 (86)	NA
Dysglycemia diagnosis: non-diabetic	7 (10)	9 (11)	NA
Dysglycemia diagnosis: IFG	29 (40)	29 (36)	NA
Dysglycemia diagnosis: IFG + IGT	11 (15)	15 (19)	NA
Dysglycemia diagnosis: untreated T2D	11 (15)	9 (11)	NA
Dysglycemia diagnosis: treated T2D	11 (15)	16 (20)	NA
Total body mass (kg)	145 (135.6–160.6)	121.5 (107.95–139.4)	**<0.001**
Fat mass (DXA) (kg)	66.2 (56.3–77.6)	63.6 (53.2–71.4)	0.249
Lean body mass (DXA) (kg)	77.0 (72.9–83.8)	56.5 (51.5–62.6)	**<0.001**
Visceral adipose tissue mass (DXA) (kg)	5.1 (4.2–5.6)	2.5 (2.1–3.4)	**<0.001**
Muscle mass (bioimpedance) (kg)	46.65 (43.68–51.12)	33.6 (30.3–37.3)	**<0.001**
Fat mass (bioimpedance) (kg)	67.7 (57.82–79.38)	63.9 (53.8–72.7)	0.230
BMI (kg/m^2^)	46.18 (43.38–51.49)	44.54 (39.76–49.62)	0.295
Daily kcal intake (kcal)	2072.37 (1465.93–2461.11)	1477.02 (1204–1935.54)	**0.023**
Daily protein intake (g)	89.9 (67.94–115.95)	67.47 (53.49–80.18)	**<0.001**
Daily fat intake (g)	65.79 (45.23–85.01)	50.41 (35.36–67.46)	**0.027**
Daily carbs intake (g)	260.13 (187.69–357.19)	211.38 (163.08–261.59)	**0.012**
Glucose at 0 min of OGTT (mg/dL	114 (107–134)	114 (106–127)	0.766
Insulin at 0 min of OGTT (U/mL)	37.42 (27.31–51.44)	23.86 (16.68–31.89)	**<0.001**
Glucose at 120 min of OGTT (mg/dL)	133 (112.5–188.5)	140 (112–182.5)	0.485
Insulin at 120 min of OGTT (U/mL)	107.8 (60.7–170.4)	94.4 (52.7–158.2)	0.201
Glycated hemoglobin (HbA1c) (%)	5.9 (5.5–6.5)	5.8 (5.5–6.35)	0.509
Mean insulin concentration during OGTT (U/mL)	116.08 (64.24–159.83)	91.81 (66.8–123.84)	0.076
Mean glucose concentration during OGTT (mg/dL)	156 (138–193)	160.5 (138.25–196.25)	0.259
Matsuda index	1.16 (0.73–1.94)	1.6 (1–2.31)	**0.002**
Glucose AUC	339.5 (307–422.5)	344.25 (296.75–422.25)	0.864
Insulin AUC	272.88 (205.3–392.15)	231.01 (162.66–319.8)	**0.021**
HOMA- β	236.35 (168.95–350.78)	160.16 (112.94–223.94)	**<0.001**
HOMA-IR	11.38 (7.8–16.09)	6.76 (4.54–9.83)	**<0.001**
Total cholesterol (mg/dL)	192 (160–219)	191 (165–223)	0.872
Triglycerides (mg/dL)	143 (114–189)	135 (99–167)	0.259
HDL cholesterol (mg/dL)	39 (34–45)	49 (41–57)	**<0.001**
LDL cholesterol (mg/dL)	122 (95–143)	120 (97–145)	0.872
Aspartate transaminase (U/L)	27.5 (22.1–35.8)	20.5 (17.2–26.2)	**<0.001**
Alanine transaminase (U/L)	42 (32.6–55.3)	25.2 (19–31.9)	**<0.001**
Physical activity (METs- min/week)	5772 (2590–10,314)	4227 (2292–11,257)	0.167

Continuous parameters are presented as medians (IQR) and discrete data as the sample size (%). *p* is for *p*-value for non-parametric tests of median differences between male and female. NAs are for discrete parameters to which median tests do not apply. Bolded values indicate significant differences (*p* < 0.05). N–number of subjects, FH—family history, T2D—type 2 diabetes, IFG—impaired fasting glucose, IGT—impaired glucose tolerance, DXA—dual-energy X-ray absorptiometry, HDL cholesterol—high-density lipoprotein cholesterol, LDL cholesterol—low-density lipoprotein cholesterol, OGTT—oral glucose tolerance test, BMI—body mass index, HOMA—homeostasis model assessment, AUC—area under the curve, MET—metabolic equivalent of task.

**Table 3:** Time coefficients from GLMMs and significances adjusting for age, sex, diet, and physical activity.

	Time	Time ^2^		Time	Time ^2^
[glucose] 0′ (OGTT)	−2.624 ***	0.152 **	waist circumference	−5.246 ***	0.240 ***
[glucose] 30′ (OGTT)	−1.931	0.073	hip circumference	−4.312 ***	0.195 ***
[glucose] 60′ (OGTT)	−7.784 **	0.440 *	waist-to-hip ratio	−0.011 ***	0.000 *
[glucose] 120′ (OGTT)	−12.152 ***	0.673 ***	total body mass	−6.212 ***	0.304 ***
[insulin] 0′ (OGTT)	−0.812	0.037	fat mass (DXA)	−4924.935 ***	229.342 ***
[insulin] 30′ (OGTT)	5.864	−0.267	lean body mass (DXA)	−794.795 ***	36.214 ***
[insulin] 60′ (OGTT)	1.407	−0.245	visceral adipose (DXA)	−277.855 ***	13.527 ***
[insulin] 120′ (OGTT)	−13.138 **	0.593	muscle mass (bio.)	−0.758 ***	0.038 ***
HbA1c	−0.082 ***	0.005 **	fat mass (bio.)	−5.330 ***	0.262 ***
HOMA- β	−0.593	0.031	BMI	−2.328 ***	0.115 ***
HOMA-IR	−0.353 *	0.018	BMI change	2.057 ***	−0.100 ***
mean [insulin] (OGTT)	7.904 **	−0.706 ***	EBMIL	10.142 ***	−0.492 ***
mean [glucose] (OGTT)	−0.61	−0.081	total weight loss	4.456 ***	−0.216 ***
Matsuda index	0.631 **	−0.026	excess weight loss	10.093 ***	−0.490 ***
glucose AUC (OGTT)	−13.797 ***	0.783 **	HDL cholesterol	0.419	0.053
insulin AUC (OGTT)	−3.591	0.112	LDL cholesterol	1.804	−0.118
total cholesterol	0.421	−0.019	Aspartate transaminase	−1.421 ***	0.080 *
triglycerides	−5.441 *	0.256	Alanine transaminase	−3.256 **	0.184 *

Sex coefficients indicate male direction. Bolded numbers indicate significance (*p* < 0.05). Asterisks indicate significance order of magnitude: * *p* = [0.05,0.01], ** *p* = [0.01,0.001], and *** *p* < 0.001. DXA—dual energy X-ray absorptiometry, bio.—bioimpedance, HDL cholesterol—high-density lipoprotein cholesterol, LDL cholesterol—low-density lipoprotein cholesterol, OGTT—oral glucose tolerance test, BMI—body mass index, HOMA—homeostasis model assessment, AUC—area under the curve, EBMIL—excess body mass index loss.

**Table 4:** Time × sex coefficients from GLMMs and significances adjusting for age, sex, diet, and physical activity.

	Time × Sex	Time^2^ × Sex		Time × Sex	Time^2^ × Sex
[glucose] 0′ (OGTT)	−1.323	0.114	waist circumference	0.117	−0.009
[glucose] 30′ (OGTT)	−4.457 *	0.383 *	hip circumference	0.321	0.002
[glucose] 60′ (OGTT)	−2.46	0.203	waist-to-hip ratio	0.001	0
[glucose] 120′ (OGTT)	−2.588	0.188	total body mass	−1.149 *	0.073
[insulin] 0′ (OGTT)	−3.128 *	0.179	fat mass (DXA)	−1105.802 *	69.935
[insulin] 30′ (OGTT)	−2.464	−0.072	lean body mass (DXA)	−355.734	30.562
[insulin] 60′ (OGTT)	−5.767	0.449	visceral adipose (DXA)	−307.185 ***	17.030 ***
[insulin] 120′ (OGTT)	−16.033 **	1.170 *	muscle mass (bio.)	−0.051	0.006
HbA1c	−0.025	0.001	fat mass (bio.)	−1.263 **	0.074 *
HOMA-beta	−10.117 *	0.515	BMI	0.06	0
HOMA-IR	−1.061 ***	0.070 ***	BMI change	−0.136	0.005
mean [insulin] (OGTT)	1.055	0.026	EBMIL	−1.382	0.058
mean [glucose] (OGTT)	1.365	0.006	total weight loss	−0.383	0.013
Matsuda index	−0.672 **	0.031	excess weight loss	−1.475	0.056
glucose AUC (OGTT)	−5.272	0.419	HDL cholesterol	1.198 *	−0.086
insulin AUC (OGTT)	−13.561	0.816	LDL cholesterol	0.011	0.012
Total cholesterol	−1.602	0.107	Aspartate transaminase	−1.383 *	0.093 *
triglycerides	−6.03	0.374	Alanine transaminase	−3.789 *	0.219

Sex coefficients indicate male direction. Bolded numbers indicate significance (*p* < 0.05). Asterisks indicate significance order of magnitude: * *p* = [0.05,0.01], ** *p* = [0.01,0.001], and *** *p* < 0.001. DXA—dual energy X-ray absorptiometry, bio.—bioimpedance, HDL cholesterol—high-density lipoprotein cholesterol, LDL cholesterol—low-density lipoprotein cholesterol, OGTT—oral glucose tolerance test, BMI—body mass index, HOMA—homeostasis model assessment, AUC—area under the curve, EBMIL—excess body mass index loss.

## Supplementary Materials

The following are available online at https://www.mdpi.com/2072-6643/11/10/2408/s1, Table S1: quantile regression results; Appendix S1: anthropometric linear and quadratic GLMM results with all covariate combinations; Appendix S2: metabolic linear and quadratic GLMM results with all covariate combinations.
