# Supplementary material for: Sex-Specific Glucose Homeostasis and Anthropometric Responses to Sleeve Gastrectomy in Obese Patients
Source: Nutrients. 2019 Oct 9;11(10):2408. doi: 10.3390/nu11102408 (PMC6836106; doi:10.3390/nu11102408)
Supplement: Supplementary file 1 [file nutrients-11-02408-s001.pdf]

Supplementary Materials for

Sex-specific glucose homeostasis and anthropometric responses to sleeve gastrectomy in obese patients

Mark A. Taylor, Lukasz Szczerbinski , Anna Citko, Magdalena Niemira, Maria Gorska, Hady Razak Hady and Adam Kretowski

Author for correspondence : Lukasz Szczerbinski
Email: lukasz.szczerbinski@umb.edu.pl

Table S1 Quantile regression for response parameters. We implemented three of five possible quantile regression models to estimate median and IQR adjusted for: 1) age by model 1; 2) age and smoking status by model 2; 3) age, smoking status, baseline diet (total kCal intake), and baseline IPAQ by model 3; 4) age, smoking status, and baseline BMI by my model 4; and 5) age, smoking status, baseline BMI, baseline diet, and baseline IPAQ by model 5. Models 1, 2, and 3 were used for continuous variables based upon or highly correlated to BMI (such as weight), while models 1, 4, and 5 were used for all others

parameter (unit)	model	male	female
Total body mass (DXA) (kg)		145 (135.6-160.6)	121.5 (107.95-139.4)
	1	143.08 (139.43-146.37)	121.04 (116.71-126.29)
	2	143.06 (139.73-146.64)	119.27 (114.7-123.39)
	3	146.54 (139.26-154.13)	120.73 (114.19-126.22)
Fat mass (DXA) (g)		66181 (56380-77634)	63613 (53221-71379)
	1	66015.52 (63534.65-68248.3)	61563.2 (57810.92-66119.54)
	2	65794.41 (63259.59-67906.76)	61518.38 (57858.56-64106)
	3	67604.26 (62543.18-72295.38)	61278.26 (56677.64-64365.77)
Lean body mass (DXA) (g)		77020 (72785-83776)	56492 (51464.5-62619)
	1	78053.89 (76407.05-79536.05)	56290.2 (55140.8-57685.9)
	2	77795.47 (76057.81-79243.53)	55942.86 (55146.57-57660.61)
	3	77195.21 (75106.82-78950.18)	55314.04 (53467.38-57172.79)
Visceral adipose tissue mass (DXA) (g)		5116 (4198-5644)	2510 (2065.75-3364.5)
	1	5549.73 (4897.73-6083.18)	2681 (2476.19-2885.81)
	2	5544.39 (4886.71-6082.5)	2597.8 (2398.33-2819.13)
	3	5408.48 (4930.23-5771.83)	2649.43 (2505.75-2847.43)
Body weight (kg)		146.75 (136.05-162.97)	122.8 (108-139.3)
	1	145.24 (140.35-149.9)	122.34 (119.02-126.13)
	2	145.44 (139.41-149.61)	119.21 (115.15-122.9)
	3	154.22 (145.46-162.37)	120.71 (113.94-126.19)
BMI (kg/m^2)		46.18 (43.38-51.49)	44.54 (39.76-49.62)
	1	46.3 (45.95-46.62)	44.33 (43.9-44.81)
	2	46.47 (46.23-47.29)	44.21 (42.9-44.87)
	3	47.98 (46.03-49.47)	44.16 (43.37-45.27)
Leptin (ng/ml)		30.09 (20.73-47.36)	51.97 (38.28-68.48)
	1	30.79 (26.66-34.65)	50.56 (48.8-51.77)
	2	32.13 (27.39-36.52)	49.59 (46.92-52.31)
	3	35.29 (31.97-41.08)	50.03 (47.04-56.26)
Daily kcal intake (kcal)		2072.37 (1465.93-2461.11)	1477.02 (1204.74-1935.54)
	1	1874.35 (1649.42-2109.5)	1474.67 (1470.56-1478.77)
	2	1887.56 (1668.34-2172.21)	1476.14 (1470.04-1483.12)
	3	2024.62 (1464.02-2449.58)	1465.76 (1202.66-1952.08)
Daily protein intake (g)		89.9 (67.94-115.95)	67.47 (53.49-80.18)
	1	86.91 (79.88-94.27)	68.95 (67.8-70.11)
	2	91.47 (83.9-99.59)	67.87 (64.41-69.02)
	3	85.3 (70.29-105.11)	66.42 (55.48-81.81)
Daily fat intake (g)		65.79 (45.23-85.01)	50.41 (35.36-67.46)

	1	59.59 (54.44-64.97)	49.49 (48.4-50.57)
	2	65.06 (57.46-73.86)	49.57 (48.53-50.51)
	3	66.37 (46.08-86.16)	49.81 (37.13-68.27)
Daily carbs intake (g)		260.13 (187.69-357.19)	211.38 (163.08-261.59)
	1	254.75 (224.8-286.05)	209.07 (198.89-219.24)
	2	251.56 (223.77-279.71)	209.32 (197.75-217.5)
	3	276.6 (203.09-342.7)	205.78 (177.82-260.99)
Glucose at 0 minute of OGTT (mg/dl)		114 (107-134)	114 (106-127)
	1	117.89 (110.67-125.11)	114.44 (110-118.33)
	4	116.26 (110.61-122.73)	115.25 (110.24-121.24)
	5	118.97 (110.99-126.04)	116.43 (110.66-121.55)
Insulin at 0 minute of OGTT (U/mL)		37.42 (27.31-51.44)	23.86 (16.68-31.89)
	1	37.7 (35.57-39.84)	23.9 (23.43-24.44)
	4	38.51 (34.05-43.83)	22.18 (20.19-25.21)
	5	36.9 (28.9-44.6)	23.82 (21.47-25.82)
Glycated hemoglobin (%)		5.9 (5.5-6.5)	5.8 (5.5-6.35)
	1	5.86 (5.77-5.96)	5.84 (5.72-5.95)
	4	5.87 (5.7-5.96)	5.79 (5.67-5.88)
	5	5.96 (5.77-6.12)	5.98 (5.76-6.06)
HOMA-beta		236.35 (168.95-350.78)	160.16 (112.94-223.94)
	1	252.43 (207.73-297.14)	158.95 (144.35-175.63)
	4	246.18 (210.23-287.72)	160.47 (142.3-177.05)
	5	247.53 (202.51-319.14)	157.98 (133.47-181.6)
HOMA-IR		11.38 (7.8-16.09)	6.76 (4.54-9.83)
	1	11.68 (11.19-12.17)	7.36 (7.09-7.6)
	4	11.83 (10.43-12.73)	6.41 (5.65-7.56)
	5	11.92 (9.93-13.68)	7.2 (6.54-7.42)
Total Cholesterol(mg/dl)		192 (160-219)	191 (165-223)
	1	186.62 (181.23-192)	191.2 (186.4-195.4)
	4	188.89 (186.64-191.57)	189.15 (184.2-194.69)
	5	182.96 (175.81-193.64)	186.64 (178.74-200.57)
Triglycerides (mg/dl)		143 (114-189)	135 (99-167)
	1	143.86 (134.79-152.93)	130 (122-137)
	4	144.64 (131.56-156.05)	135.05 (124.71-145.04)
	5	154.55 (136.46-180.95)	129.06 (121.5-137.37)
HDL Cholesterol(mg/dl)		39 (34-45)	49 (41-57)
	1	38.85 (37.1-40.6)	49.4 (46.84-51.64)
	4	39.15 (37.89-40.85)	50.41 (46.12-53.39)
	5	38.16 (36.43-40.71)	49.14 (46.81-50.84)
LDL Cholesterol(mg/dl)		122 (95-143)	120 (97-145)
	1	123.03 (119.37-126.7)	119.91 (119.16-120.77)
	4	122.76 (119.9-125.09)	116.42 (111-119.66)
	5	116.79 (108.48-124.75)	118.34 (113.39-125.14)
Aspartate transaminase (U/l)		27.5 (22.1-35.8)	20.5 (17.2-26.2)
	1	26.85 (25.44-28.26)	20.54 (19.7-21.27)
	4	26.14 (24.11-28.02)	20.43 (19.63-21.3)
	5	26.49 (25.12-28.16)	19.75 (18.94-20.47)
Alanine transaminase (U/l)		42 (32.6-55.3)	25.2 (19-31.9)
	1	43.53 (38.07-49)	25.45 (23.87-26.83)
	4	42.67 (38.01-48.24)	25.24 (23.4-27.92)
	5	42.79 (37.54-48.54)	24.92 (23.96-25.79)

C Reactive Protein (mg/l)		5.15 (3.14-9.65)	6.25 (3.28-9.61)
	1	5.4 (4.55-6.26)	5.96 (4.9-7.17)
	4	5.21 (4.61-6)	6.02 (4.37-7.32)
	5	5.19 (4.28-6.33)	6.31 (4.99-7.66)
Physical activity (METs- minutes/week)		5772 (2590.5-10314)	4227 (2292-11257.5)
	1	6612.47 (5485.11-7686.16)	4687.7 (3938.06-5597.99)
	4	6569.88 (5572.05-7814.08)	4558.92 (3227.18-5630.87)
	5	6148.5 (2539.5-10374)	4242 (2524.5-11257.5)
Mean insulin concentration during OGTT (U/mL)		116.08 (64.24-159.83)	91.81 (66.8-123.84)
	1	112.78 (105.21-120.35)	87.06 (81.58-93.33)
	4	114.07 (97.57-132.97)	86.01 (80.57-95.15)
	5	108.68 (92.49-125.4)	86.74 (77.91-99.97)
Mean glucose concentration during OGTT (mg/dl)		156 (138-193)	160.5 (138.25-196.25)
	1	157.38 (149.56-165.19)	162.88 (149.59-174.51)
	4	156.5 (148.52-165.01)	165.51 (153.45-177.91)
	5	160.02 (149.21-167.3)	166.16 (152.53-182.24)
MATSUDA index		1.16 (0.73-1.94)	1.6 (1-2.31)
	1	1.18 (1.15-1.2)	1.58 (1.53-1.64)
	4	1.14 (0.98-1.27)	1.74 (1.53-1.84)
	5	1.12 (0.87-1.27)	1.67 (1.49-1.79)
Glucose AUC		339.5 (307-422.5)	344.25 (296.75-422.25)
	1	334.08 (316.04-357.27)	352.06 (320.6-379.59)
	4	331.52 (316.64-352.47)	351.98 (327.65-379.52)
	5	343.06 (320.54-364.06)	356.17 (328-380.57)
Insulin AUC		272.88 (205.3-392.15)	231.01 (162.66-319.8)
	1	275.83 (268.63-281.43)	215.25 (201.83-230.11)
	4	267.27 (238.58-299.63)	213.81 (202.3-238.06)
	5	280.69 (222.62-346.31)	208.18 (188.93-234.47)

Supplementary Appendix 1: Anthropometric Generalized Linear Mixed Models

Table 1. Results of linear models for waist circumference 6
Table 2. Results of quadratic models for waist circumference 7
Table 3. Results of linear models for hip circumference 8
Table 4. Results of quadratic models for hip circumference 9
Table 5. Results of linear models for WAIST-TO-HIP RATIO (WHR) 10
Table 6. Results of quadratic models for WAIST-TO-HIP RATIO (WHR) 11
Table 7. Results of linear models for Total body mass (DXA) 12
Table 8. Results of quadratic models for Total body mass (DXA) 13
Table 9. Results of linear models for Fat mass (DXA) 14
Table 10. Results of quadratic models for Fat mass (DXA) 15
Table 11. Results of linear models for Lean mass (DXA) 16
Table 12. Results of quadratic models for Lean mass (DXA) 17
Table 13. Results of linear models for Visceral adipose tissue mass (DXA) 18
Table 14. Results of quadratic models for Visceral adipose tissue mass (DXA) 19
Table 15. Results of linear models for Total body mass (bioimpedence) 20
Table 16. Results of quadratic models for Total body mass (bioimpedence) 21
Table 17. Results of linear models for Muscle mass (bioimpedence) 22
Table 18. Results of quadratic models for Muscle mass (bioimpedence) 23
Table 19. Results of linear models for Fat mass (bioimpedence) 24
Table 20. Results of quadratic models for Fat mass (bioimpedence) 25
Table 21. Results of linear models for BMI 26
Table 22. Results of quadratic models for BMI 27

waist circumference

Figure 1. Distribution of waist circumference in time - linear fit

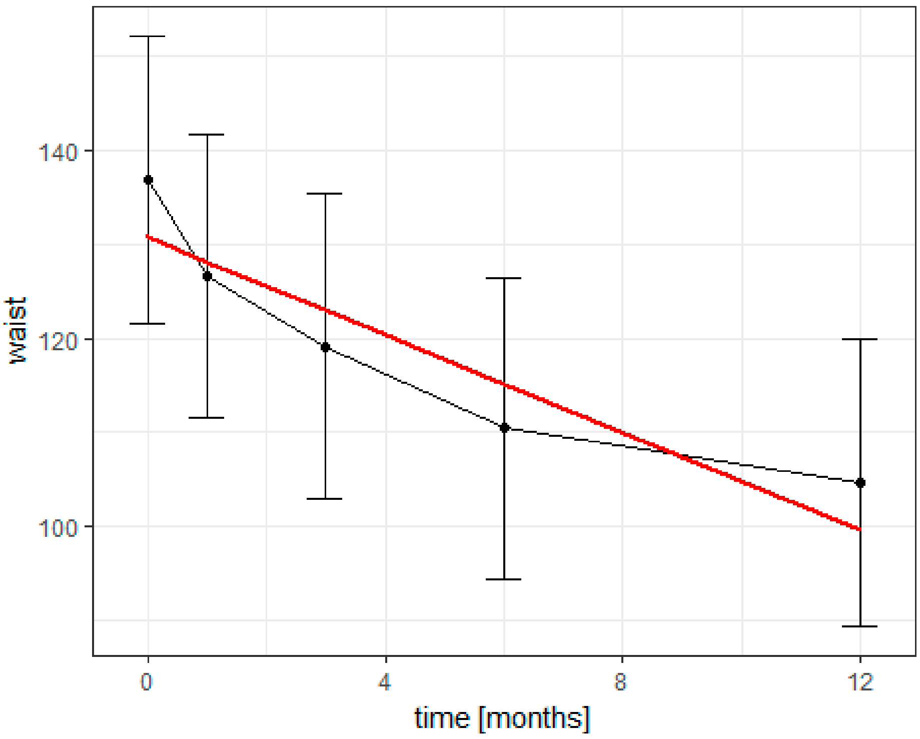

Table 1. Results of linear models for waist circumference

variable	without covariates	model 1	model 2	model 3	model 4	model 5	model 6	model 7
(Intercept)	130.8***	130.9***	125.6***	125.4***	125.8***	125.2***	120.8***	120.8***
time	-2.593***	-2.554***	-2.596***	-2.465***	-2.553***	-2.402***	-2.469***	-2.405***
IPAQ		-0.000			-0.000	-0.000		-0.000
sexM			11.374***		11.459***		10.118***	10.008***
diet_kcal				0.005***		0.005***	0.005***	0.005***
no. of observations	596	576	596	558	576	540	558	540
REML	4511.3	4384.3	4485.2	4159.1	4356.8	4040.1	4137.7	4018.4

Figure 2. Distribution of waist circumference in time - quadratic fit

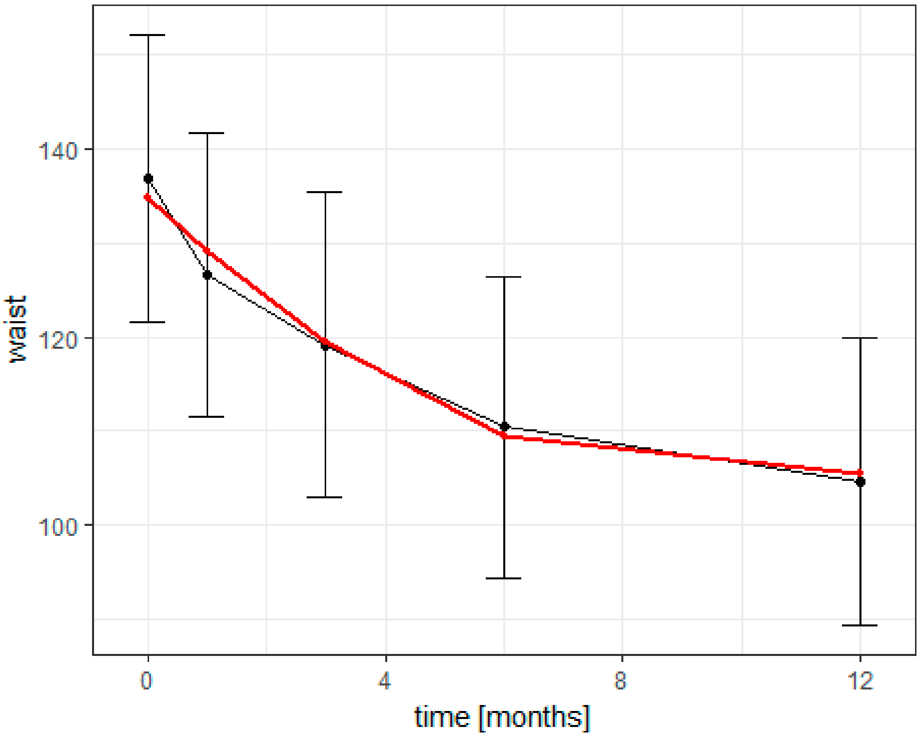

Table 2. Results of quadratic models for waist circumference

variable	without covariates	model 1	model 2	model 3	model 4	model 5	model 6	model 7
(Intercept)	134.8***	134.8***	129.6***	131.2***	129.6***	130.8***	126.4***	126.1***
time_sq	0.299***	0.298***	0.299***	0.243***	0.298***	0.234***	0.245***	0.236***
time	-6.026***	- 5.999***	-6.031***	- 5.323***	-5.999***	- 5.166***	-5.351***	-5.195***
IPAQ		-0.000			-0.000	-0.000		-0.000
sexM			11.468***		11.516***		10.776***	10.686***
diet_kcal				0.003***		0.003***	0.003***	0.003***
no. of observations	596	576	596	558	576	540	558	540
REML	4308.9	4194.1	4282.4	4043.2	4166.5	3938.6	4019.5	3914.5

hip circumference

Figure 3. Distribution of hip circumference in time - linear fit

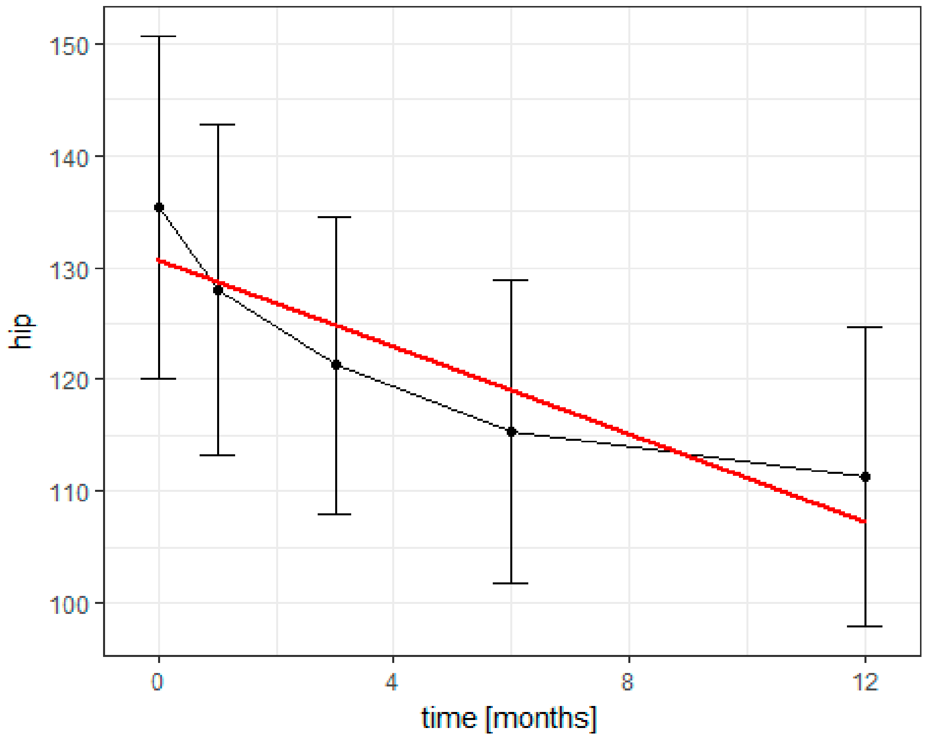

Table 3. Results of linear models for hip circumference

variable	without covariates	model 1	model 2	model 3	model 4	model 5	model 6	model 7
(Intercept)	130.7***	130.4***	131.3***	127.1***	131***	126.3***	128.1***	127.3***
time	-1.964***	-1.937***	-1.963***	-1.886***	-1.937***	-1.860***	-1.886***	-1.859***
IPAQ		0.000			0.000	0.000		0.000
sexM			-1.327		-1.206		-2.329	-2.271
diet_kcal				0.004***		0.004***	0.004***	0.004***
no. of observations	596	576	596	558	576	540	558	540
REML	4364.9	4230	4361.1	4055.5	4226.2	3934.5	4050.9	3930

Figure 4. Distribution of hip circumference in time - quadratic fit

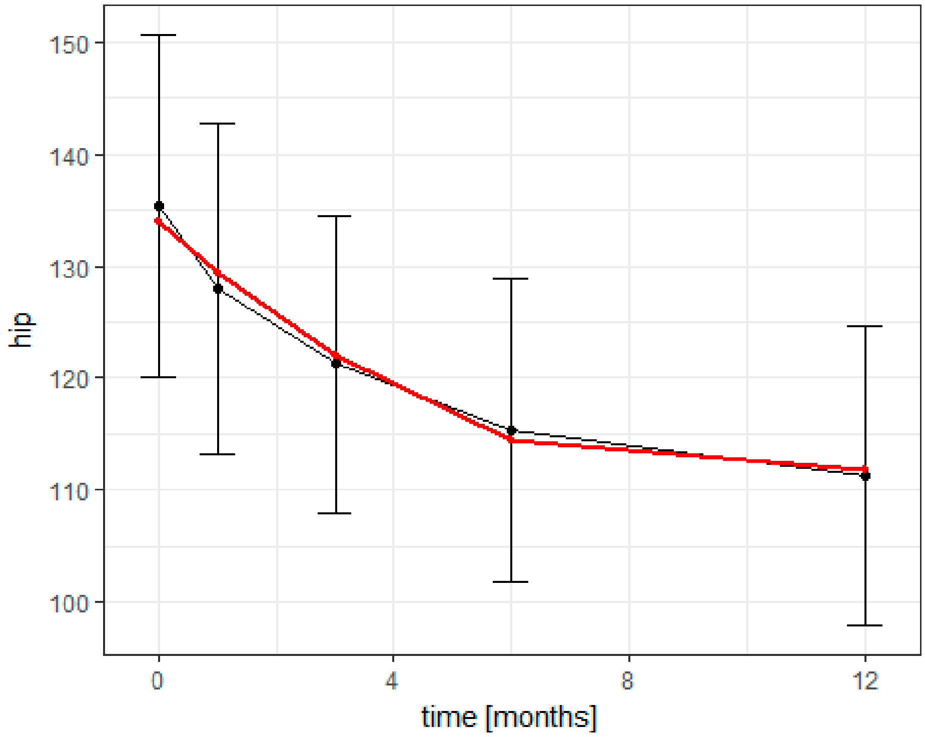

Table 4. Results of quadratic models for hip circumference

variable	without covariates	model 1	model 2	model 3	model 4	model 5	model 6	model 7
(Intercept)	133.9***	133.5***	134.5***	131.9***	134***	131***	132.7***	131.8***
time_sq	0.236***	0.234***	0.236***	0.202***	0.234***	0.196***	0.202***	0.196***
time	-4.676***	- 4.644***	- 4.675***	- 4.264***	- 4.644***	- 4.184***	- 4.259***	- 4.179***
IPAQ		0.000			0.000	0.000		0.000
sexM			-1.247		-1.155		-1.783	-1.700
diet_kcal				0.002***		0.002***	0.002***	0.002***
no. of observations	596	576	596	558	576	540	558	540
REML	4209.8	4077.8	4206	3961.2	4074	3847.2	3957.1	3843.1

WAIST-TO-HIP RATIO (WHR)

Figure 5. Distribution of WAIST-TO-HIP RATIO (WHR) in time - linear fit

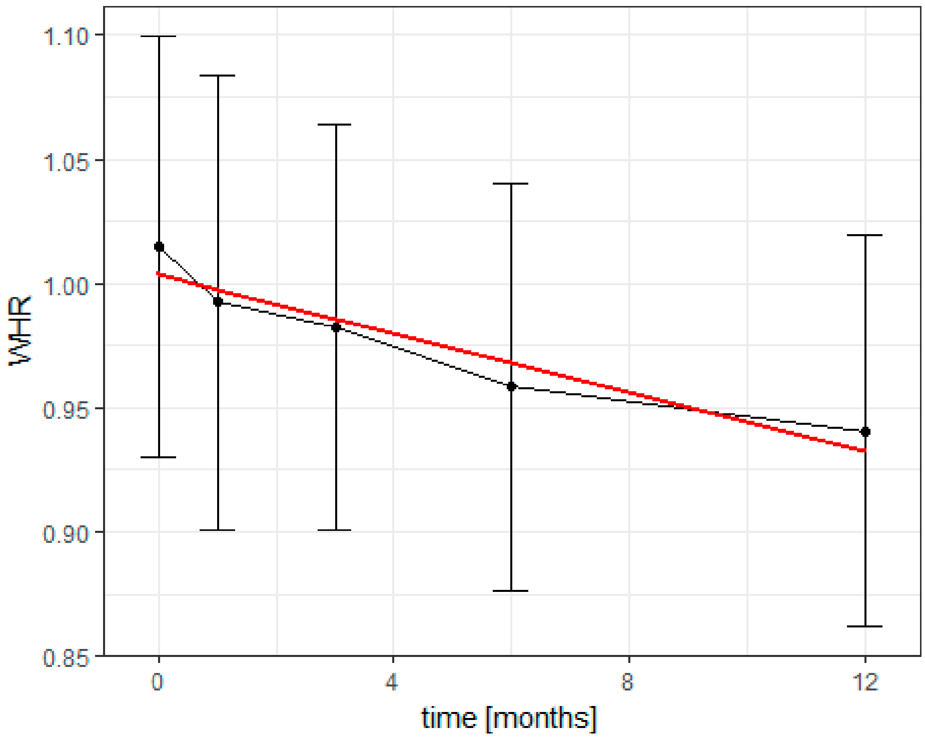

Table 5. Results of linear models for WAIST-TO-HIP RATIO (WHR)

variable	without covariates	model 1	model 2	model 3	model 4	model 5	model 6	model 7
(Intercept)	1***	1***	1***	1***	1***	1***	0.9***	1***
time	-0.006***	-0.006***	-0.006***	-0.006***	-0.006***	-0.005***	-0.006***	-0.005***
IPAQ		-0.000			-0.000	-0.000*		-0.000*
sexM			0.103***		0.103***		0.101***	0.101***
diet_kcal				0.000***		0.000**	0.000**	0.000**
no. of observations	596	576	596	558	576	540	558	540
REML	-1648.6	-1582.4	-1729.8	-1529.4	-1662.9	-1449.5	-1606	-1524.7

Figure 6. Distribution of WAIST-TO-HIP RATIO (WHR) in time - quadratic fit

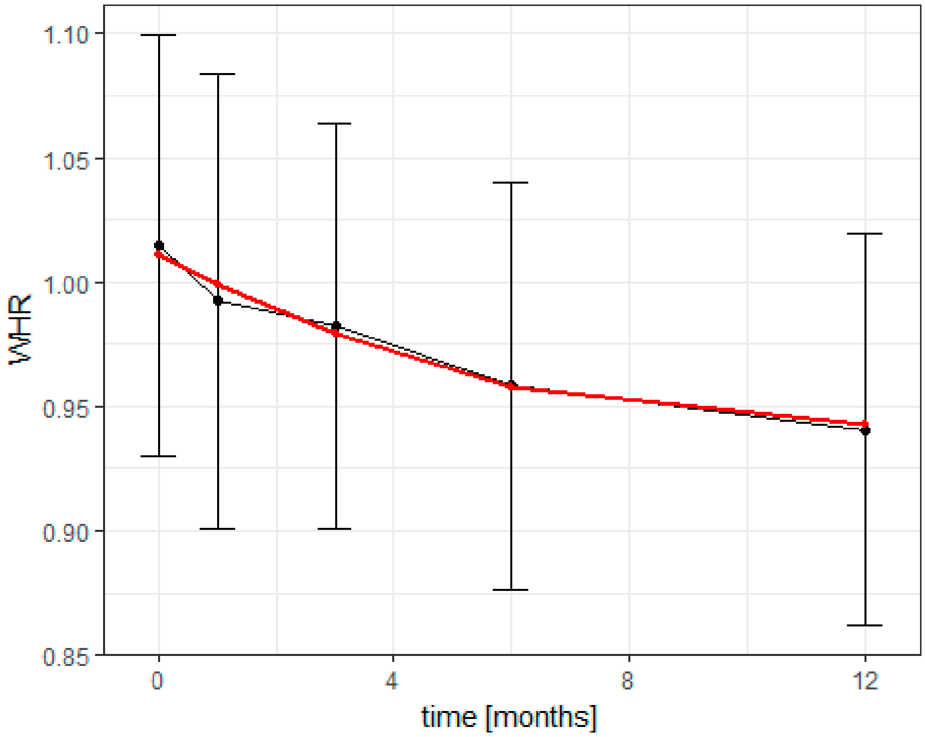

Table 6. Results of quadratic models for WAIST-TO-HIP RATIO (WHR)

variable	without covariates	model 1	model 2	model 3	model 4	model 5	model 6	model 7
(Intercept)	1***	1***	1***	1***	1***	1***	1***	1***
time_sq	0.001***	0.001***	0.001***	0.000*	0.001***	0.000*	0.000**	0.000**
time	-0.012***	- 0.012***	- 0.012***	- 0.010***	- 0.012***	- 0.009***	- 0.010***	- 0.010***
IPAQ		-0.000			-0.000	-0.000*		-0.000*
sexM			0.103***		0.103***		0.102***	0.102***
diet_kcal				0.000*		0.000*	0.000	0.000
no. of observations	596	576	596	558	576	540	558	540
REML	-1649.1	-1584.3	-1730.6	-1520	-1665.2	-1439.1	-1598	-1516

Total body mass (DXA)

Figure 7. Distribution of Total body mass (DXA) in time - linear fit

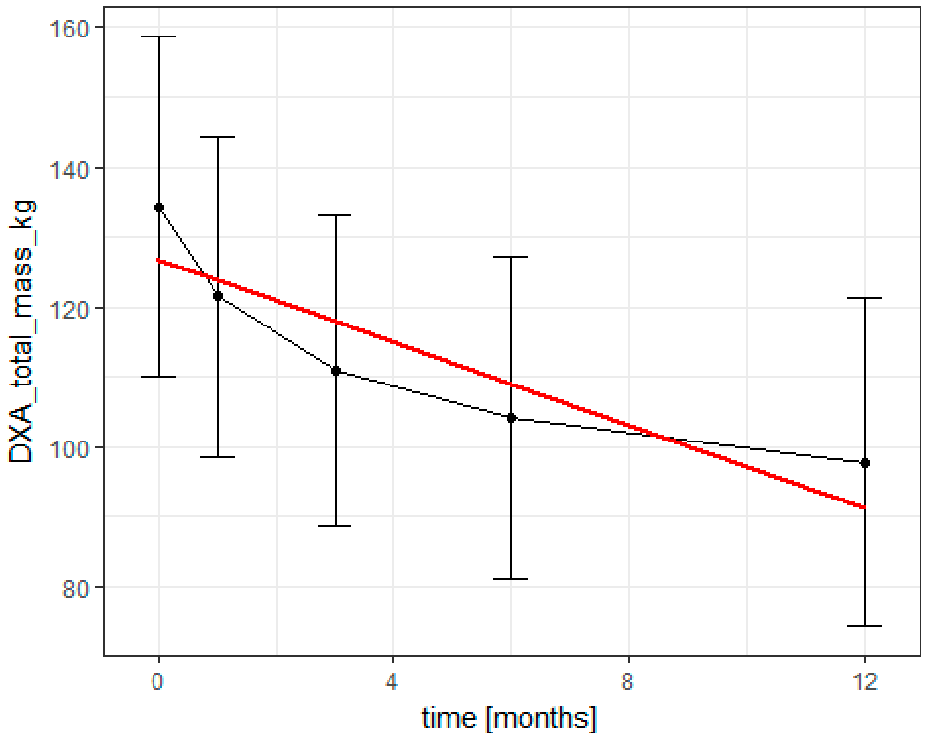

Table 7. Results of linear models for Total body mass (DXA)

variable	without covariates	model 1	model 2	model 3	model 4	model 5	model 6	model 7
(Intercept)	126.8***	126.9***	115.5***	118.6***	115.5***	118.5***	108.4***	108.4***
time	-2.953***	- 2.942***	-2.953***	- 2.770***	-2.938***	- 2.728***	-2.773***	-2.730***
IPAQ		-0.000			-0.000	-0.000		-0.000
sexM			24.565***		24.761***		22.346***	22.337***
diet_kcal				0.008***		0.008***	0.007***	0.008***
no. of observations	594	576	594	556	576	540	556	540
REML	4796.2	4683.4	4745	4368.6	4631.3	4267.5	4325.4	4223.8

Figure 8. Distribution of Total body mass (DXA) in time - quadratic fit

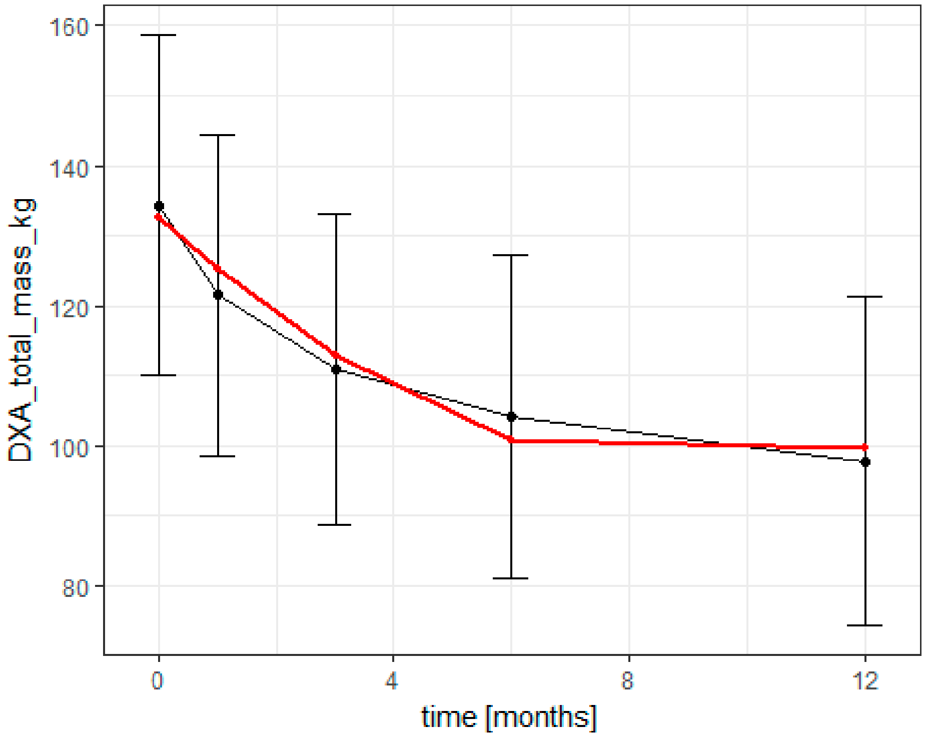

Table 8. Results of quadratic models for Total body mass (DXA)

variable	without covariates	model 1	model 2	model 3	model 4	model 5	model 6	model 7
(Intercept)	132.7***	132.5***	121.3***	126.9***	121.1***	126.7***	116.3***	116.1***
time_sq	0.423***	0.423***	0.423***	0.341***	0.422***	0.334***	0.343***	0.336***
time	-7.830***	- 7.842***	-7.827***	- 6.790***	-7.833***	- 6.686***	-6.814***	-6.708***
IPAQ		0.000			0.000	-0.000		-0.000
sexM			24.747***		24.834***		23.366***	23.389***
diet_kcal				0.004***		0.004***	0.004***	0.004***
no. of observations	594	576	594	556	576	540	556	540
REML	4492.8	4395.4	4442.1	4176.2	4344.5	4091.9	4130.1	4045.6

Fat mass (DXA)

Figure 9. Distribution of Fat mass (DXA) in time - linear fit

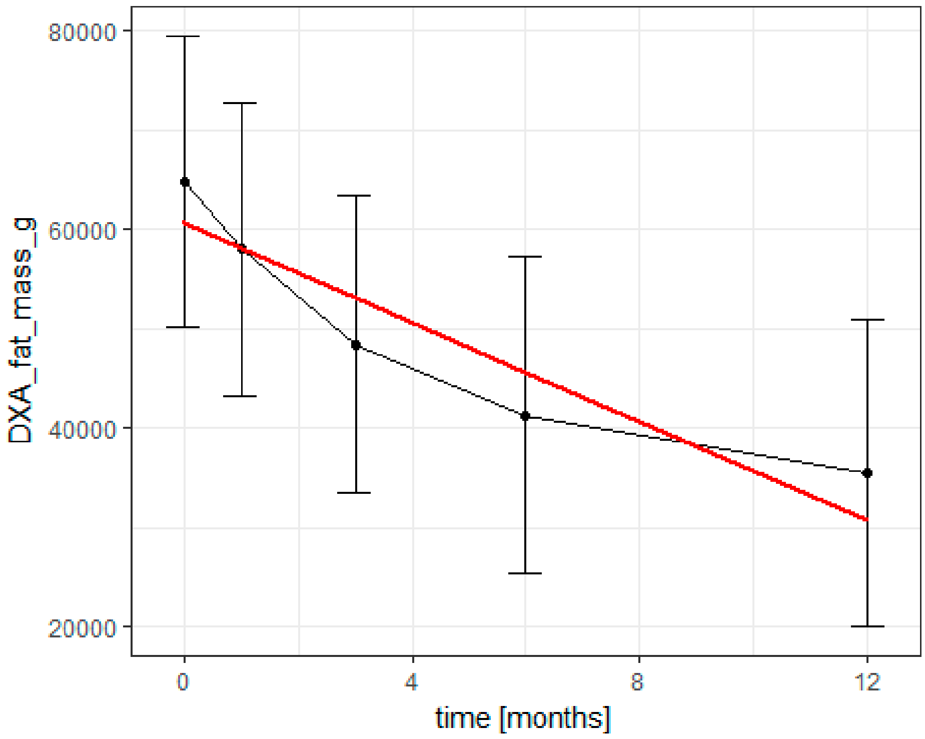

Table 9. Results of linear models for Fat mass (DXA)

variable	without covariates	model 1	model 2	model 3	model 4	model 5	model 6	model 7
(Intercept)	60615.9***	60981.8***	59184.9***	56160.8***	59500.2***	56221.7***	55355.1***	55439.3***
time	-2486.723***	-2451.535***	-2487.064***	-2381.993***	-2451.573***	-2329.766***	-2382.396***	-2330.278***
IPAQ		-0.053			-0.053	-0.063		-0.063
sexM			3116.978		3244.651		1790.427	1749.025
diet_kcal				4.085***		4.412***	4.069***	4.394***
no. of observations	591	573	591	554	573	538	554	538
REML	12575.7	12202	12556.6	11746.9	12182.8	11406.9	11728.9	11388.9

Figure 10. Distribution of Fat mass (DXA) in time - quadratic fit

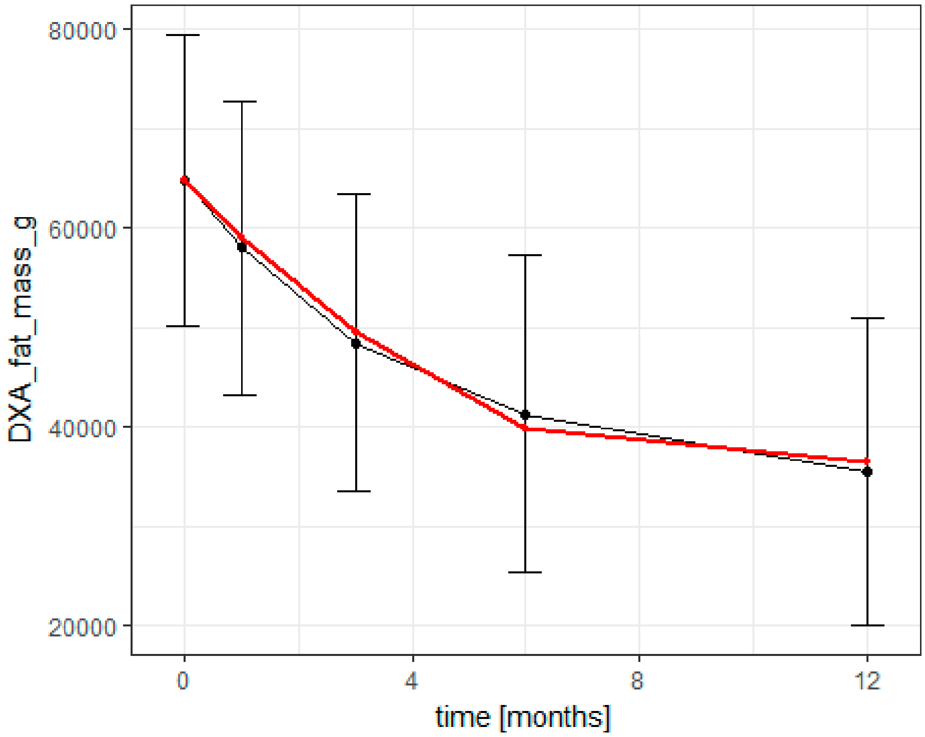

Table 10. Results of quadratic models for Fat mass (DXA)

variable	without covariates	model 1	model 2	model 3	model 4	model 5	model 6	model 7
(Intercept)	64749.4***	64901.4***	63272.3***	62708.6***	63414***	62487.1***	61549.8***	61358.1***
time_sq	298.063***	295.207***	298.097***	269.426***	295.179***	258.542***	269.910***	259.062***
time	-5923.126***	-5874.118***	-5923.736***	-5555.696***	-5873.817***	-5394.596***	-5561.823***	-5401.256***
IPAQ		-0.029			-0.029	-0.040		-0.040
sexM			3216.420		3247.883		2584.916	2533.155
diet_kcal				1.516**		1.838***	1.495**	1.814***
no. of observations	591	573	591	554	573	538	554	538
REML	12343.3	11984.3	12324.2	11588.2	11965.1	11265.3	11569.6	11246.7

Lean mass (DXA)

Figure 11. Distribution of Lean mass (DXA) in time - linear fit

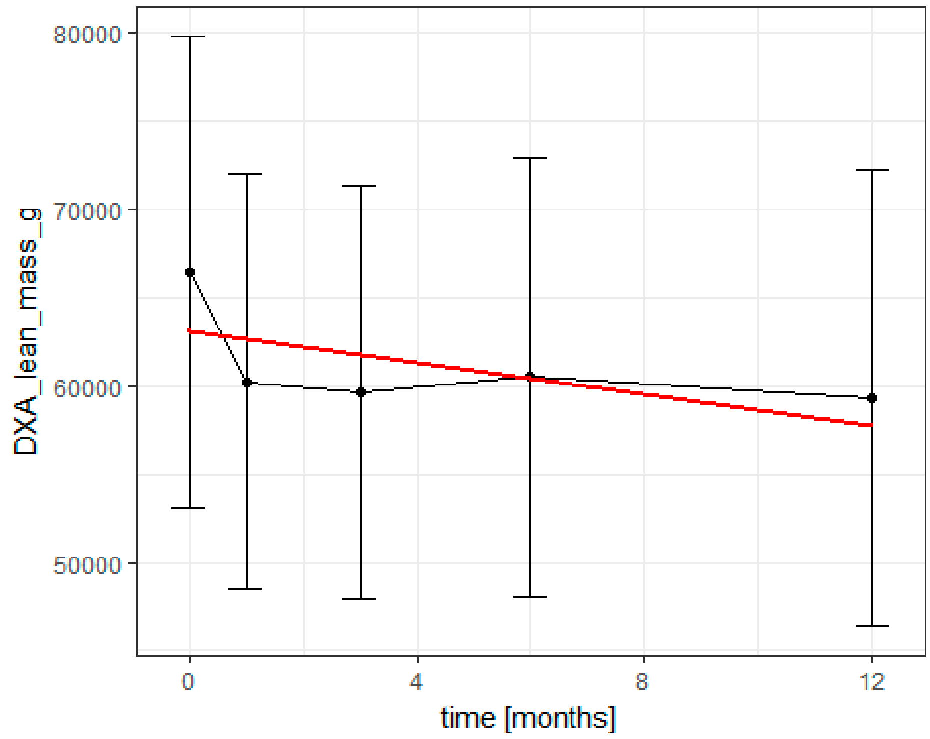

Table 11. Results of linear models for Lean mass (DXA)

variable	without covariates	model 1	model 2	model 3	model 4	model 5	model 6	model 7
(Intercept)	63113.4***	62992.8***	53741.9***	59324***	53568.6***	59362.9***	50442.8***	50457.7***
time	-446.114***	-461.754***	-444.111***	-364.074***	-459.225***	-364.021***	-364.347***	-364.427***
IPAQ		0.024			0.024	0.003		0.004
sexM			20366.844***		20457.442***		19412.243***	19464.163***
diet_kcal				3.499***		3.452***	3.457***	3.402***
no. of observations	593	575	593	555	575	539	555	539
REML	11881.5	11531.1	11704.2	10866.7	11353.8	10569.4	10696.3	10399.4

Figure 12. Distribution of Lean mass (DXA) in time - quadratic fit

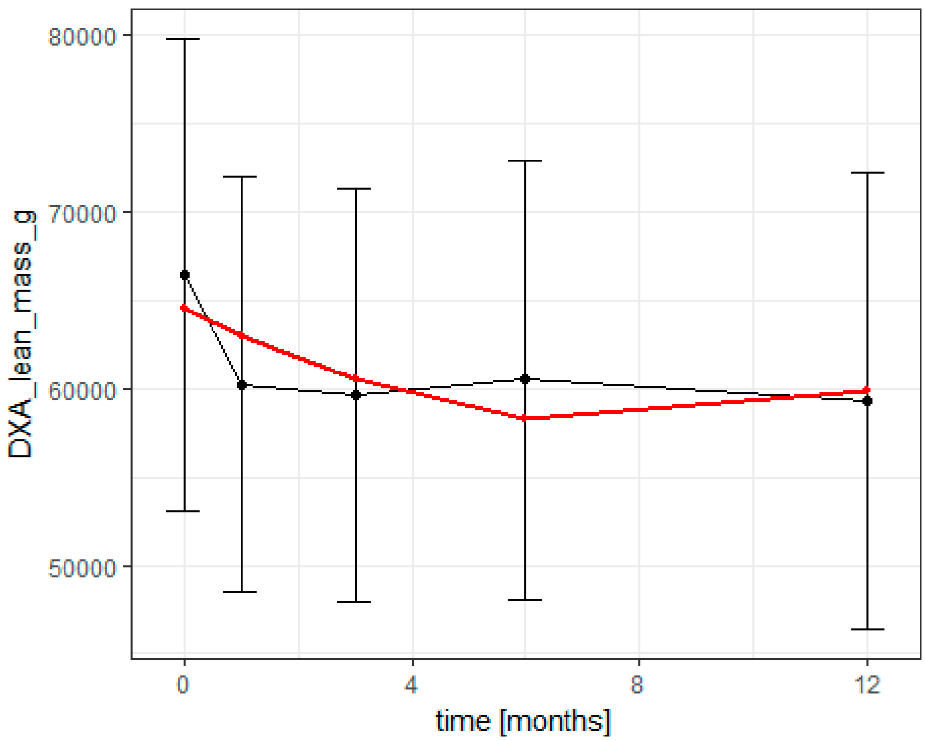

Table 12. Results of quadratic models for Lean mass (DXA)

variable	without covariates	model 1	model 2	model 3	model 4	model 5	model 6	model 7
(Intercept)	64605.6***	64449.9***	55208.6***	60500.6***	55017***	60566.7***	51558.7***	51596.4***
time_sq	108.048***	109.243***	107.656***	48.080***	108.648***	49.092***	48.556***	49.584***
time	-1691.303***	-1726.566***	-1685.569***	-930.634***	-1717.722***	-945.762***	-936.511***	-951.875***
IPAQ		0.031			0.030	0.007		0.007
sexM			20414.718***		20476.583***		19558.795***	19620.486***
diet_kcal				3.031***		2.953***	2.989***	2.904***
no. of observations	593	575	593	555	575	539	555	539
REML	11754.3	11402.3	11577.8	10829.1	11226.4	10531.3	10657.9	10360.6

Visceral adipose tissue mass (DXA)

Figure 13. Distribution of Visceral adipose tissue mass (DXA) in time - linear fit

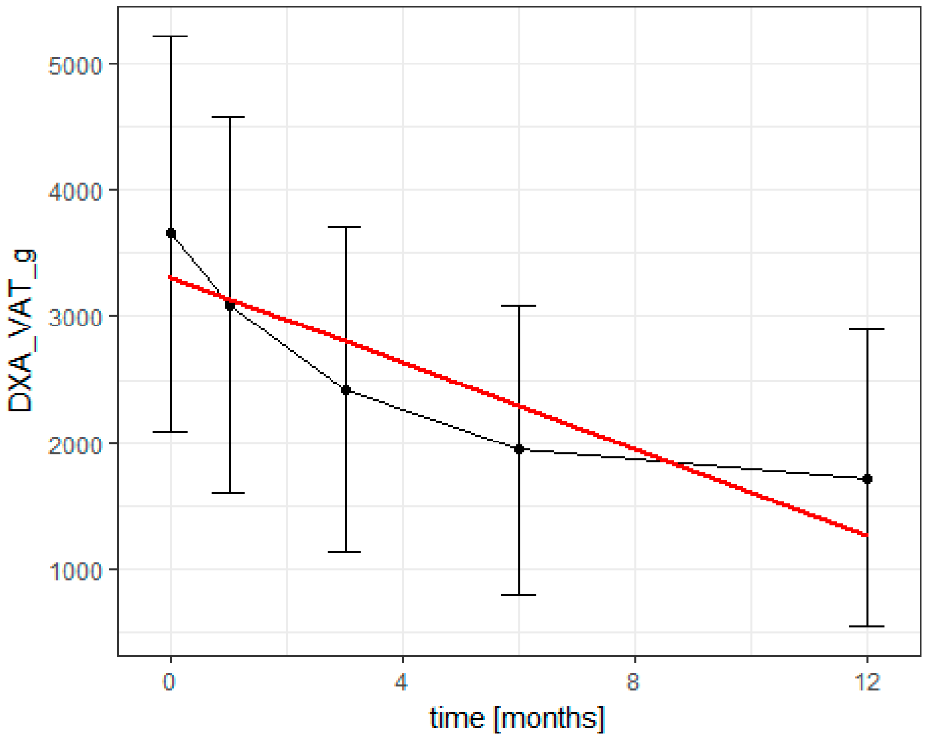

Table 13. Results of linear models for Visceral adipose tissue mass (DXA)

variable	without covariates	model 1	model 2	model 3	model 4	model 5	model 6	model 7
(Intercept)	3305.9***	3327***	2646***	2954.9***	2662.6***	2966.9***	2350.5***	2363.3***
time	-169.413***	-165.922***	-169.355***	-163.757***	-165.457***	-158.022***	-163.898***	-157.939***
IPAQ		-0.003			-0.004	-0.007		-0.007*
sexM			1488.624***		1509.009***		1393.073***	1393.517***
diet_kcal				0.347***		0.386***	0.335***	0.373***
no. of observations	522	506	522	492	506	478	492	478
REML	8576.1	8290.3	8510	8049.5	8221.7	7780	7988.5	7717.6

Figure 14. Distribution of Visceral adipose tissue mass (DXA) in time - quadratic fit

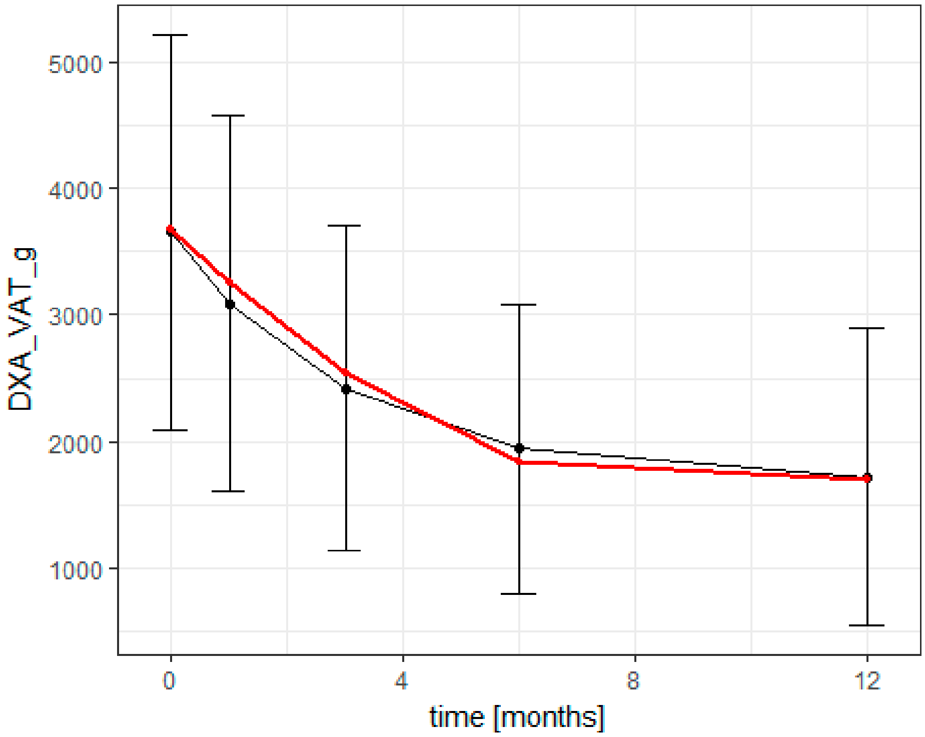

Table 14. Results of quadratic models for Visceral adipose tissue mass (DXA)

variable	without covariates	model 1	model 2	model 3	model 4	model 5	model 6	model 7
(Intercept)	3674.6***	3677.8***	3008.2***	3495.9***	3008.6***	3470.4***	2865.9***	2841.2***
time_sq	23.475***	22.801***	23.556***	21.405***	22.855***	19.742***	21.669***	19.977***
time	-446.199***	-436.265***	-447.151***	-419.184***	-436.575***	-394.915***	-422.439***	-397.642***
IPAQ		-0.002			-0.003	-0.006		-0.006*
sexM			1506.722***		1517.417***		1457.454***	1453.446***
diet_kcal				0.141**		0.187***	0.130**	0.176***
no. of observations	522	506	522	492	506	478	492	478
REML	8416.8	8131	8349	7935.2	8061.2	7673.2	7870.6	7607.6

Total body mass (bioimpedence)

Figure 15. Distribution of Total body mass (bioimpedence) in time - linear fit

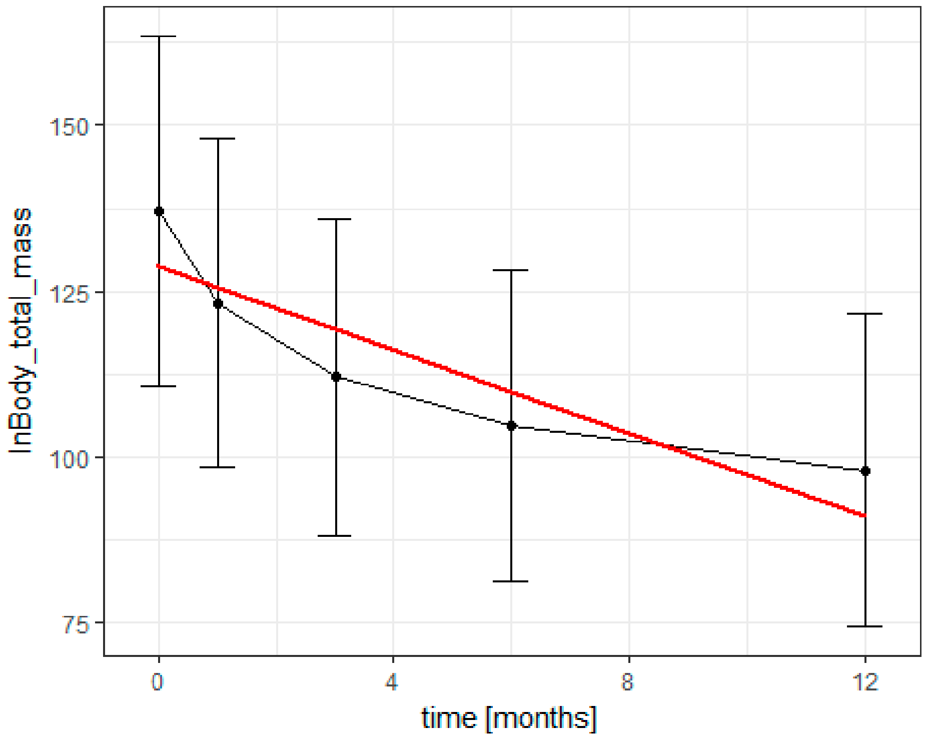

Table 15. Results of linear models for Total body mass (bioimpedence)

variable	without covariates	model 1	model 2	model 3	model 4	model 5	model 6	model 7
(Intercept)	128.7***	128.7***	117.1***	120.1***	117.1***	119.9***	109.7***	109.6***
time	-3.139***	- 3.115***	-3.140***	- 2.930***	-3.111***	- 2.873***	-2.934***	-2.875***
IPAQ		-0.000			-0.000	-0.000		-0.000
sexM			25.471***		25.484***		23.264***	23.002***
diet_kcal				0.008***		0.008***	0.008***	0.008***
no. of observations	601	582	601	562	582	545	562	545
REML	4888	4763	4838.3	4453.7	4711.9	4337.6	4411.2	4294.4

Figure 16. Distribution of Total body mass (bioimpedence) in time - quadratic fit

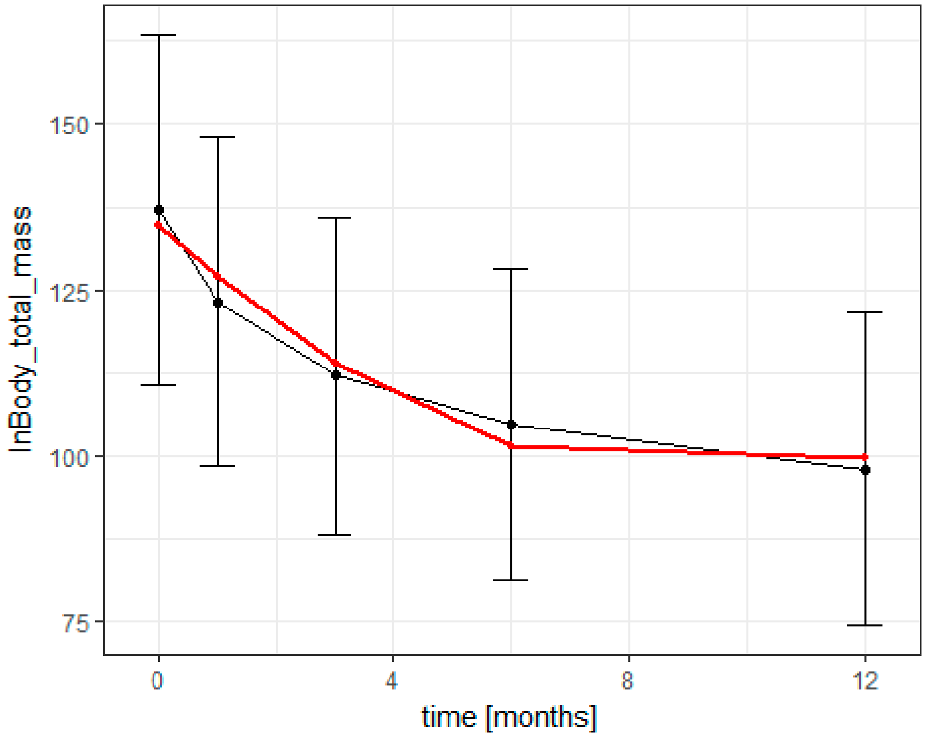

Table 16. Results of quadratic models for Total body mass (bioimpedence)

variable	without covariates	model 1	model 2	model 3	model 4	model 5	model 6	model 7
(Intercept)	134.7***	134.5***	123***	128.9***	122.9***	128.5***	118***	117.7***
time_sq	0.441***	0.439***	0.441***	0.360***	0.438***	0.350***	0.362***	0.352***
time	-8.205***	- 8.184***	-8.204***	- 7.163***	-8.176***	- 7.015***	-7.185***	-7.035***
IPAQ		0.000			0.000	-0.000		-0.000
sexM			25.568***		25.500***		24.230***	24.050***
diet_kcal				0.004***		0.005***	0.004***	0.004***
no. of observations	601	582	601	562	582	545	562	545
REML	4562.6	4458.6	4513.1	4244.5	4408.5	4149.3	4199.3	4103.6

Muscle mass (bioimpedence)

Figure 17. Distribution of Muscle mass (bioimpedence) in time - linear fit

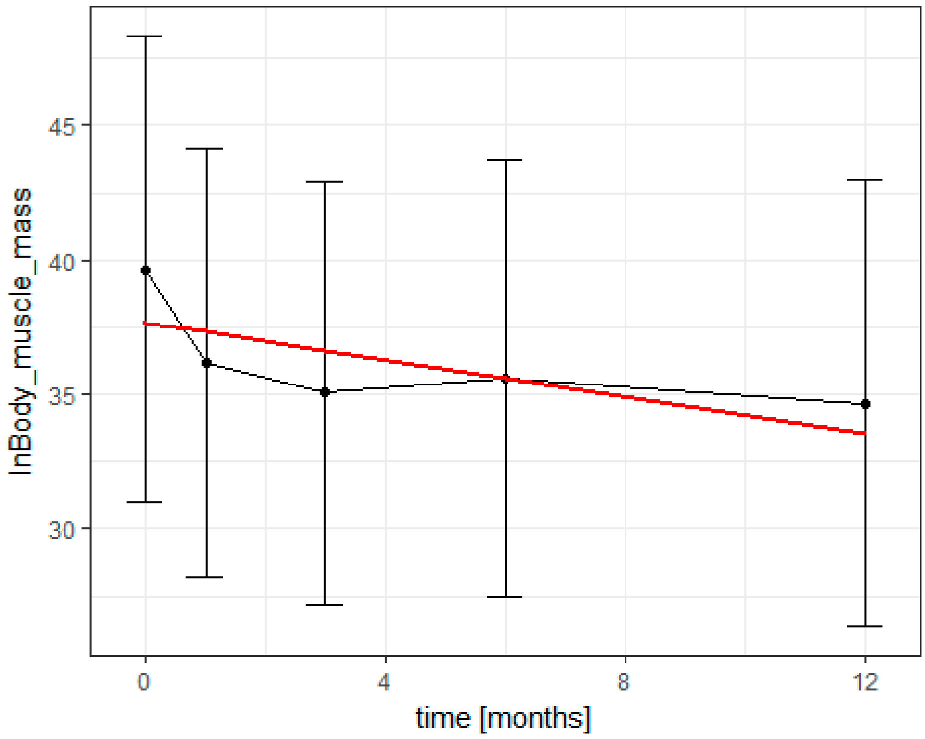

Table 17. Results of linear models for Muscle mass (bioimpedence)

variable	without covariates	model 1	model 2	model 3	model 4	model 5	model 6	model 7
(Intercept)	37.7***	37.5***	31.5***	35.5***	31.3***	35.5***	29.6***	29.5***
time	-0.341***	-0.351***	-0.340***	-0.294***	-0.351***	-0.298***	-0.294***	-0.299***
IPAQ		0.000			0.000*	0.000		0.000
sexM			13.492***		13.586***		12.977***	13.033***
diet_kcal				0.002***		0.002***	0.002***	0.002***
no. of observations	601	582	601	562	582	545	562	545
REML	3160.1	3084.7	2995.7	2771.7	2918.4	2714.5	2614.2	2555.8

Figure 18. Distribution of Muscle mass (bioimpedence) in time - quadratic fit

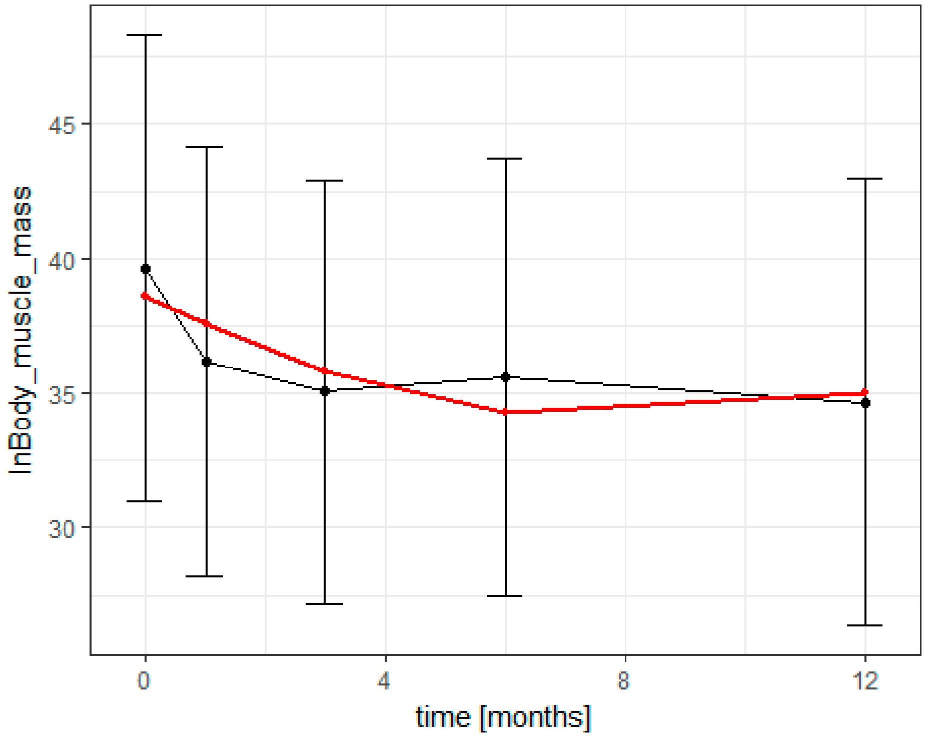

Table 18. Results of quadratic models for Muscle mass (bioimpedence)

variable	without covariates	model 1	model 2	model 3	model 4	model 5	model 6	model 7
(Intercept)	38.6***	38.5***	32.5***	36.5***	32.3***	36.5***	30.6***	30.5***
time_sq	0.071***	0.072***	0.071***	0.040***	0.071***	0.041***	0.040***	0.041***
time	-1.155***	- 1.180***	-1.153***	- 0.760***	-1.176***	- 0.779***	-0.763***	-0.782***
IPAQ		0.000**			0.000**	0.000		0.000
sexM			13.504***		13.582***		13.083***	13.153***
diet_kcal				0.002***		0.002***	0.002***	0.002***
no. of observations	601	582	601	562	582	545	562	545
REML	3009.1	2929.7	2845.4	2722.9	2765.1	2663.4	2564.4	2503.8

Fat mass (bioimpedence)

Figure 19. Distribution of Fat mass (bioimpedence) in time - linear fit

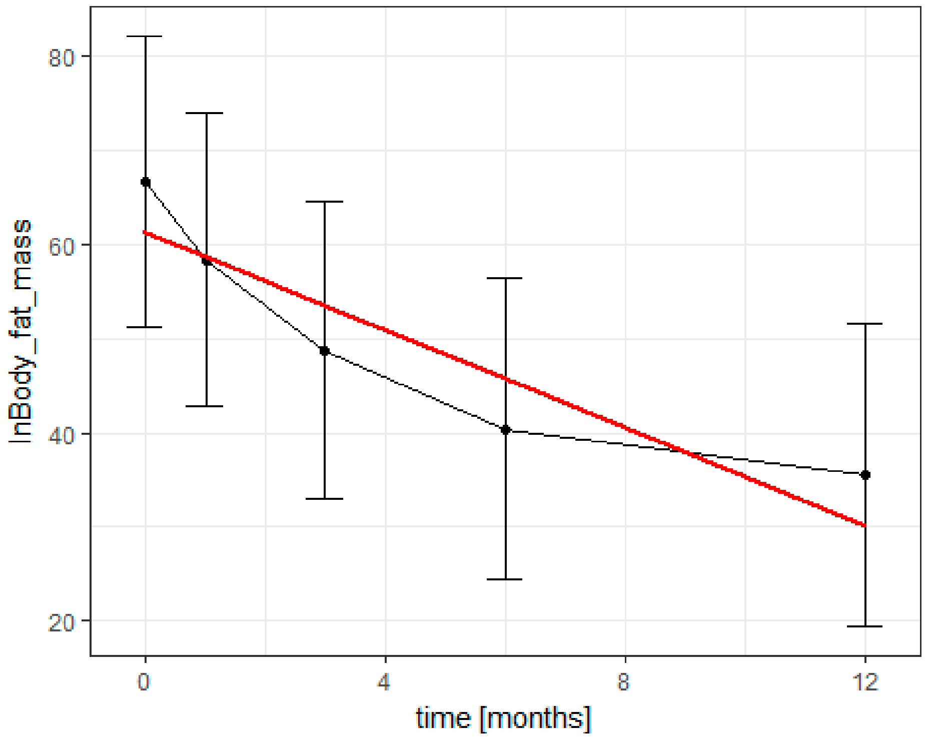

Table 19. Results of linear models for Fat mass (bioimpedence)

variable	without covariates	model 1	model 2	model 3	model 4	model 5	model 6	model 7
(Intercept)	61.3***	61.6***	60***	56.2***	60.4***	56.3***	55.6***	55.8***
time	-2.604***	- 2.556***	- 2.604***	- 2.470***	- 2.556***	- 2.399***	- 2.470***	- 2.399***
IPAQ		-0.000			-0.000	-0.000*		-0.000*
sexM			2.828		2.685		1.426	1.064
diet_kcal				0.005***		0.005***	0.005***	0.005***
no. of observations	601	582	601	562	582	545	562	545
REML	4509.9	4382.5	4505	4167.5	4377.7	4042.6	4163.5	4038.8

Figure 20. Distribution of Fat mass (bioimpedence) in time - quadratic fit

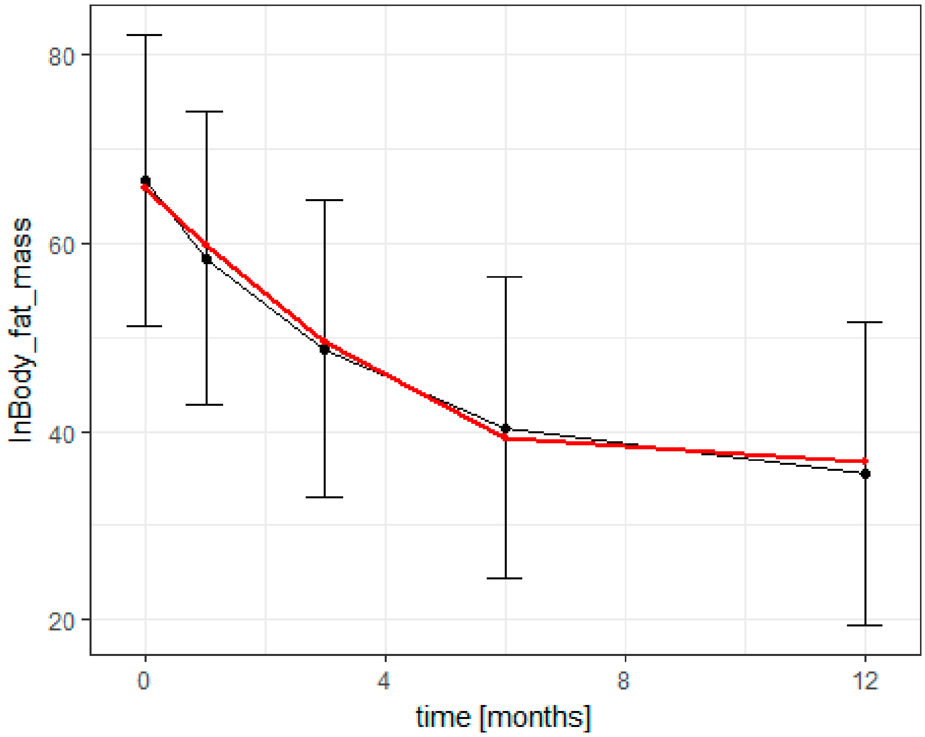

Table 20. Results of quadratic models for Fat mass (bioimpedence)

variable	without covariates	model 1	model 2	model 3	model 4	model 5	model 6	model 7
(Intercept)	65.9***	65.9***	64.5***	63.6***	64.7***	63.4***	62.6***	62.5***
time_sq	0.334***	0.330***	0.334***	0.305***	0.330***	0.293***	0.306***	0.293***
time	-6.436***	- 6.366***	- 6.436***	- 6.056***	- 6.366***	- 5.868***	- 6.060***	- 5.872***
IPAQ		-0.000			-0.000	-0.000		-0.000
sexM			2.909		2.697		2.251	1.941
diet_kcal				0.002***		0.002***	0.002***	0.002***
no. of observations	601	582	601	562	582	545	562	545
REML	4190.8	4083.1	4185.8	3939.2	4078.3	3836.8	3934.7	3832.5

BMI

Figure 21. Distribution of BMI in time - linear fit

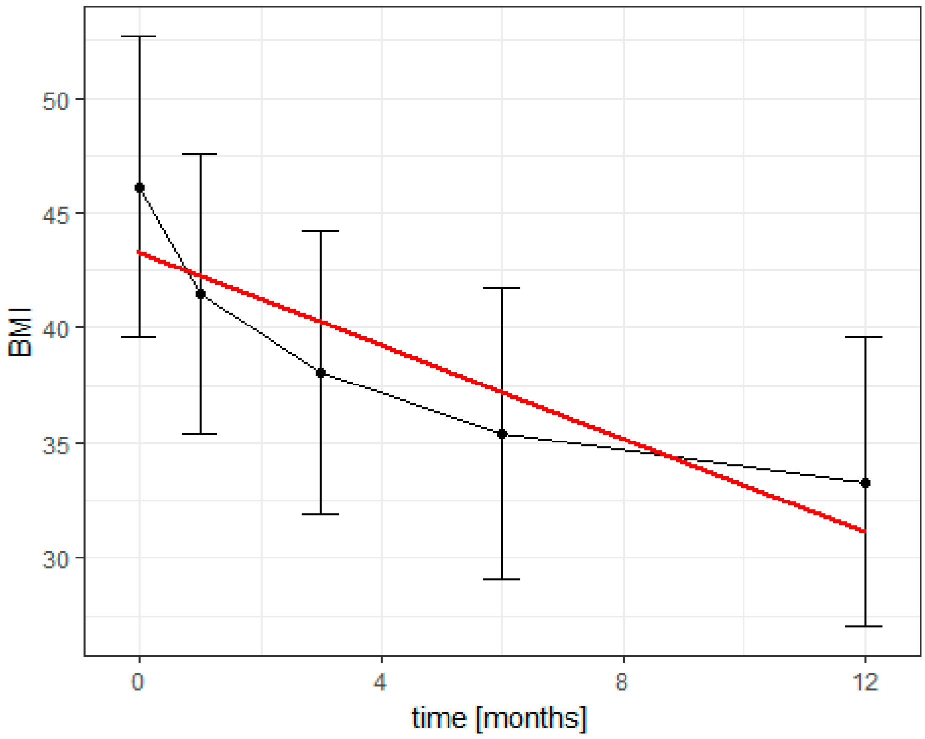

Table 21. Results of linear models for BMI

variable	without covariates	model 1	model 2	model 3	model 4	model 5	model 6	model 7
(Intercept)	43.3***	43.4***	42.7***	40.6***	42.7***	40.6***	40.3***	40.3***
time	-1.017***	- 1.008***	- 1.017***	- 0.955***	- 1.008***	- 0.938***	- 0.955***	- 0.938***
IPAQ		-0.000			-0.000	-0.000		-0.000
sexM			1.402		1.475		0.688	0.698
diet_kcal				0.003***		0.003***	0.003***	0.003***
no. of observations	601	583	601	562	583	546	562	546
REML	3464.8	3391.5	3461	3116.5	3387.5	3052.3	3114.1	3049.9

Figure 22. Distribution of BMI in time - quadratic fit

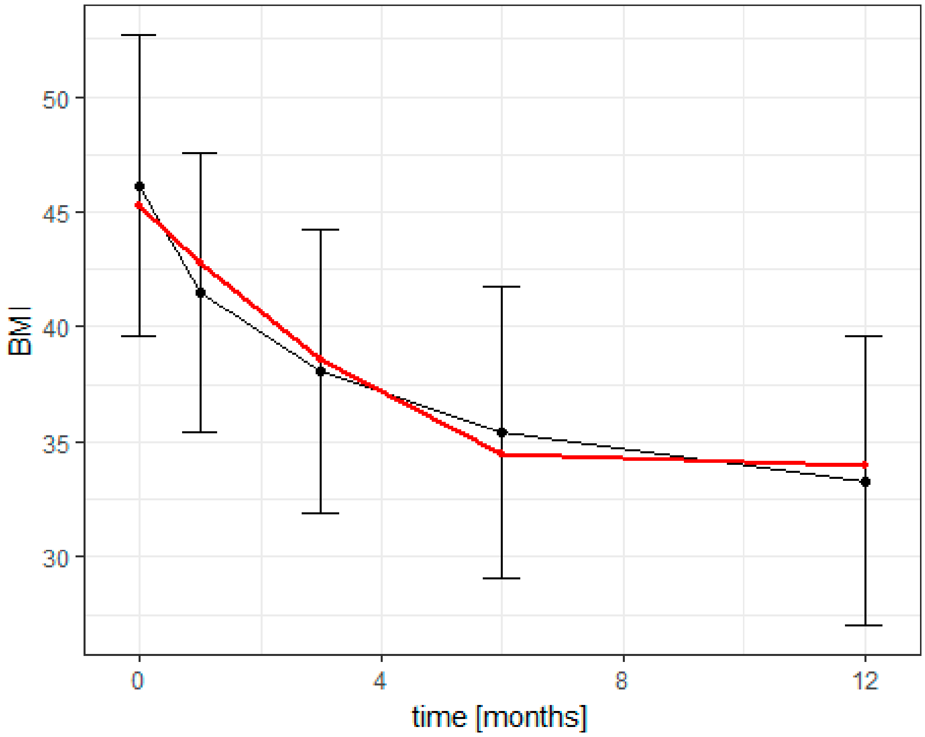

Table 22. Results of quadratic models for BMI

variable	without covariates	model 1	model 2	model 3	model 4	model 5	model 6	model 7
(Intercept)	45.3***	45.2***	44.6***	43.4***	44.6***	43.4***	43***	42.9***
time_sq	0.143***	0.143***	0.143***	0.117***	0.143***	0.115***	0.117***	0.115***
time	-2.665***	-2.667***	-2.666***	-2.332***	-2.667***	-2.302***	-2.334***	-2.304***
IPAQ		0.000			0.000	-0.000		-0.000
sexM			1.447		1.479		1.014	1.036
diet_kcal				0.001***		0.001***	0.001***	0.001***
no. of observations	601	583	601	562	583	546	562	546
REML	3133.3	3076.1	3129.5	2897.3	3072.1	2851	2894.4	2848.1

Supplementary Appendix 2 : Metabolic Generalized Linear Mixed Models

Table 2. Results of linear models for GLUOCSE CONCETRATION AT 0′ (OGTT) ... 29
Table 3. Results of quadratic models for GLUOCSE CONCETRATION AT 0′ (OGTT) ... 30
Table 4. Results of linear models for GLUOCSE CONCETRATION AT 30′ (OGTT) ... 32
Table 5. Results of quadratic models for GLUOCSE CONCETRATION AT 30′ (OGTT) ... 33
Table 6. Results of linear models for GLUOCSE CONCETRATION AT 60′ (OGTT) ... 35
Table 7. Results of quadratic models for GLUOCSE CONCETRATION AT 60′ (OGTT) ... 36
Table 8. Results of linear models for GLUOCSE CONCETRATION AT 120′ (OGTT) ... 38
Table 9. Results of quadratic models for GLUOCSE CONCETRATION AT 120′ (OGTT) ... 39
Table 10. Results of linear models for INSULIN CONCETRATION AT 0′ (OGTT) ... 41
Table 11. Results of quadratic models for INSULIN CONCETRATION AT 0′ (OGTT) ... 42
Table 12. Results of linear models for INSULIN CONCETRATION AT 30′ (OGTT) ... 44
Table 13. Results of quadratic models for INSULIN CONCETRATION AT 30′ (OGTT) ... 45
Table 14. Results of linear models for INSULIN CONCETRATION AT 60′ (OGTT) ... 47
Table 15. Results of quadratic models for INSULIN CONCETRATION AT 60′ (OGTT) ... 48
Table 16. Results of linear models for INSULIN CONCETRATION AT 120′ (OGTT) ... 50
Table 17. Results of quadratic models for INSULIN CONCETRATION AT 120′ (OGTT) ... 51
Table 18. Results of linear models for HbA1c ... 53
Table 19. Results of quadratic models for HbA1c ... 54
Table 20. Results of linear models for HOMA-beta ... 56
Table 21. Results of quadratic models for HOMA-beta ... 57
Table 22. Results of linear models for HOMA-IR ... 59
Table 23. Results of quadratic models for HOMA-IR ... 60
Table 24. Results of linear models for TOTAL CHOLESTEROL ... 62
Table 25. Results of quadratic models for TOTAL CHOLESTEROL ... 63
Table 26. Results of linear models for TRIGLYCERIDES ... 65
Table 27. Results of quadratic models for TRIGLYCERIDES ... 66
Table 28. Results of linear models for HDL CHOLESTEROL ... 68
Table 29. Results of quadratic models for HDL CHOLESTEROL ... 69
Table 30. Results of linear models for LDL CHOLESTEROL ... 71
Table 31. Results of quadratic models for LDL CHOLESTEROL ... 72
Table 32. Results of linear models for ASPARTATE TRANSAMINASE ... 74
Table 33. Results of quadratic models for ASPARTATE TRANSAMINASE ... 75
Table 34. Results of linear models for ALANINE TRANSAMINASE ... 77
Table 35. Results of quadratic models for ALANINE TRANSAMINASE ... 78
Table 36. Results of linear models for C REACTIVE PROTEIN ... 80
Table 37. Results of quadratic models for C REACTIVE PROTEIN ... 81
Table 38. Results of linear models for MEAN INSULIN CONCENTRATION (OGTT) ... 83
Table 39. Results of quadratic models for MEAN INSULIN CONCENTRATION (OGTT) ... 84
Table 40. Results of linear models for MEAN GLUCOSE CONCENTRATION (OGTT) ... 86
Table 41. Results of quadratic models for MEAN GLUCOSE CONCENTRATION (OGTT) ... 87
Table 42. Results of linear models for MATSUDA INDEX ... 89
Table 43. Results of quadratic models for MATSUDA INDEX ... 90
Table 44. Results of linear models for GLUCOSE AREA UNDER THE CURVE (OGTT) ... 92
Table 45. Results of quadratic models for GLUCOSE AREA UNDER THE CURVE (OGTT) ... 93
Table 46. Results of linear models for INSULIN AREA UNDER THE CURVE (OGTT) ... 95
Table 47. Results of quadratic models for INSULIN AREA UNDER THE CURVE (OGTT) ... 96

GLUOCSE CONCETRATION AT 0′ (OGTT)

Figure 23. Distribution of GLUOCSE CONCETRATION AT 0′ (OGTT) in time - linear fit

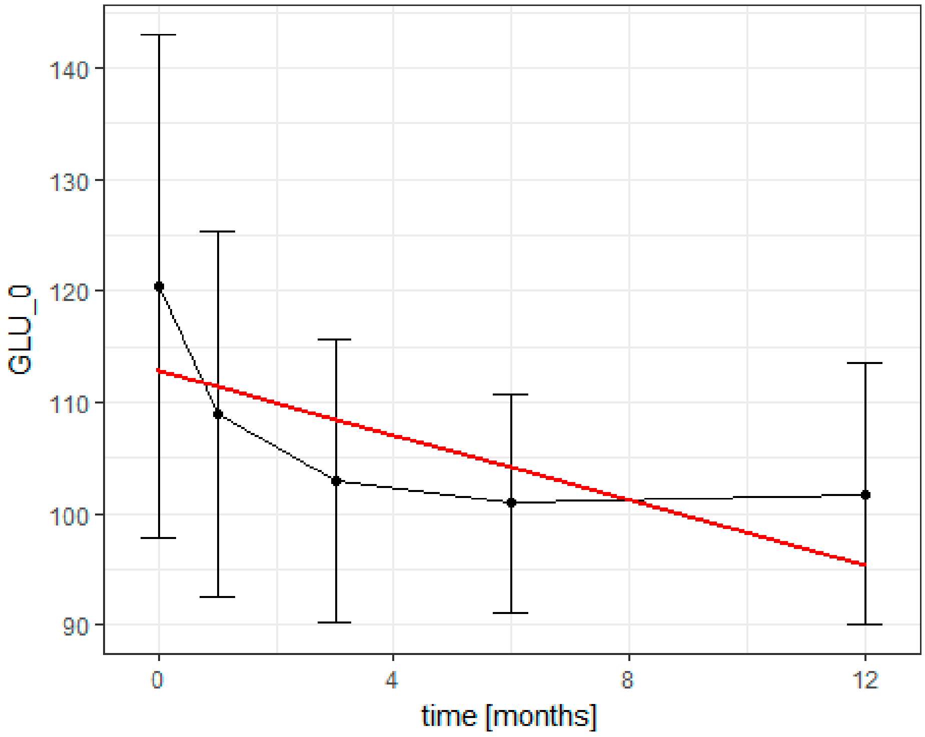

Table 23. Results of linear models for GLUOCSE CONCETRATION AT 0′ (OGTT)

variable	without covariates	model 1	model 2	model 3	model 4	model 5	model 6	model 7	model 8	model 9	model 10	model 11	model 12	model 13	model 14	model 15
(Intercept)	112.8***	77.7***	113.3***	112.6***	105.4***	78.1***	74.8***	84.5***	113.1***	105.7***	106***	75.1***	85.5***	82***	106.3***	82.8***
time	-1.456***	-0.640***	-1.444***	-1.456***	-1.263***	-0.644***	-0.491**	-0.781***	-1.444***	-1.237***	-1.262***	-0.494**	-0.778***	-0.657***	-1.236***	-0.649***
total_mass_kg		0.277***				0.276***	0.326***	0.174***				0.327***	0.170***	0.217***		0.215***
IPAQ			-0.000			-0.000			-0.000	-0.000		-0.000	-0.000		-0.000	-0.000
sexM				0.450			-7.610**		0.443		-1.252	-7.642**		-6.137*	-1.432	-6.273*

diet_kcal					0.007***			0.006***		0.007***	0.007***		0.006***	0.006***	0.007***	0.006***
no. of observations	602	599	581	602	563	581	599	560	581	544	563	581	544	560	544	544
AIC	4927.9	4863.6	4782.8	4924.4	4535.1	4742.4	4851.4	4499	4779.3	4405.5	4531.4	4730	4393.3	4489.1	4401.7	4383.2

Figure 24. Distribution of GLUOCSE CONCETRATION AT 0′ (OGTT) in time - quadratic fit

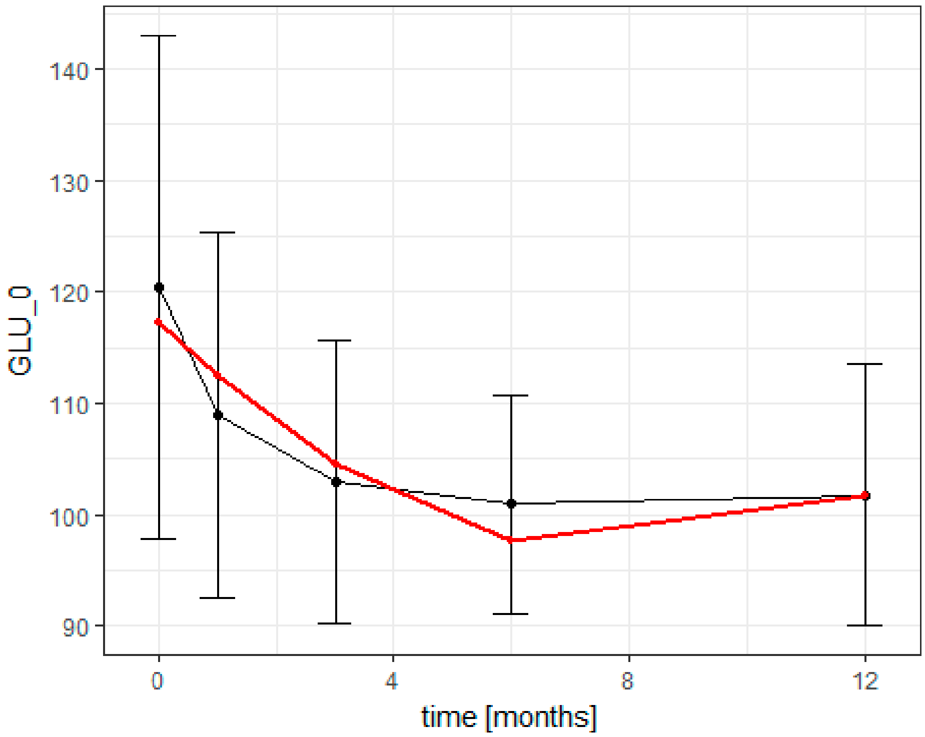

Table 24. Results of quadratic models for GLUOCSE CONCETRATION AT 0′ (OGTT)

variable	without covariates	model 1	model 2	model 3	model 4	model 5	model 6	model 7	model 8	model 9	model 10	model 11	model 12	model 13	model 14	model 15
(Intercept)	117.3***	101***	117.6***	117.1***	111.5***	101.4***	98.8***	100.8***	117.4***	111.8***	111.7***	99.1***	101.5***	98.8***	112.1***	99.3***
time_sq	0.326***	0.275***	0.332***	0.327***	0.255***	0.280***	0.264***	0.227***	0.332***	0.257***	0.255***	0.269***	0.231***	0.217***	0.256***	0.220***
time	-5.221***	-4.263***	-5.283***	-5.222***	-4.268***	-4.332***	-4.049***	-3.709***	-5.284***	-4.275***	-4.263***	-4.113***	-3.751***	-3.511***	-4.269***	-3.540***

total_mass_kg		0.123**				0.123**	0.150**	0.083*				0.151**	0.082	0.108*		0.110*
IPAQ			-0.000			-0.000			-0.000	-0.000		-0.000	-0.000		-0.000	-0.000
sexM				0.495			-3.215		0.398		-0.561	-3.326		-3.085	-0.727	-3.290
diet_kcal					0.004***			0.004***		0.005***	0.004***		0.004***	0.004***	0.005***	0.004***
no. of observations	602	599	581	602	563	581	599	560	581	544	563	581	544	560	544	544
AIC	4839.5	4813.3	4695.9	4836	4488.3	4691.7	4808	4467.3	4692.5	4360.8	4484.8	4686.3	4361.5	4462.1	4357.3	4356

GLUOCSE CONCETRATION AT 30′ (OGTT)

Figure 25. Distribution of GLUOCSE CONCETRATION AT 30′ (OGTT) in time - linear fit

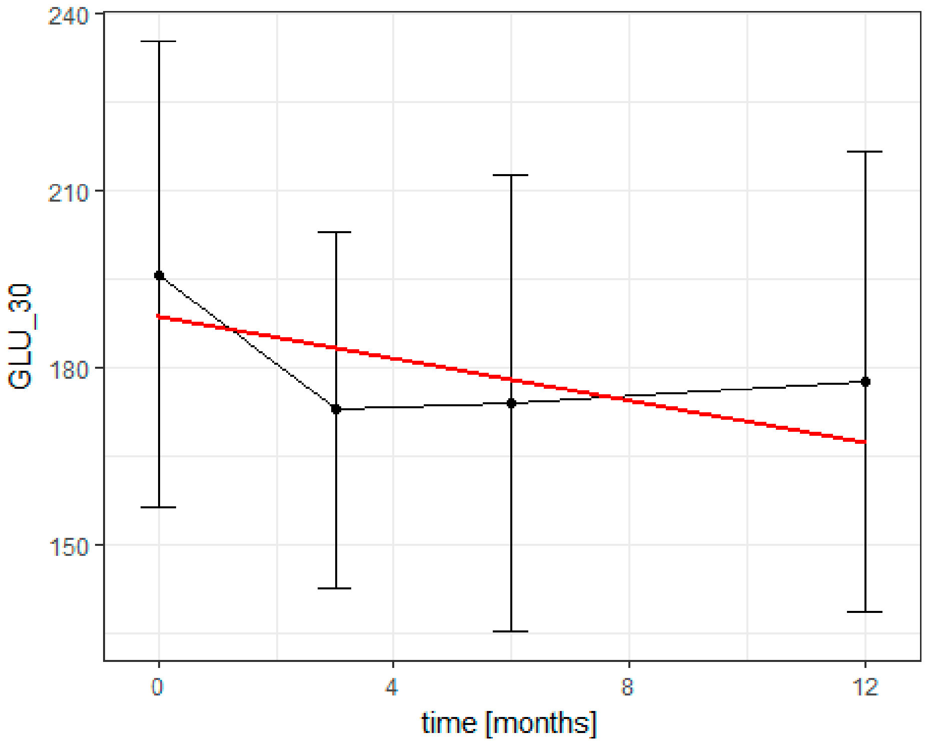

Table 25. Results of linear models for GLUOCSE CONCETRATION AT 30′ (OGTT)

variable	without covariates	model 1	model 2	model 3	model 4	model 5	model 6	model 7	model 8	model 9	model 10	model 11	model 12	model 13	model 14	model 15
(Intercept)	188.6***	162.1***	190.8***	190.3***	169.2***	168***	155.9***	167.2***	192.5***	168.6***	171.7***	162***	173.4***	163***	171.2***	169.3***
time	-1.769***	-1.148*	-1.678***	-1.747***	-0.860*	-1.132*	-0.819	-0.823	-1.657***	-0.718	-0.806*	-0.826	-0.822	-0.600	-0.660	-0.613
total_mass_kg		0.204*				0.174	0.289**	-0.003				0.254*	-0.041	0.059		0.017
IPAQ			-0.000			-0.000			-0.000	-0.000		-0.000	-0.000		-0.000	-0.000
sexM				-3.634			-10.547		-3.526		-6.261	-9.704		-7.418	-6.506	-6.888
diet_kcal					0.014***			0.016***		0.016***	0.014***		0.016***	0.015***	0.016***	0.016***

no. of observations	386	384	378	386	355	378	384	353	378	348	355	378	348	353	348	348
AIC	3810	3791	3749.2	3804.3	3485.3	3749.5	3782.8	3466.1	3743.5	3430.1	3478.8	3741.7	3432.6	3459.2	3424.3	3425.9

Figure 26. Distribution of GLUOCSE CONCETRATION AT 30′ (OGTT) in time - quadratic fit

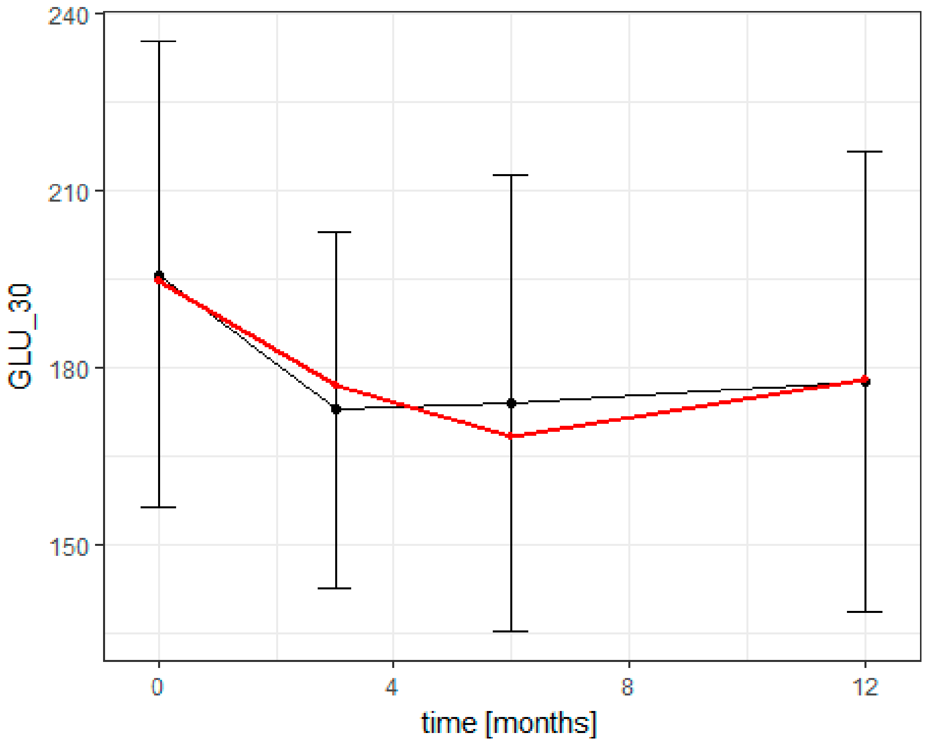

Table 26. Results of quadratic models for GLUOCSE CONCETRATION AT 30′ (OGTT)

variable	without covariates	model 1	model 2	model 3	model 4	model 5	model 6	model 7	model 8	model 9	model 10	model 11	model 12	model 13	model 14	model 15
(Intercept)	194.6***	202.6***	196.8***	195.9***	182***	208.8***	201.1***	190.8***	198.1***	180.8***	183.2***	207.8***	197.3***	188.4***	182.2***	195.2***
time_sq	0.501***	0.523***	0.491***	0.500***	0.351**	0.528***	0.515***	0.344**	0.490***	0.310**	0.341**	0.523***	0.349**	0.330**	0.298**	0.337**
time	-7.395***	-7.851***	-7.198***	-7.369***	-5.208***	-7.898***	-7.700***	-5.332***	-7.172***	-4.561**	-5.050***	-7.805***	-5.380**	-5.065**	-4.374**	-5.158**

total_mass_kg		-0.059				-0.089	-0.042	-0.093				-0.078	-0.130	-0.068		-0.108
IPAQ			-0.000			-0.000			-0.000	-0.000		-0.000	-0.000		-0.000	-0.000
sexM				-2.818			-1.685		-2.905		-4.111	-0.983		-2.576	-4.737	-2.125
diet_kcal					0.008**			0.011**		0.010**	0.009**		0.011***	0.011***	0.011**	0.011***
no. of observations	386	384	378	386	355	378	384	353	378	348	355	378	348	353	348	348
AIC	3780.7	3765.7	3722.4	3775.2	3477.7	3724.4	3760.1	3460	3716.8	3425.3	3471.9	3718.8	3426.5	3454.3	3419.3	3420.9

GLUOCSE CONCETRATION AT 60′ (OGTT)

Figure 27. Distribution of GLUOCSE CONCETRATION AT 60′ (OGTT) in time - linear fit

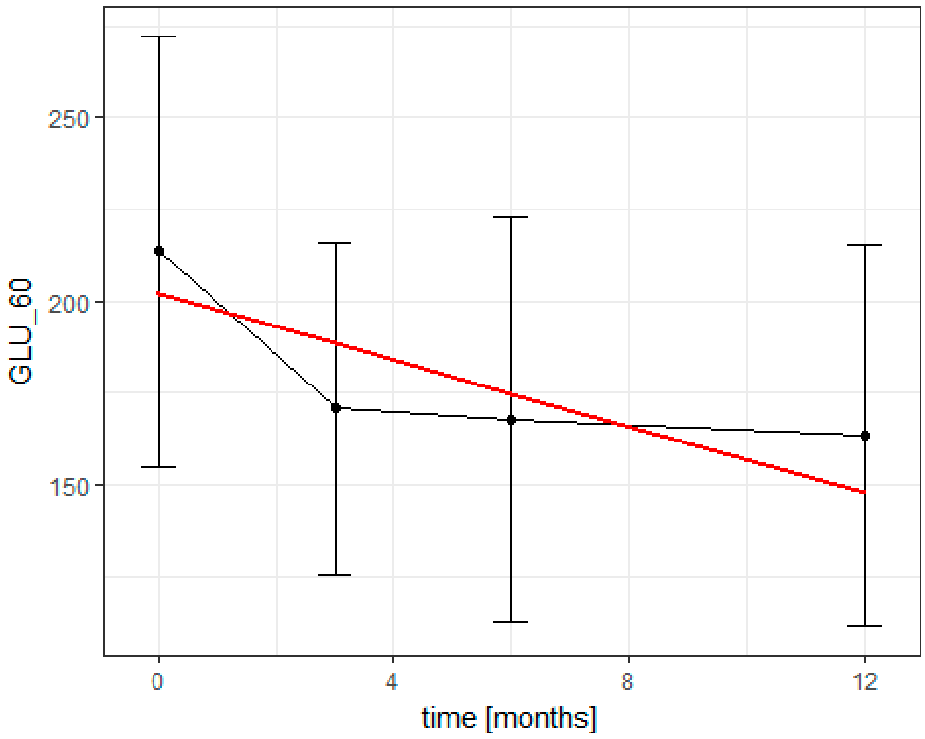

Table 27. Results of linear models for GLUOCSE CONCETRATION AT 60′ (OGTT)

variable	without covariates	model 1	model 2	model 3	model 4	model 5	model 6	model 7	model 8	model 9	model 10	model 11	model 12	model 13	model 14	model 15
(Intercept)	202***	131.1***	205.8***	202.3***	175***	136.5***	123.5***	132.1***	206.2***	176.1***	176.8***	128.1***	137.9***	125.9***	178***	131***
time	-4.500***	-2.828***	-4.445***	-4.496***	-3.249***	-2.765***	-2.368***	-2.347***	-4.441***	-3.134***	-3.210***	-2.294**	-2.288**	-1.982**	-3.093***	-1.898**
total_mass_kg		0.547***				0.529***	0.664***	0.345*				0.651***	0.327*	0.446**		0.437**
IPAQ			-0.000			-0.000			-0.000	-0.000		-0.000	-0.000		-0.000	-0.000
sexM				-0.772			-16.310		-0.853		-4.573	-16.568		-13.575	-4.758	-14.303

diet_kcal					0.019***			0.018***		0.021***	0.019***		0.018***	0.018***	0.021***	0.018***
no. of observations	383	381	376	383	352	376	381	350	376	346	352	376	346	350	346	346
AIC	4058.5	4025.2	3999.1	4052.5	3715.7	3988.8	4015.8	3687.3	3993.1	3661.9	3709.3	3979.3	3659.7	3678.9	3655.6	3651.1

Figure 28. Distribution of GLUOCSE CONCETRATION AT 60' (OGTT) in time - quadratic fit

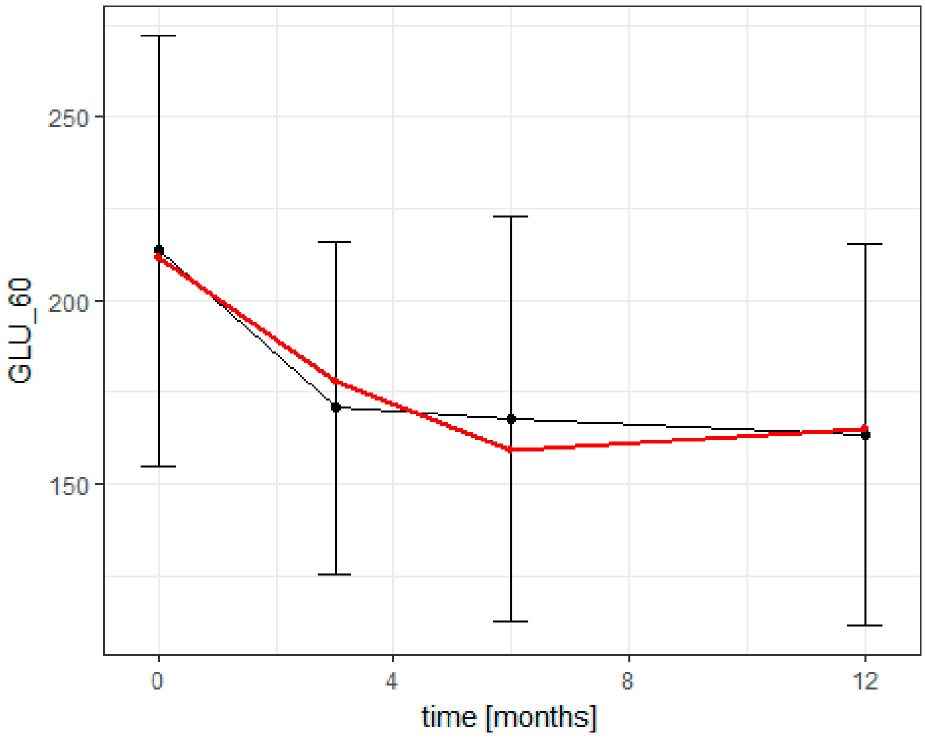

Table 28. Results of quadratic models for GLUOCSE CONCETRATION AT 60' (OGTT)

variable	without covariates	model 1	model 2	model 3	model 4	model 5	model 6	model 7	model 8	model 9	model 10	model 11	model 12	model 13	model 14	model 15
(Intercept)	211.5***	189.5***	215.4***	211.2***	201.2***	197.1***	186.5***	175.7***	215.2***	204.2***	201.1***	194***	186.1***	171.8***	204.3***	181.9***
time_sq	0.806***	0.728***	0.816***	0.807***	0.713***	0.759***	0.712***	0.608***	0.816***	0.717***	0.713***	0.742***	0.673***	0.584***	0.716***	0.649***
time	-13.543***	-12.214***	-13.614***	-13.551***	-12.085***	-12.544***	-11.898***	-10.357***	-13.619***	-12.030***	-12.090***	-12.225***	-11.127***	-9.915***	-12.014***	-10.672***

total_mass_kg		0.164				0.135	0.199	0.170				0.171	0.142	0.212		0.186
IPAQ			-0.000			-0.000			-0.000	-0.000		-0.000	-0.000		-0.000	-0.000
sexM				0.671			-3.829		0.364		0.168	-3.850		-4.724	-0.431	-4.878
diet_kcal					0.008			0.010*		0.009	0.008		0.008	0.010*	0.009	0.008
no. of observations	383	381	376	383	352	376	381	350	376	346	352	376	346	350	346	346
AIC	4019.4	3999.7	3960.4	4013.4	3697.1	3961.6	3993.3	3675.5	3954.4	3644.2	3691	3955.2	3645.3	3668.9	3638.1	3638.7

GLUOCSE CONCETRATION AT 120′ (OGTT)

Figure 29. Distribution of GLUOCSE CONCETRATION AT 120′ (OGTT) in time - linear fit

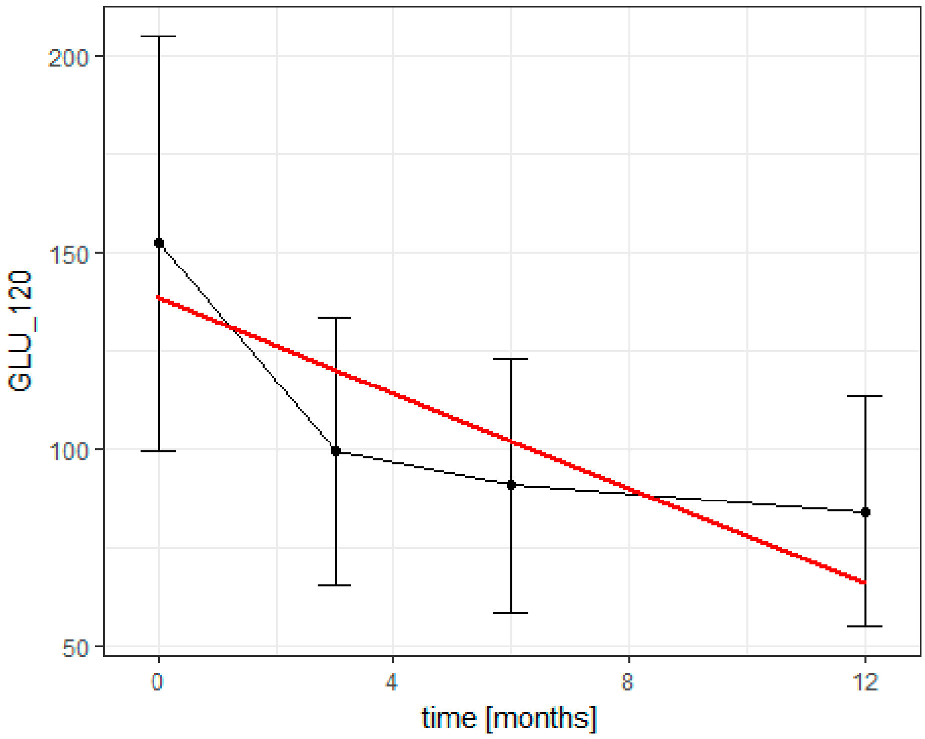

Table 29. Results of linear models for GLUOCSE CONCETRATION AT 120′ (OGTT)

variable	without covariates	model 1	model 2	model 3	model 4	model 5	model 6	model 7	model 8	model 9	model 10	model 11	model 12	model 13	model 14	model 15
(Intercept)	138.3***	76.3***	139.6***	142***	111.6***	78.2***	62.6***	80.3***	143.5***	110.8***	116.1***	63.8***	82.3***	68.6***	115.6***	70***
time	-6.028***	-4.545***	-5.956***	-5.960***	-4.863***	-4.511***	-3.764***	-4.184***	-5.880***	-4.839***	-4.710***	-3.725***	-4.226***	-3.499***	-4.676***	-3.523***
total_mass_kg		0.477***				0.469***	0.668***	0.256*				0.666***	0.242*	0.427***		0.420**
IPAQ			-0.000			-0.000			-0.000	-0.000		-0.000	-0.000		-0.000	-0.000
sexM				-7.928			-24.038***		-8.459		-12.023*	-24.372***		-21.242**	-12.432*	-21.740**

diet_kcal					0.018***			0.017***		0.019***	0.019***		0.017***	0.016***	0.020***	0.017***
no. of observations	384	382	376	384	352	376	382	350	376	346	352	376	346	350	346	346
AIC	3947.4	3914.6	3884.4	3940.2	3590.9	3872.5	3896.8	3569	3876.9	3546.9	3581.3	3854.6	3545.5	3553.2	3537.1	3529.3

Figure 30. Distribution of GLUOCSE CONCETRATION AT 120' (OGTT) in time - quadratic fit

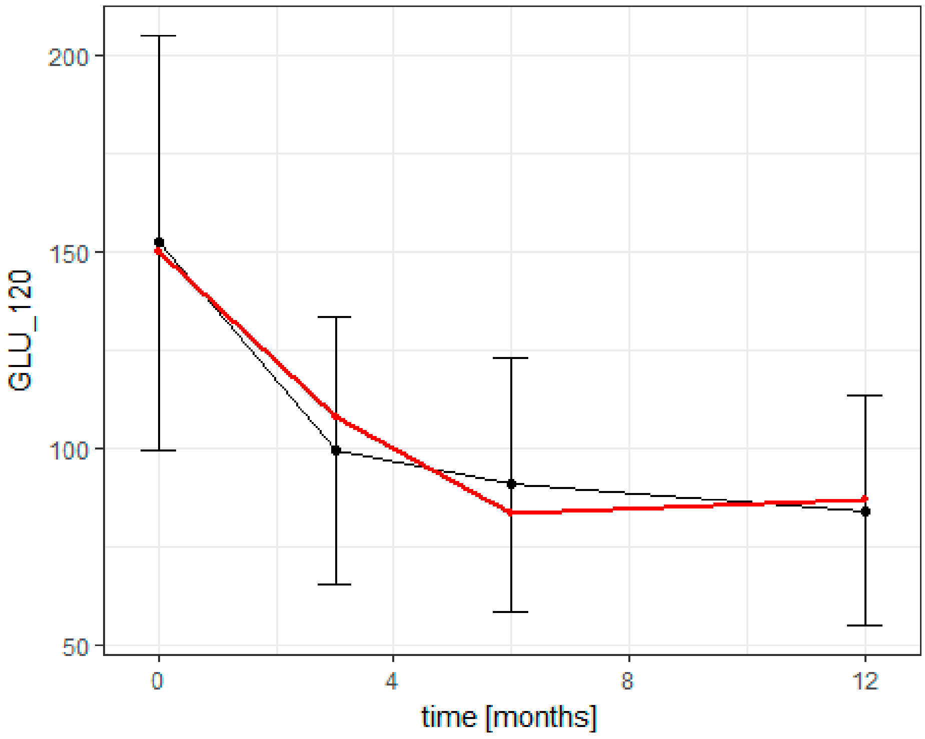

Table 30. Results of quadratic models for GLUOCSE CONCETRATION AT 120' (OGTT)

variable	without covariates	model 1	model 2	model 3	model 4	model 5	model 6	model 7	model 8	model 9	model 10	model 11	model 12	model 13	model 14	model 15
(Intercept)	150.1***	134.9***	151.4***	152.9***	143.1***	138.1***	125.7***	130.8***	154.6***	145.3***	144.8***	128.3***	135.1***	120.5***	147.2***	124.2***
time_sq	0.975***	0.925***	0.982***	0.971***	0.891***	0.941***	0.874***	0.860***	0.978***	0.918***	0.868***	0.887***	0.895***	0.792***	0.894***	0.824***
time	-16.968***	-16.053***	-17.012***	-16.888***	-15.825***	-16.246***	-15.055***	-15.204***	-16.925***	-16.161***	-15.473***	-15.205***	-15.675***	-13.961***	-15.774***	-14.391***

total_mass_kg		0.112				0.098	0.219	0.096				0.211	0.080	0.201		0.190
IPAQ			-0.000			-0.000			-0.000	-0.000		-0.000	-0.000		-0.000	-0.000
sexM				-6.038			-11.585		-6.945		-6.584	-12.132		-11.448	-7.364	-11.935
diet_kcal					0.005			0.004		0.004	0.005		0.004	0.005	0.005	0.005
no. of observations	384	382	376	384	352	376	382	350	376	346	352	376	346	350	346	346
AIC	3872.6	3856	3812.6	3866.1	3551.8	3814.4	3847.4	3535.4	3805.8	3508.3	3545.2	3805.5	3510.3	3526.9	3501.3	3501.5

INSULIN CONCETRATION AT 0′ (OGTT)

Figure 31. Distribution of INSULIN CONCETRATION AT 0′ (OGTT) in time - linear fit

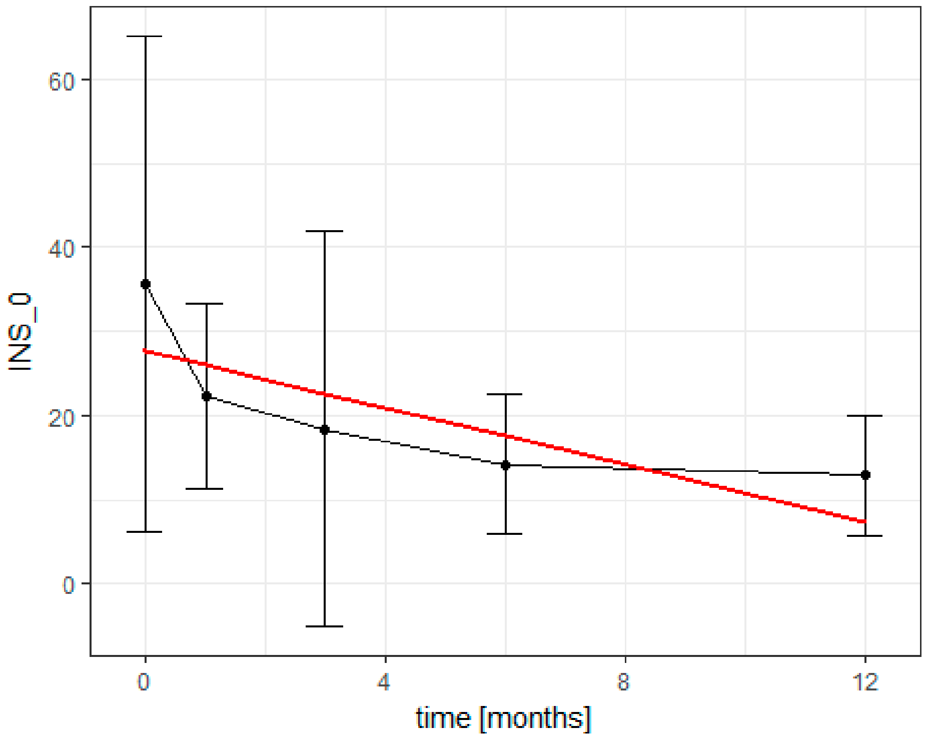

Table 31. Results of linear models for INSULIN CONCETRATION AT 0′ (OGTT)

variable	without covariates	model 1	model 2	model 3	model 4	model 5	model 6	model 7	model 8	model 9	model 10	model 11	model 12	model 13	model 14	model 15
(Intercept)	27.7***	-14.3**	28.5***	23.5***	19.2***	-13.5**	-13.3*	-12.5*	24.4***	20.2***	16.3***	-12.3*	-10.6*	-11.3*	17.6***	-9.3
time	-1.692***	-0.701***	-1.638***	-1.695***	-1.468***	-0.670**	-0.741***	-0.720***	-1.634***	-1.373***	-1.482***	-0.714**	-0.665**	-0.765***	-1.389***	-0.713**
total_mass_kg		0.331***				0.331***	0.318***	0.268***				0.316***	0.260***	0.253***		0.244***
IPAQ			-0.000			-0.000			-0.000	-0.000*		-0.000	-0.000		-0.000*	-0.000
sexM				9.293***			1.377		9.344***		7.301***	1.555		1.540	7.219***	1.660

diet_kcal					0.008***			0.006***		0.008***	0.007***		0.006***	0.006***	0.008***	0.006***
no. of observations	598	595	578	598	559	578	595	556	578	541	559	578	541	556	541	541
AIC	5220.3	5125.4	5071.3	5197.9	4848.3	5006.9	5121.6	4785	5048.6	4716.4	4832.5	5003	4679.9	4781.2	4700.7	4676

Figure 32. Distribution of INSULIN CONCETRATION AT 0′ (OGTT) in time - quadratic fit

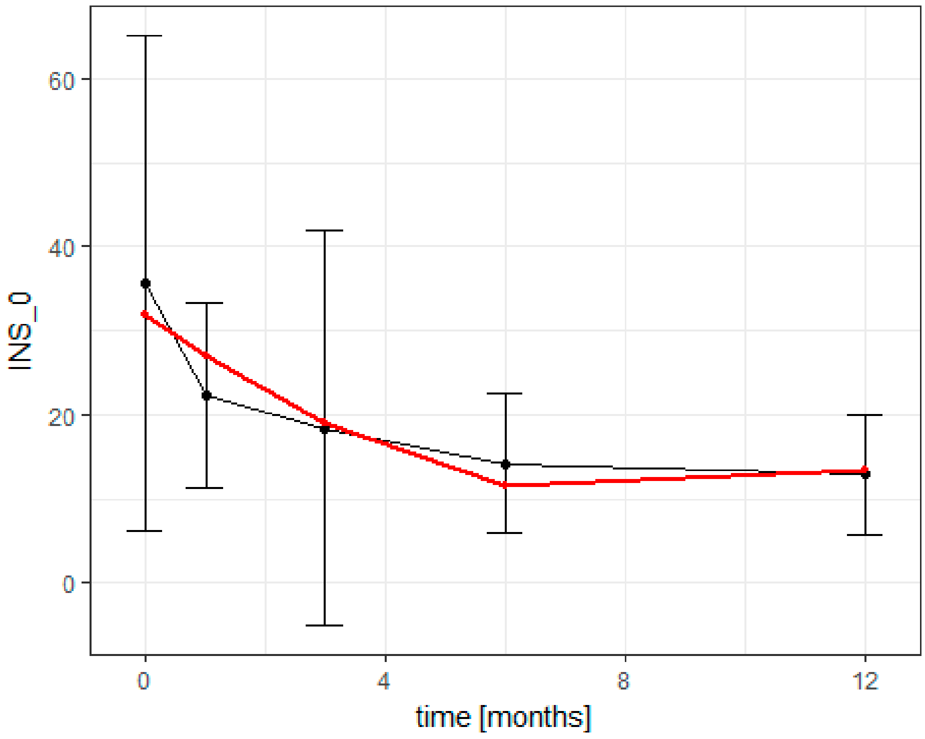

Table 32. Results of quadratic models for INSULIN CONCETRATION AT 0′ (OGTT)

variable	without covariates	model 1	model 2	model 3	model 4	model 5	model 6	model 7	model 8	model 9	model 10	model 11	model 12	model 13	model 14	model 15
(Intercept)	31.8***	-6.3	32.5***	27.6***	23.3***	-5.5	-3.6	-8.6	28.4***	24.2***	20.5***	-2.5	-6.9	-6.3	21.7***	-4.6
time_sq	0.304***	0.182***	0.301***	0.304***	0.176**	0.182***	0.195***	0.098	0.300***	0.168**	0.188***	0.195***	0.093	0.110	0.180**	0.106
time	-5.193***	-2.925***	-5.117***	-5.201***	-3.538***	-2.905***	-3.160***	-1.908**	-5.105***	-3.354***	-3.697***	-3.151***	-1.801*	-2.120**	-3.523***	-2.019**

total_mass_kg		0.287***				0.287***	0.257***	0.254***				0.254***	0.248***	0.230***		0.223***
IPAQ			-0.000			-0.000			-0.000	-0.000*		-0.000	-0.000		-0.000*	-0.000
sexM				9.326***			2.900		9.316***		7.793***	3.049		2.349	7.687***	2.413
diet_kcal					0.006***			0.005***		0.006***	0.006***		0.005***	0.005***	0.006***	0.005***
no. of observations	598	595	578	598	559	578	595	556	578	541	559	578	541	556	541	541
AIC	5187	5116.8	5040.8	5164.3	4842.2	4998.8	5111.6	4785.9	5018.1	4711.7	4824.8	4993.5	4681.2	4781.4	4694.6	4676.5

INSULIN CONCETRATION AT 30′ (OGTT)

Figure 33. Distribution of INSULIN CONCETRATION AT 30′ (OGTT) in time - linear fit

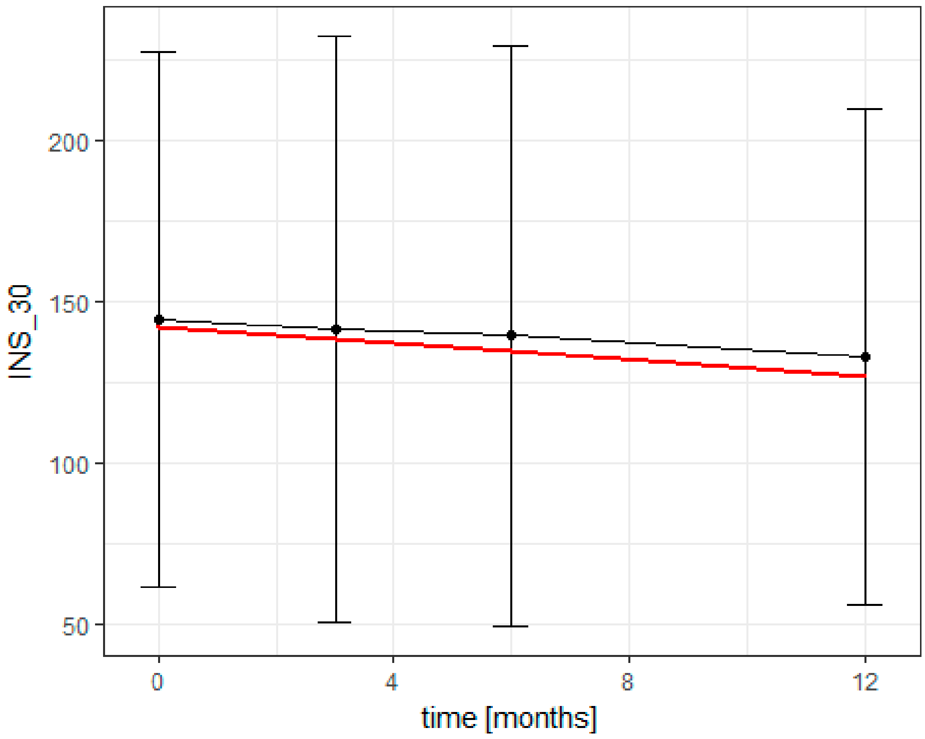

Table 33. Results of linear models for INSULIN CONCETRATION AT 30′ (OGTT)

variable	without covariates	model 1	model 2	model 3	model 4	model 5	model 6	model 7	model 8	model 9	model 10	model 11	model 12	model 13	model 14	model 15
(Intercept)	142.1***	55.9*	148.2***	127.8***	124.3***	64*	61.2*	64.3*	133.2***	128.8***	111.9***	70*	73.5*	72*	115.9***	81.7**
time	-1.261	0.849	-1.060	-1.374*	-0.492	1.020	0.444	0.744	-1.177	-0.228	-0.677	0.576	0.988	0.223	-0.416	0.439
total_mass_kg		0.663***				0.645**	0.560**	0.515*				0.529*	0.482*	0.366		0.323
IPAQ			-0.001			-0.001			-0.001	-0.001		-0.001	-0.001		-0.001	-0.001
sexM				30.864*			17.576		32.185*		29.952*	19.463		22.665	30.946*	23.973
diet_kcal					0.012*			0.008		0.014**	0.011*		0.009	0.009	0.013*	0.010
no. of observations	380	378	373	380	349	373	378	347	373	343	349	373	343	347	343	343

AIC	4277.7	4247.3	4212.2	4264.9	3938.3	4203.2	4238.6	3912.5	4198.9	3883.2	3926.1	4194.1	3879.9	3902.8	3870.7	3869.9

Figure 34. Distribution of INSULIN CONCETRATION AT 30′ (OGTT) in time - quadratic fit

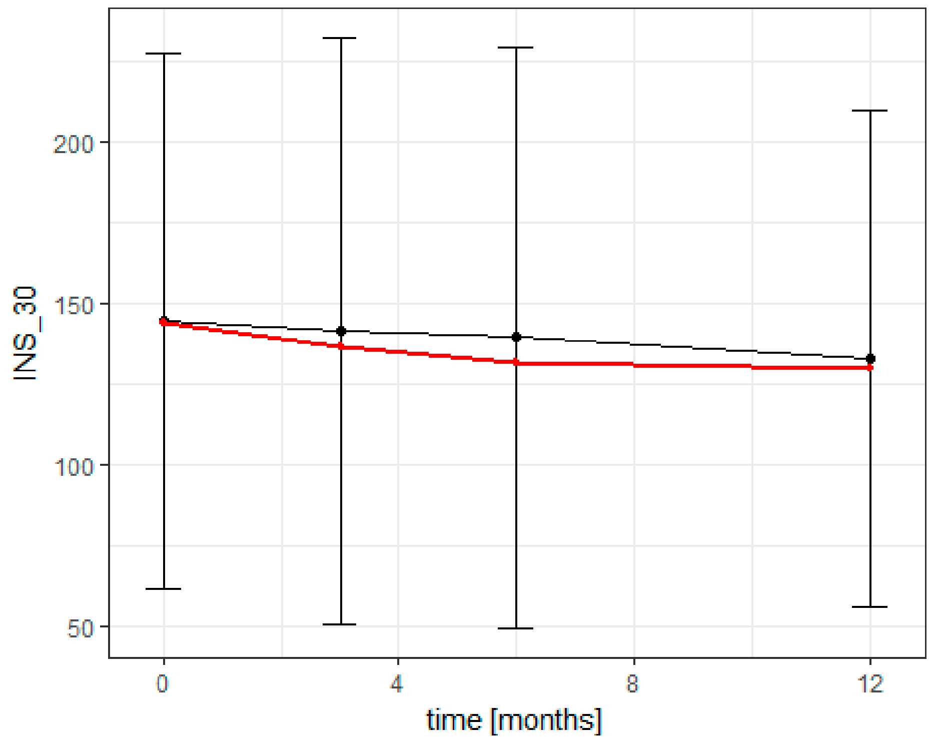

Table 34. Results of quadratic models for INSULIN CONCETRATION AT 30′ (OGTT)

variable	without covariates	model 1	model 2	model 3	model 4	model 5	model 6	model 7	model 8	model 9	model 10	model 11	model 12	model 13	model 14	model 15
(Intercept)	143.8***	37.8	149.6***	129.4***	115.5***	45.2	47.2	27.2	134.7***	115.1***	105.8***	56	32.8	38.2	105.1***	44.3
time_sq	0.146	-0.205	0.120	0.154	-0.231	-0.210	-0.148	-0.495*	0.127	-0.340	-0.176	-0.148	-0.541*	-0.423	-0.285	-0.466
time	-2.899	3.539	-2.411	-3.107	2.402	3.794	2.457	7.331*	-2.612	4.024	1.524	2.585	8.176*	5.995	3.164	6.790
total_mass_kg		0.784***				0.769***	0.663*	0.668**				0.633*	0.646**	0.539*		0.510
IPAQ			-0.001			-0.001			-0.001	-0.001		-0.001	-0.001		-0.001	-0.001
sexM				31.138*			14.815		32.374*		28.771*	16.762		16.203	29.203*	17.093

diet_kcal					0.016**			0.015*		0.020**	0.014*		0.017*	0.015*	0.018**	0.016*
no. of observations	380	378	373	380	349	373	378	347	373	343	349	373	343	347	343	343
AIC	4278.7	4247.6	4213.4	4265.8	3938.4	4203.5	4239.4	3909.3	4200.1	3882.1	3926.6	4195	3876	3900.8	3870.2	3867.4

INSULIN CONCETRATION AT 60′ (OGTT)

Figure 35. Distribution of INSULIN CONCETRATION AT 60′ (OGTT) in time - linear fit

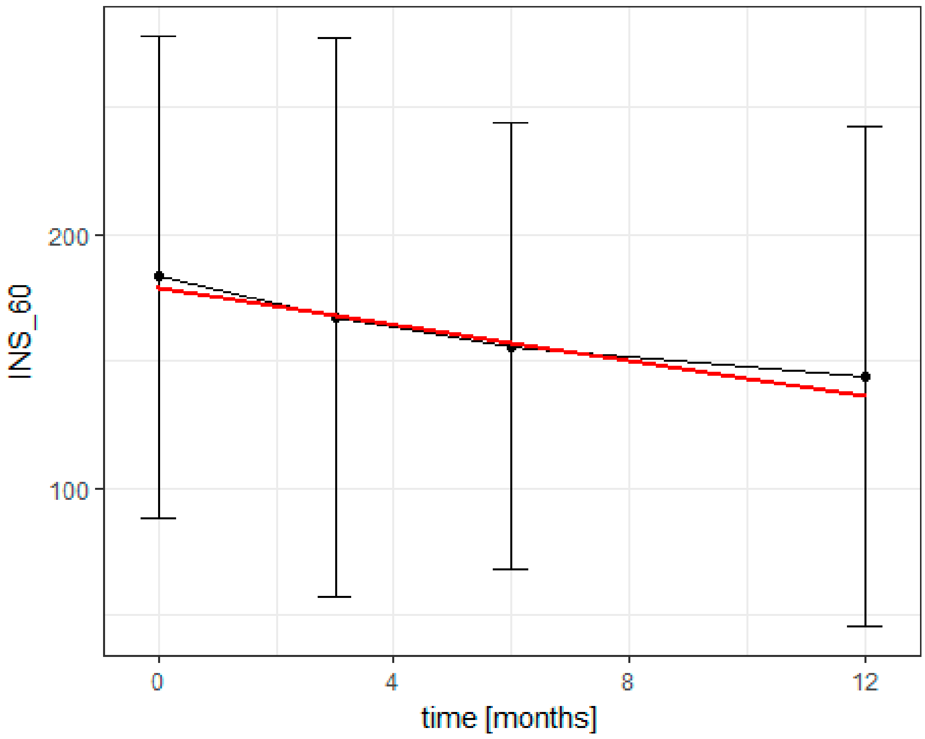

Table 35. Results of linear models for INSULIN CONCETRATION AT 60′ (OGTT)

variable	without covariates	model 1	model 2	model 3	model 4	model 5	model 6	model 7	model 8	model 9	model 10	model 11	model 12	model 13	model 14	model 15
(Intercept)	179***	103.5**	188.3***	170.5***	158.6***	113.9***	105.9**	107.1**	179.6***	166.1***	151.6***	116.6***	118.6***	112**	159.1***	123.3***
time	-3.587***	-1.779	-3.271***	-3.690***	-2.779**	-1.484	-1.936	-1.704	-3.381***	-2.272*	-2.937**	-1.652	-1.253	-2.004	-2.432*	-1.535
total_mass_kg		0.582*				0.569*	0.543*	0.425				0.527*	0.412	0.345		0.335
IPAQ			-0.001*			-0.001*			-0.001*	-0.001*		-0.001*	-0.001*		-0.001*	-0.001*
sexM				18.190			5.539		18.509		17.675	5.916		10.802	17.387	10.110
diet_kcal					0.015*			0.013		0.017*	0.014*		0.013	0.013	0.016*	0.013

no. of observations	379	377	372	379	348	372	377	346	372	342	348	372	342	346	342	342
AIC	4446.9	4420.7	4379.8	4438.1	4087	4375.4	4413.2	4062.6	4371	4028.7	4074.8	4367.9	4027.2	4054.8	4020.1	4019.4

Figure 36. Distribution of INSULIN CONCETRATION AT 60' (OGTT) in time - quadratic fit

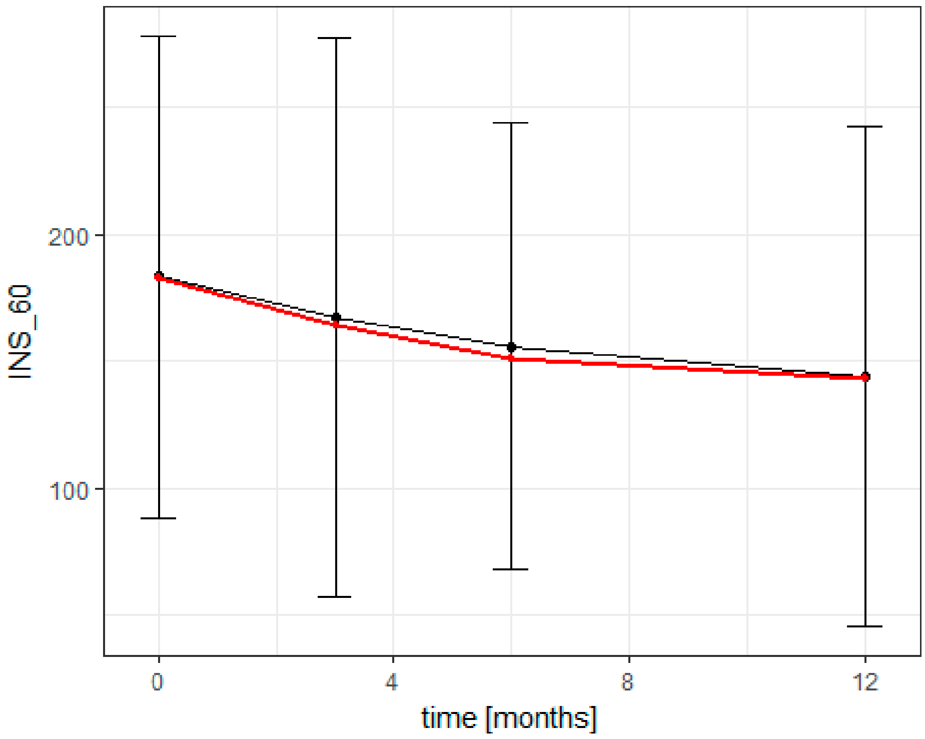

Table 36. Results of quadratic models for INSULIN CONCETRATION AT 60' (OGTT)

variable	without covariates	model 1	model 2	model 3	model 4	model 5	model 6	model 7	model 8	model 9	model 10	model 11	model 12	model 13	model 14	model 15
(Intercept)	183***	111.3**	192.5***	174.3***	161.6***	123.7**	117**	99.4*	183.7***	167.6***	156.2***	130.2**	113.1**	107.3*	162.3***	120.9**
time_sq	0.338	0.111	0.354	0.346	0.081	0.140	0.143	-0.120	0.361	0.039	0.132	0.176	-0.087	-0.067	0.087	-0.035
time	-7.386**	-3.178	-7.255*	-7.583**	-3.789	-3.258	-3.811	-0.138	-7.446**	-2.752	-4.590	-3.953	-0.128	-1.107	-3.522	-1.066
total_mass_kg		0.532*				0.506	0.463	0.452				0.429	0.430	0.366		0.346
IPAQ			-0.001*			-0.001*			-0.001*	-0.001*		-0.001*	-0.001*		-0.001*	-0.001*

sexM				18.766			7.686		19.006		18.525	8.476		9.912	17.892	9.666
diet_kcal					0.014			0.015		0.017	0.012		0.014	0.014	0.015	0.014
no. of observations	379	377	372	379	348	372	377	346	372	342	348	372	342	346	342	342
AIC	4445.9	4421.4	4378.7	4436.9	4087.5	4375.9	4413.8	4062.9	4369.7	4029.2	4078.7	4368.3	4027.5	4055.1	4020.5	4019.7

INSULIN CONCETRATION AT 120′ (OGTT)

Figure 37. Distribution of INSULIN CONCETRATION AT 120′ (OGTT) in time - linear fit

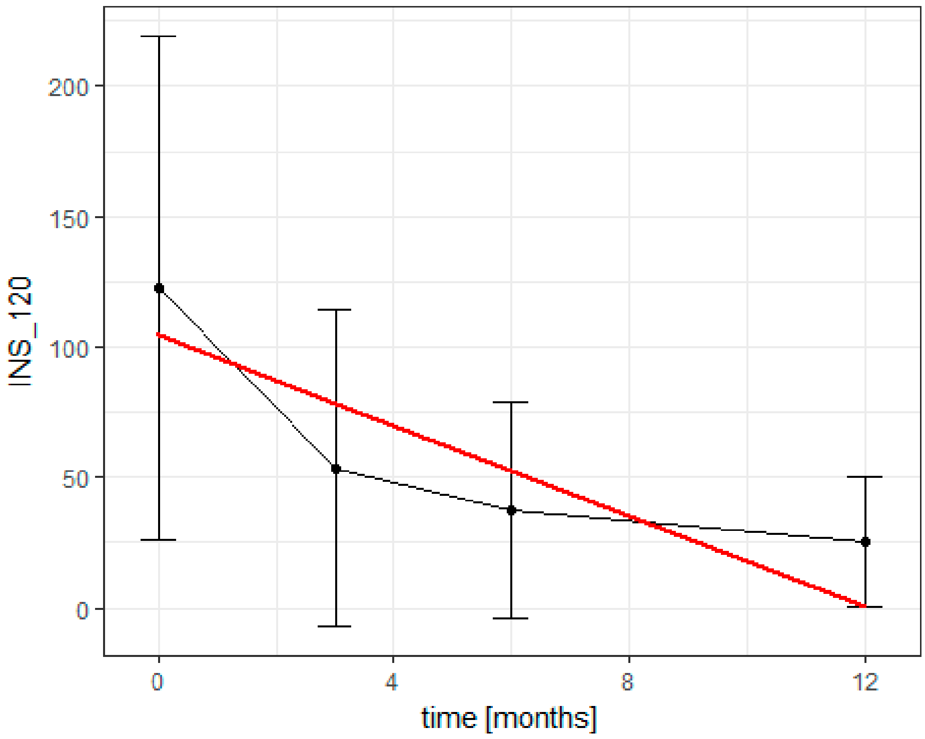

Table 37. Results of linear models for INSULIN CONCETRATION AT 120′ (OGTT)

variable	without covariates	model 1	model 2	model 3	model 4	model 5	model 6	model 7	model 8	model 9	model 10	model 11	model 12	model 13	model 14	model 15
(Intercept)	104.2***	30.3	108.9***	104.7***	77.5***	35.9	17.4	29.1	109.3***	77.7***	79.1***	22.6	37.9	16.7	79.5***	25.1
time	-8.641***	-7.018***	-8.446***	-8.630***	-7.539***	-6.888***	-6.324***	-6.525***	-8.440***	-7.179***	-7.431***	-6.206***	-6.408***	-5.827***	-7.056***	-5.700***
total_mass_kg		0.572**				0.553**	0.736***	0.375*				0.719***	0.336	0.529**		0.493*
IPAQ			-0.000			-0.000			-0.000	-0.001		-0.000	-0.000		-0.001	-0.000
sexM				-0.971			-17.723		-0.757		-5.232	-17.558		-16.720	-5.655	-16.706

diet_kcal					0.019***			0.019**		0.023***	0.019***		0.020**	0.019**	0.023***	0.020**
no. of observations	381	379	373	381	349	373	379	347	373	343	349	373	343	347	343	343
AIC	4326.3	4296.4	4254.3	4320.2	3964.2	4246.5	4286.8	3937.4	4248.2	3909.9	3957.7	4237.1	3908.1	3928.2	3903.3	3898.9

Figure 38. Distribution of INSULIN CONCETRATION AT 120' (OGTT) in time - quadratic fit

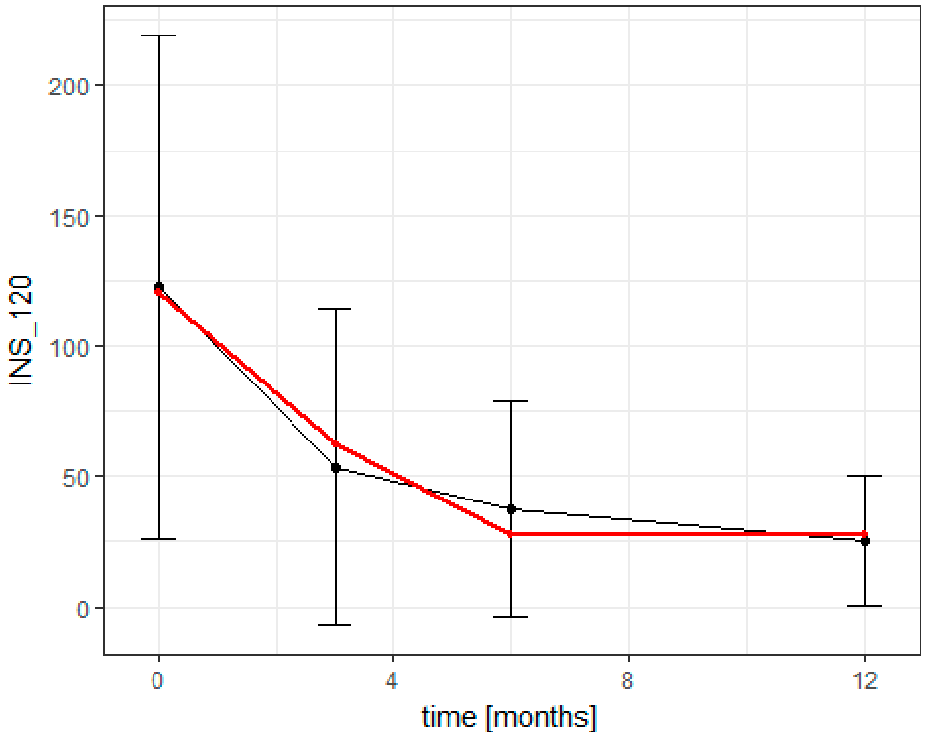

Table 38. Results of quadratic models for INSULIN CONCETRATION AT 120' (OGTT)

variable	without covariates	model 1	model 2	model 3	model 4	model 5	model 6	model 7	model 8	model 9	model 10	model 11	model 12	model 13	model 14	model 15
(Intercept)	120.1***	83.1***	124.6***	119.7***	119.7***	89.5***	76.7**	82.5**	124.2***	120.6***	119.5***	82.7**	91.9***	75.3**	120.6***	84.2**
time_sq	1.277***	1.175***	1.296***	1.278***	1.259***	1.190***	1.141***	1.141***	1.296***	1.226***	1.268***	1.156***	1.170***	1.093***	1.228***	1.119***
time	-23.023***	-21.095***	-23.122***	-23.043***	-22.914***	-21.187***	-20.424***	-20.762***	-23.144***	-22.194***	-23.046***	-20.503***	-20.987***	-19.889***	-22.224***	-20.065***

total_mass_kg		0.278				0.257	0.350	0.263				0.331	0.228	0.328		0.298
IPAQ			-0.000			-0.000			-0.000	-0.001		-0.000	-0.000		-0.001	-0.000
sexM				0.875			-7.010		0.788		1.477	-7.191		-6.561	0.208	-6.965
diet_kcal					0.002			0.003		0.004	0.001		0.003	0.004	0.004	0.004
no. of observations	381	379	373	381	349	373	379	347	373	343	349	373	343	347	343	343
AIC	4292.1	4269.8	4220.9	4286	3944	4220.5	4262.9	3921.6	4214.8	3892.1	3937.8	4213.5	3892.2	3914.7	3886	3885.2

HbA1c

Figure 39. Distribution of HbA1c in time - linear fit

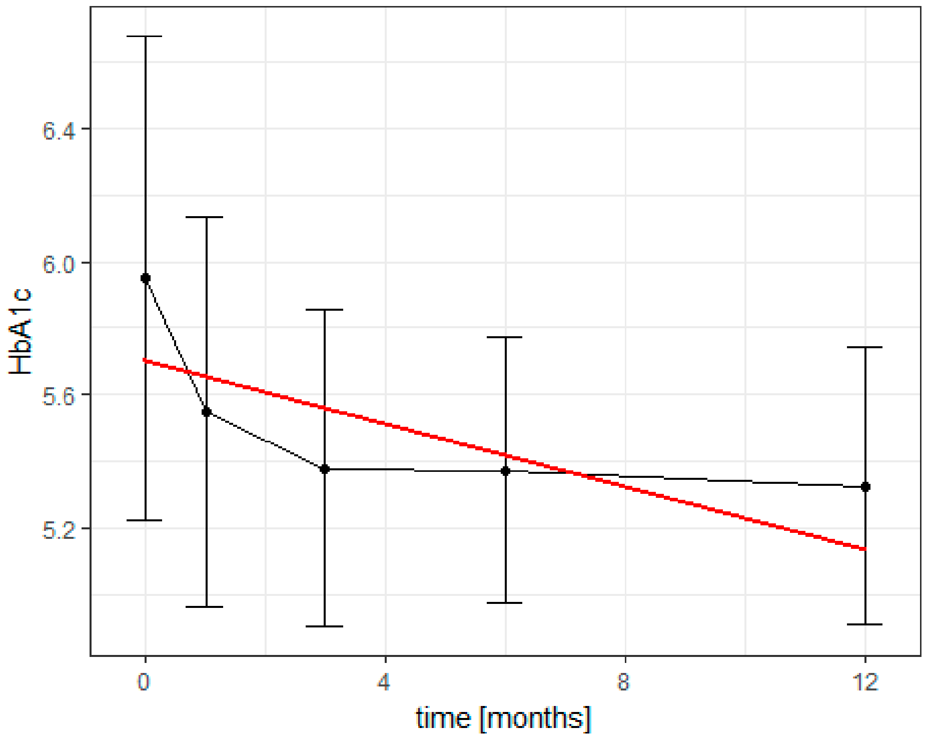

Table 39. Results of linear models for HbA1c

variable	without covariates	model 1	model 2	model 3	model 4	model 5	model 6	model 7	model 8	model 9	model 10	model 11	model 12	model 13	model 14	model 15
(Intercept)	5.7***	4.5***	5.7***	5.7***	5.5***	4.5***	4.5***	4.9***	5.7***	5.5***	5.5***	4.5***	5***	4.9***	5.5***	4.9***
time	-0.047***	-0.020***	-0.048***	-0.048***	-0.043***	-0.022***	-0.015**	-0.031***	-0.048***	-0.043***	-0.043***	-0.017**	-0.031***	-0.027***	-0.043***	-0.027***
total_mass_kg		0.009***				0.009***	0.011***	0.004**				0.011***	0.004**	0.006***		0.006***
IPAQ			0.000			0.000			0.000	-0.000		0.000	-0.000		-0.000	-0.000
sexM				-0.031			-0.302**		-0.022		-0.090	-0.292**		-0.220*	-0.081	-0.211*

diet_kcal					0.000***			0.000***		0.000***	0.000***		0.000***	0.000***	0.000***	0.000***
no. of observations	598	595	578	598	559	578	595	556	578	541	559	578	541	556	541	541
AIC	837.8	806	842.2	840.9	735.3	818.3	799.5	737.8	845.3	751.3	737.2	812.5	755.2	735	753.5	752.9

Figure 40. Distribution of HbA1c in time - quadratic fit

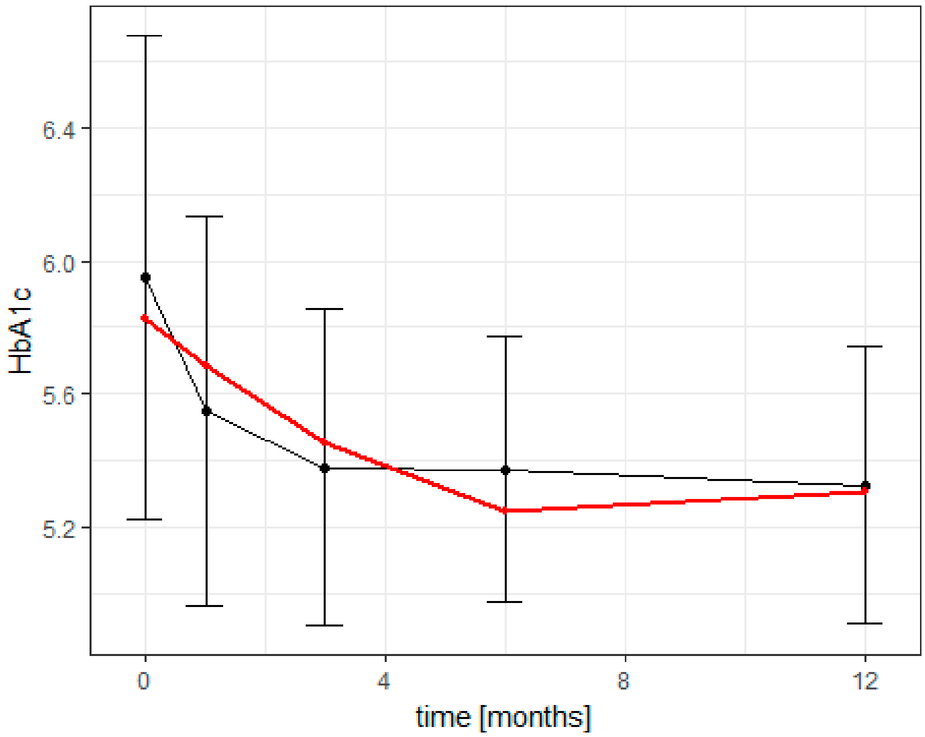

Table 40. Results of quadratic models for HbA1c

variable	without covariates	model 1	model 2	model 3	model 4	model 5	model 6	model 7	model 8	model 9	model 10	model 11	model 12	model 13	model 14	model 15
(Intercept)	5.8***	5.3***	5.8***	5.8***	5.6***	5.3***	5.2***	5.4***	5.8***	5.6***	5.6***	5.2***	5.4***	5.3***	5.6***	5.3***
time_sq	0.009***	0.007***	0.009***	0.009***	0.006***	0.007***	0.007***	0.006***	0.009***	0.006***	0.006***	0.007***	0.006***	0.005***	0.006***	0.005***
time	-0.150***	-0.118***	-0.152***	-0.150***	-0.115***	-0.121***	-0.107***	-0.106***	-0.152***	-0.117***	-0.115***	-0.111***	-0.107***	-0.098***	-0.117***	-0.099***

total_mass_kg		0.004**				0.004**	0.005**	0.001				0.005**	0.001	0.003		0.003
IPAQ			0.000			0.000			0.000	-0.000		0.000	-0.000		-0.000	-0.000
sexM				-0.030			-0.162		-0.024		-0.073	-0.152		-0.132	-0.064	-0.124
diet_kcal					0.000***			0.000***		0.000***	0.000***		0.000***	0.000***	0.000***	0.000***
no. of observations	598	595	578	598	559	578	595	556	578	541	559	578	541	556	541	541
AIC	787	786.3	792.8	790.1	720.1	797.9	786.2	729.7	795.9	737.1	722.5	798.2	747.3	730.5	739.7	748.5

HOMA-beta

Figure 41. Distribution of HOMA-beta in time - linear fit

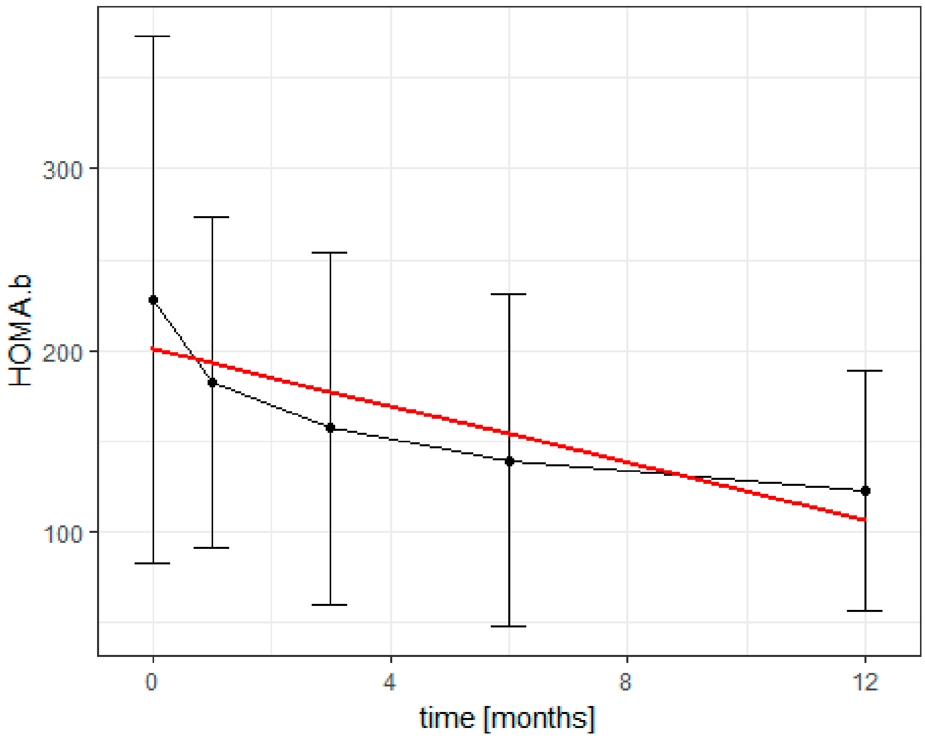

Table 41. Results of linear models for HOMA-beta

variable	without covariates	model 1	model 2	model 3	model 4	model 5	model 6	model 7	model 8	model 9	model 10	model 11	model 12	model 13	model 14	model 15
(Intercept)	201.1***	-98.8***	203.4***	173.6***	170.6***	-96.1***	-97.8***	-68.2*	175.6***	173.2***	147.7***	-94.5**	-57.9*	-65.5*	150.5***	-54.5*
time	-7.864***	-0.869	-7.778***	-7.889***	-6.569***	-0.847	-0.927	-1.042	-7.785***	-6.172***	-6.601***	-0.932	-0.861	-1.204	-6.207***	-1.051
total_mass_kg		2.363***				2.355***	2.344***	2.004***				2.326***	1.948***	1.948***		1.882***
IPAQ			-0.000			-0.000			-0.000	-0.001*		-0.000	-0.001		-0.001*	-0.001
sexM				60.450***			3.133		61.904***		52.242***	4.545		9.103	52.502***	10.418

diet_kcal					0.023***			0.010*		0.026***	0.022***		0.011*	0.010*	0.025***	0.011*
no. of observations	595	592	575	595	556	575	592	553	575	538	556	575	538	553	538	538
AIC	6996.6	6855.7	6779.2	6972.5	6339.9	6673.3	6848.5	6225.9	6753.9	6149.5	6319	6666	6074.9	6218.4	6128.2	6067.2

Figure 42. Distribution of HOMA-beta in time - quadratic fit

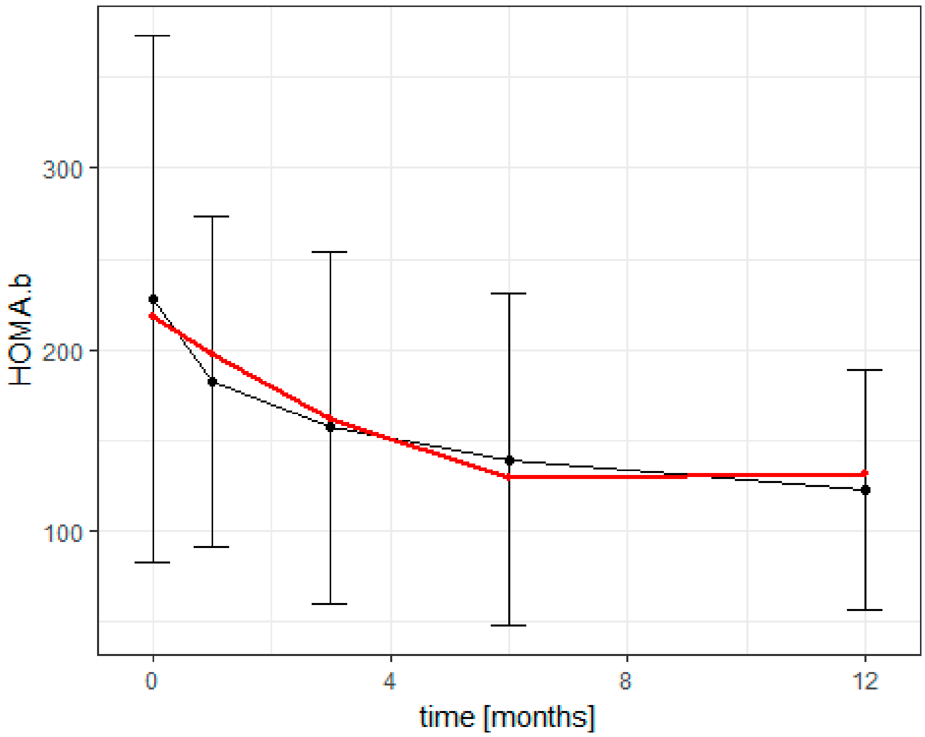

Table 42. Results of quadratic models for HOMA-beta

variable	without covariates	model 1	model 2	model 3	model 4	model 5	model 6	model 7	model 8	model 9	model 10	model 11	model 12	model 13	model 14	model 15
(Intercept)	218.3***	-69.6*	220***	190.6***	189.3***	-67.4*	-63.7	-58.2	192.1***	191***	165.8***	-60.3	-49.3	-52	167.8***	-42.1
time_sq	1.268***	0.361	1.267***	1.266***	0.778***	0.360	0.394	0.124	1.261***	0.727***	0.798***	0.396	0.111	0.159	0.748***	0.150
time	-22.414***	-5.585	-22.405***	-22.427***	-15.716***	-5.552	-6.189*	-2.657	-22.351***	-14.767***	-15.979***	-6.242*	-2.310	-3.323	-15.057***	-3.048

total_mass_kg		2.172***				2.166***	2.095***	1.946***				2.077***	1.899***	1.857***		1.800***
IPAQ			-0.000			-0.000			-0.000	-0.001*		-0.000	-0.001		-0.001*	-0.001
sexM				60.683***			9.330		62.014***		54.536***	10.658		11.599	54.818***	12.695
diet_kcal					0.016***			0.009*		0.019***	0.015***		0.010*	0.009*	0.018***	0.010*
no. of observations	595	592	575	595	556	575	592	553	575	538	556	575	538	553	538	538
AIC	6958.5	6854.2	6742.7	6934.4	6323.6	6671.8	6846.5	6227	6717.8	6136.2	6301.8	6664	6076	6219.2	6113.9	6068

HOMA-IR

Figure 43. Distribution of HOMA-IR in time - linear fit

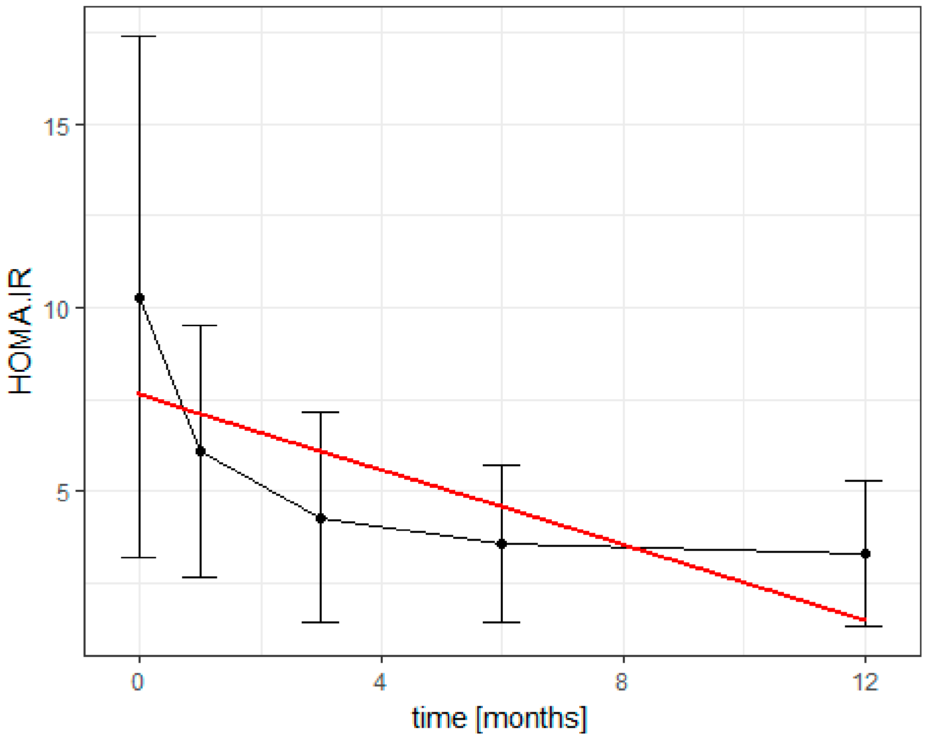

Table 43. Results of linear models for HOMA-IR

variable	without covariates	model 1	model 2	model 3	model 4	model 5	model 6	model 7	model 8	model 9	model 10	model 11	model 12	model 13	model 14	model 15
(Intercept)	7.6***	-5.5***	7.8***	6.6***	4.8***	-5.4***	-5.9***	-4.3***	6.8***	5***	4.2***	-5.8***	-3.7**	-4.5***	4.4***	-4**
time	-0.511***	-0.204***	-0.501***	-0.512***	-0.434***	-0.201***	-0.190***	-0.222***	-0.500***	-0.416***	-0.437***	-0.189***	-0.215***	-0.213***	-0.418***	-0.208***
total_mass_kg		0.103***				0.104***	0.108***	0.077***				0.108***	0.074***	0.080***		0.077***
IPAQ			-0.000			-0.000			-0.000	-0.000*		-0.000	-0.000*		-0.000*	-0.000*
sexM				2.208***			-0.477		2.218***		1.472**	-0.430		-0.328	1.446**	-0.286

diet_kcal					0.003***			0.002***		0.003***	0.003***		0.002***	0.002***	0.003***	0.002***
no. of observations	596	593	576	596	557	576	593	554	576	539	557	576	539	554	539	539
AIC	3426.8	3291.4	3331.3	3408	3071.4	3224.1	3290	2997.7	3312.2	2994.4	3062.4	3222.9	2939.7	2996.8	2985.4	2938.9

Figure 44. Distribution of HOMA-IR in time - quadratic fit

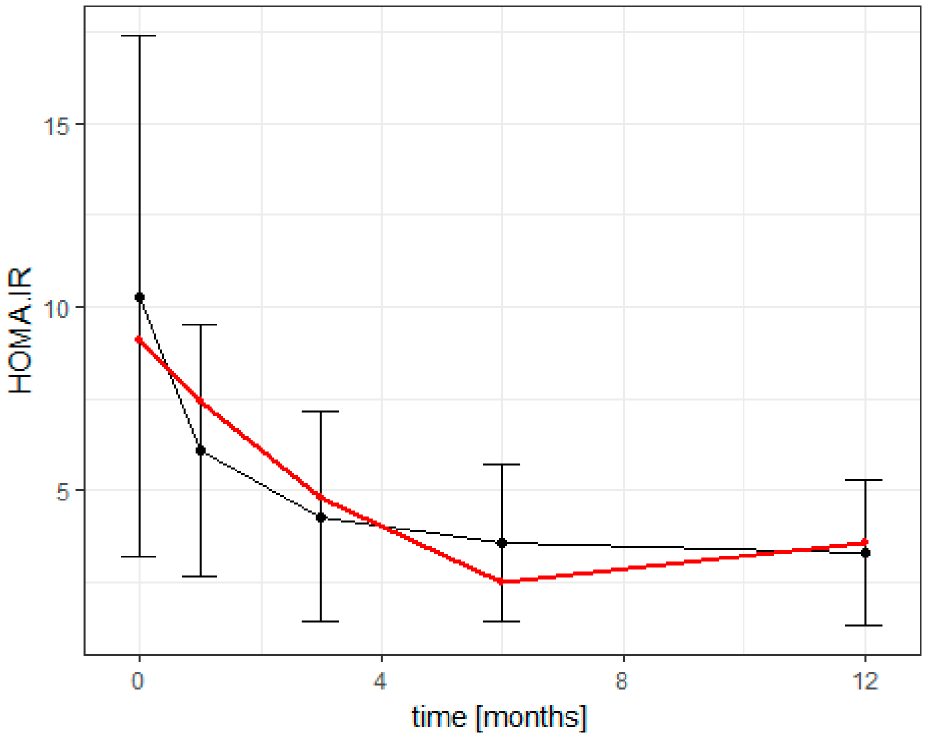

Table 44. Results of quadratic models for HOMA-IR

variable	without covariates	model 1	model 2	model 3	model 4	model 5	model 6	model 7	model 8	model 9	model 10	model 11	model 12	model 13	model 14	model 15
(Intercept)	9.1***	-1.4	9.1***	8.1***	6.4***	-1.3	-1.1	-1.9	8.2***	6.6***	5.7***	-1	-1.4	-1.8	6***	-1.3
time_sq	0.107***	0.073***	0.106***	0.107***	0.068***	0.073***	0.074***	0.048***	0.105***	0.067***	0.070***	0.074***	0.047***	0.049***	0.069***	0.048***
time	-1.737***	-1.112***	-1.724***	-1.738***	-1.236***	-1.114***	-1.137***	-0.816***	-1.720***	-1.206***	-1.258***	-1.141***	-0.804***	-0.827***	-1.230***	-0.817***

total_mass_kg		0.079***				0.079***	0.076***	0.066***				0.076***	0.064***	0.064***		0.062***
IPAQ			-0.000			-0.000			-0.000	-0.000		-0.000	-0.000		-0.000*	-0.000
sexM				2.223***			0.329		2.217***		1.655**	0.359		0.151	1.630**	0.166
diet_kcal					0.002***			0.002***		0.002***	0.002***		0.002***	0.002***	0.002***	0.002***
no. of observations	596	593	576	596	557	576	593	554	576	539	557	576	539	554	539	539
AIC	3331.9	3250.9	3242.1	3313	3037	3185.4	3250	2984.7	3223.2	2964	3025.9	3184.3	2927.7	2984.1	2952.9	2927.1

TOTAL CHOLESTEROL

Figure 45. Distribution of TOTAL CHOLESTEROL in time - linear fit

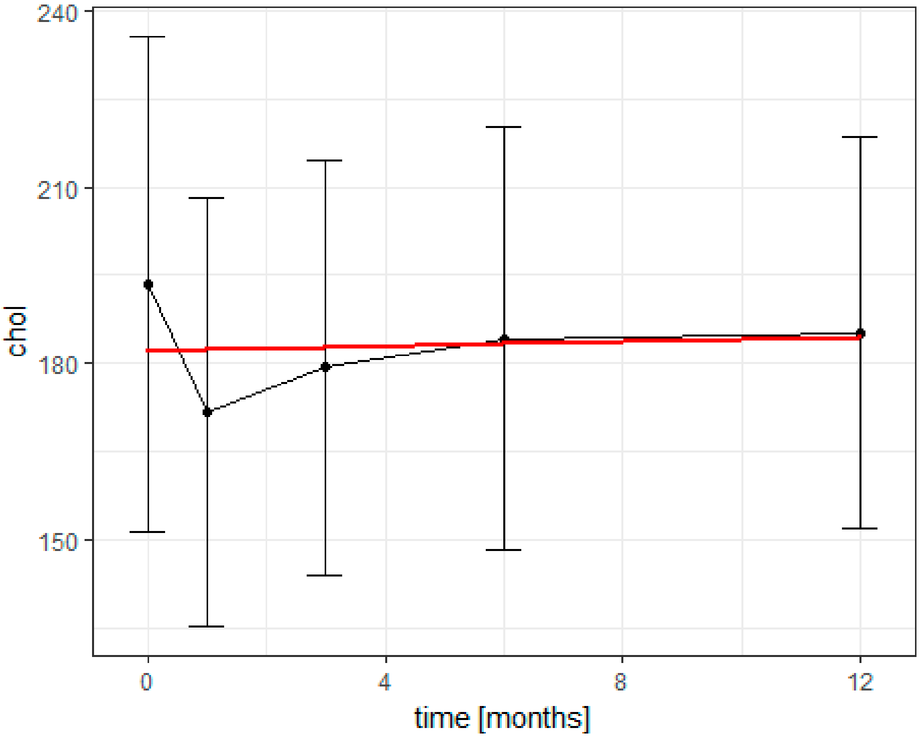

Table 45. Results of linear models for TOTAL CHOLESTEROL

variable	without covariates	model 1	model 2	model 3	model 4	model 5	model 6	model 7	model 8	model 9	model 10	model 11	model 12	model 13	model 14	model 15
(Intercept)	182.2***	178.3***	182***	185.1***	171.6***	177.8***	175.4***	194.7***	184.7***	170.8***	175.4***	174.7***	194.9***	192.9***	174.5***	192.9***
time	0.193	0.271	0.133	0.194	0.508	0.231	0.431	-0.082	0.133	0.484	0.511	0.391	-0.076	0.013	0.488	0.024
total_mass_kg		0.031				0.033	0.084	-0.203*				0.086	-0.203*	-0.170		-0.168
IPAQ			0.000			0.000			0.000	0.000		0.000	0.000		0.000	0.000
sexM				-6.473			-8.354		-6.022		-8.820	-8.160		-4.827	-8.790	-5.007
diet_kcal					0.010***			0.012***		0.011***	0.010***		0.012***	0.012***	0.011***	0.012***

no. of observations	602	599	581	602	563	581	599	560	581	544	563	581	544	560	544	544
AIC	5825.8	5802.2	5646	5819.2	5434	5648.9	5794.8	5400.8	5639.5	5273.3	5426.2	5641.7	5271.6	5394.7	5265.5	5265.5

Figure 46. Distribution of TOTAL CHOLESTEROL in time - quadratic fit

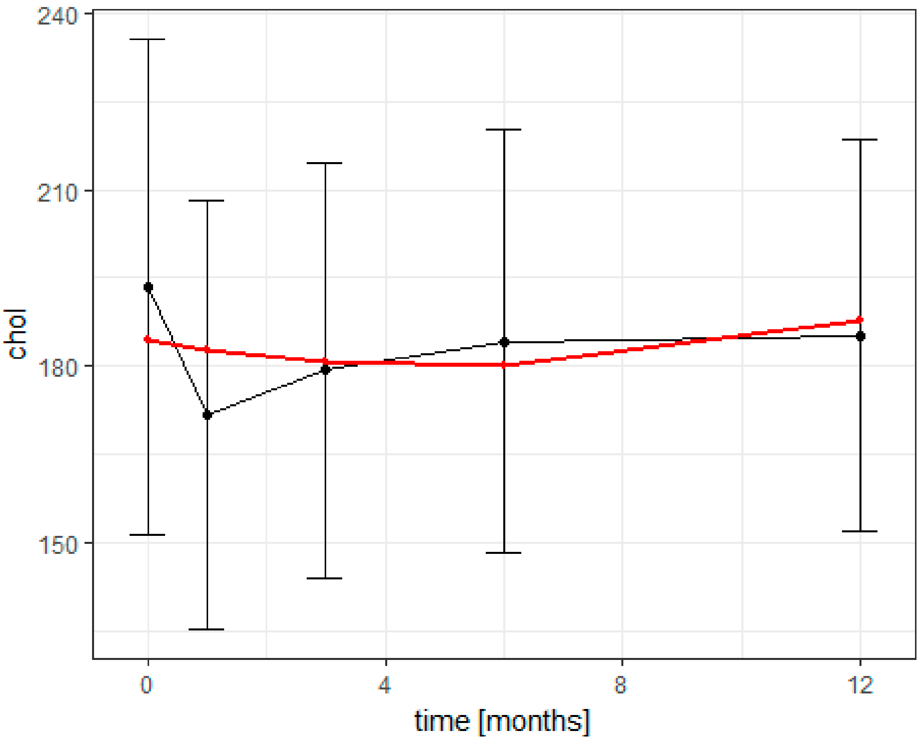

Table 46. Results of quadratic models for TOTAL CHOLESTEROL

variable	without covariates	model 1	model 2	model 3	model 4	model 5	model 6	model 7	model 8	model 9	model 10	model 11	model 12	model 13	model 14	model 15
(Intercept)	184.5***	197.9***	184.4***	187.5***	171.5***	197.1***	194.7***	199.3***	187.1***	170.4***	175.2***	193.8***	199***	196.7***	174***	196.1***
time_sq	0.173*	0.219**	0.183*	0.173*	-0.005	0.223**	0.202*	0.060	0.183*	-0.016	-0.010	0.206*	0.056	0.046	-0.022	0.041
time	-1.799*	-2.642*	-1.977*	-1.797*	0.561	-2.722*	-2.320	-0.855	-1.979*	0.676	0.623	-2.408*	-0.801	-0.595	0.746	-0.524
total_mass_kg		-0.099				-0.096	-0.058	-0.229*				-0.055	-0.227*	-0.195		-0.190

IPAQ			0.000			0.000			0.000	0.000		0.000	0.000		0.000	0.000
sexM				-6.450			-4.797		-6.044		-8.846	-4.685		-4.141	-8.852	-4.410
diet_kcal					0.010***			0.012***		0.011***	0.010***		0.012***	0.012***	0.011***	0.012***
no. of observations	602	599	581	602	563	581	599	560	581	544	563	581	544	560	544	544
AIC	5823.4	5798.3	5643.2	5816.8	5437.2	5645.1	5792.3	5403.4	5636.7	5276.5	5429.4	5639.1	5274.2	5397.5	5268.6	5268.3

TRIGLYCERIDES

Figure 47. Distribution of TRIGLYCERIDES in time - linear fit

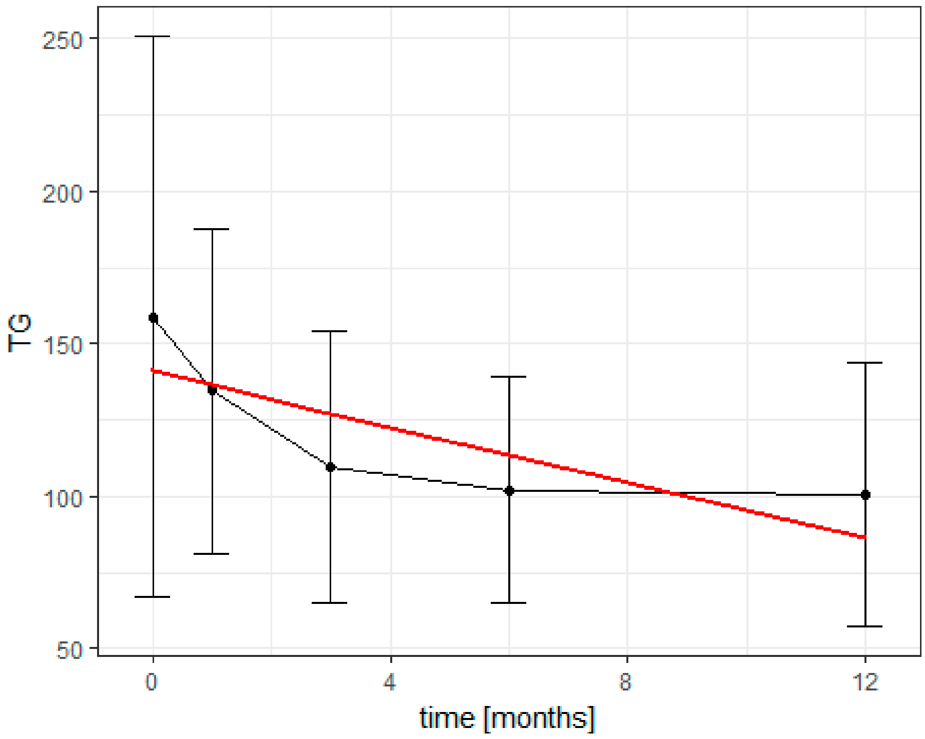

Table 47. Results of linear models for TRIGLYCERIDES

variable	without covariates	model 1	model 2	model 3	model 4	model 5	model 6	model 7	model 8	model 9	model 10	model 11	model 12	model 13	model 14	model 15
(Intercept)	140.9***	44.4*	142.7***	134.5***	119***	45.1*	41*	63.9***	136.3***	118***	115.4***	41.3*	66.6***	62.9***	114.7***	65.2***
time	-4.548***	-2.324***	-4.544***	-4.552***	-4.030***	-2.292***	-2.185**	-2.824***	-4.546***	-3.862***	-4.035***	-2.140**	-2.677***	-2.784***	-3.870***	-2.624***
total_mass_kg		0.763***				0.766***	0.809***	0.445**				0.818***	0.434**	0.459**		0.453**
IPAQ			-0.000			-0.000			-0.000	-0.000		-0.000	-0.000		-0.000	-0.000
sexM				14.099			-5.330		14.312		8.616	-5.932		-1.478	8.062	-2.138

diet_kcal					0.021***			0.020***		0.025***	0.021***		0.022***	0.020***	0.025***	0.022***
no. of observations	602	599	581	602	563	581	599	560	581	544	563	581	544	560	544	544
AIC	6533.6	6476.7	6335.6	6524.3	6009.8	6309.8	6470.1	5962.3	6326.3	5821.1	6002.6	6303.1	5814.1	5956.1	5814.1	5808

Figure 48. Distribution of TRIGLYCERIDES in time - quadratic fit

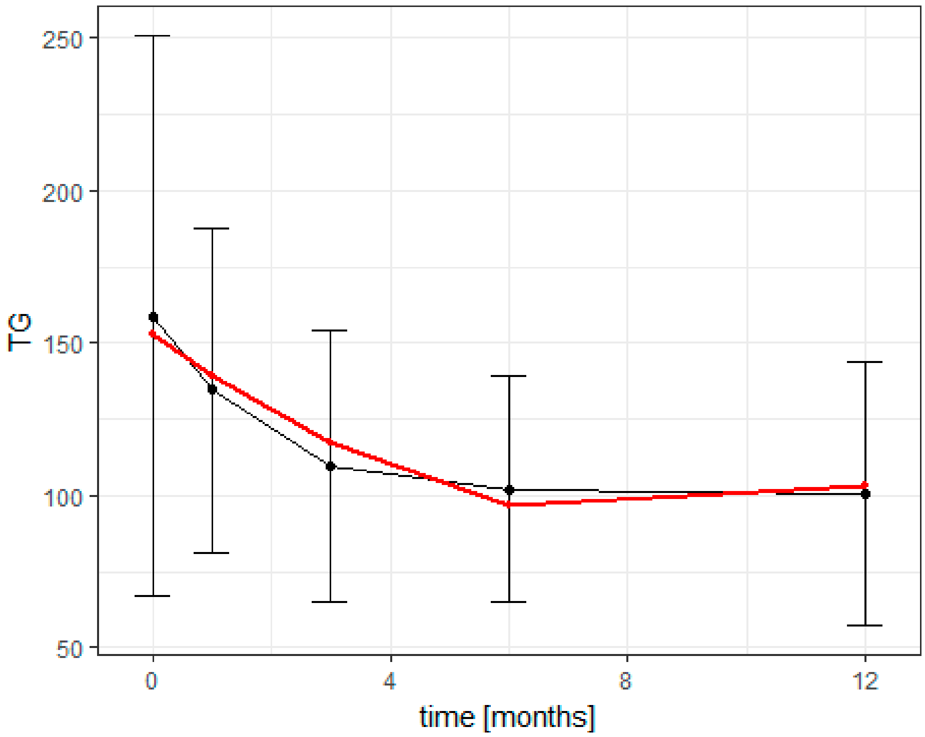

Table 48. Results of quadratic models for TRIGLYCERIDES

variable	without covariates	model 1	model 2	model 3	model 4	model 5	model 6	model 7	model 8	model 9	model 10	model 11	model 12	model 13	model 14	model 15
(Intercept)	152.7***	94.1***	154.4***	146.2***	132.2***	94***	97.8***	91.6***	148.1***	130.1***	128.1***	97***	92***	94.6***	126.4***	94.2***
time_sq	0.862***	0.687***	0.886***	0.863***	0.554***	0.696***	0.706***	0.431***	0.885***	0.501***	0.564***	0.710***	0.408***	0.446***	0.510***	0.419***
time	-14.471***	-11.178***	-14.773***	-14.486***	-10.562***	-11.243***	-11.529***	-8.304***	-14.772***	-9.786***	-10.677***	-11.516***	-7.862***	-8.594***	-9.903***	-8.067***

total_mass_kg		0.446**				0.455**	0.401*	0.299*				0.420*	0.303*	0.262		0.276
IPAQ			-0.000			-0.000			-0.000	-0.000		-0.000	-0.000		-0.000	-0.000
sexM				14.219			4.842		14.177		10.165	3.826		4.194	9.471	3.016
diet_kcal					0.016***			0.018***		0.020***	0.016***		0.019***	0.018***	0.020***	0.019***
no. of observations	602	599	581	602	563	581	599	560	581	544	563	581	544	560	544	544
AIC	6495	6456.9	6297.5	6485.7	5995.7	6290.5	6450.4	5955.4	6288.2	5810.6	5988	6284.1	5808.5	5949	5803.1	5802.2

HDL CHOLESTEROL

Figure 49. Distribution of HDL CHOLESTEROL in time - linear fit

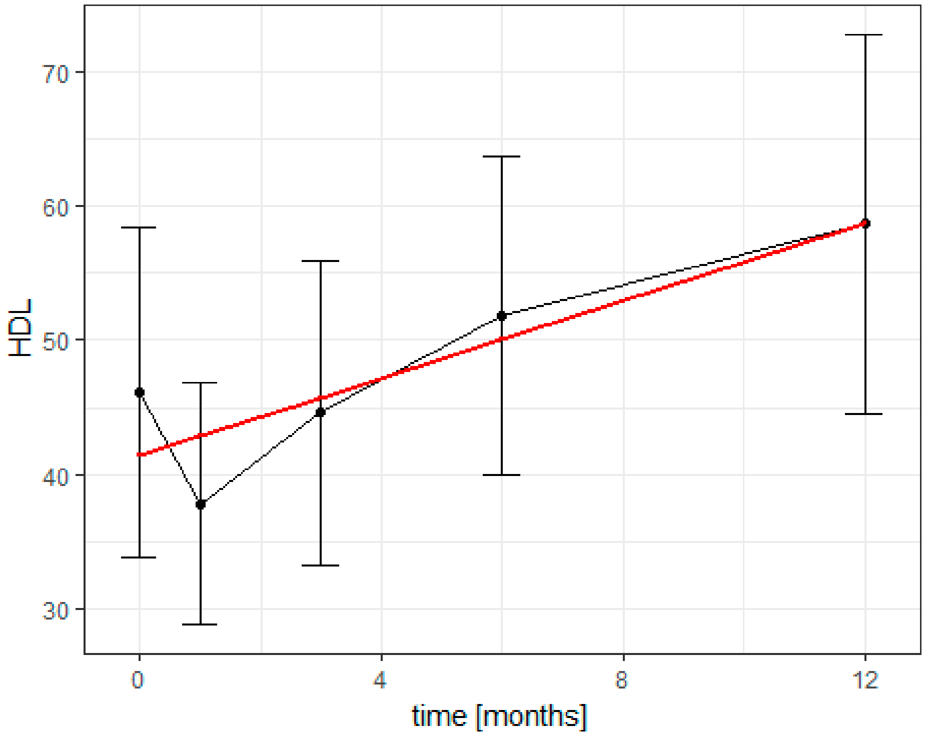

Table 49. Results of linear models for HDL CHOLESTEROL

variable	without covariates	model 1	model 2	model 3	model 4	model 5	model 6	model 7	model 8	model 9	model 10	model 11	model 12	model 13	model 14	model 15
(Intercept)	41.4***	55.2***	41.2***	45.1***	36.7***	54.6***	52.7***	61.2***	44.8***	36.6***	40.7***	51.9***	60.8***	59.1***	40.5***	58.6***
time	1.434***	1.110***	1.399***	1.436***	1.557***	1.085***	1.242***	0.973***	1.399***	1.545***	1.561***	1.218***	0.985***	1.079***	1.549***	1.094***
total_mass_kg		-0.108***				-0.106***	-0.065*	-0.208***				-0.061*	-0.204***	-0.171***		-0.165***
IPAQ			0.000			0.000			0.000	0.000		0.000	0.000		0.000	0.000
sexM				-8.195***			-6.569***		-8.103***		-9.262***	-6.582***		-5.369**	-9.207***	-5.475***

diet_kcal					0.004***			0.006***		0.004***	0.004***		0.006***	0.006***	0.004***	0.006***
no. of observations	598	595	577	598	560	577	595	557	577	541	560	577	541	557	541	541
AIC	4424.1	4395.9	4290.4	4396.3	4095.6	4282.7	4377.2	4023.1	4263	3980.3	4062.4	4264.1	3935.9	4009.4	3947.5	3921.9

Figure 50. Distribution of HDL CHOLESTEROL in time - quadratic fit

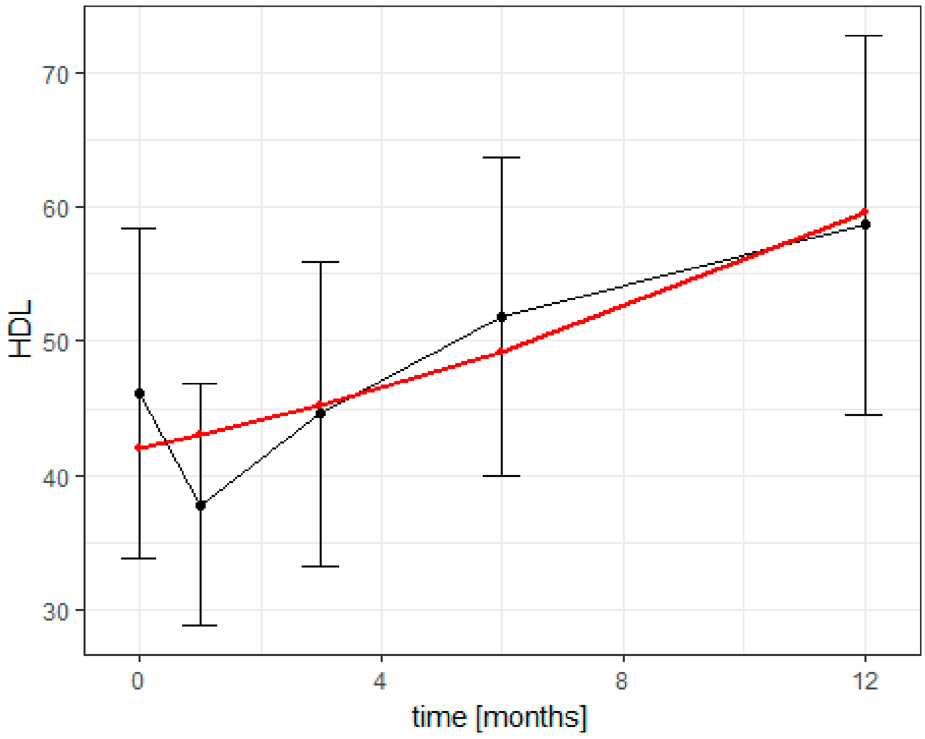

Table 50. Results of quadratic models for HDL CHOLESTEROL

variable	without covariates	model 1	model 2	model 3	model 4	model 5	model 6	model 7	model 8	model 9	model 10	model 11	model 12	model 13	model 14	model 15
(Intercept)	42***	64.1***	41.9***	45.7***	35.8***	63.8***	60.6***	63.7***	45.5***	35.9***	39.8***	60.1***	63.7***	60.4***	39.7***	60.4***
time_sq	0.045*	0.115***	0.051*	0.044	-0.036	0.119***	0.096***	0.035	0.051*	-0.030	-0.041	0.101***	0.041	0.017	-0.035	0.024
time	0.915**	-0.382	0.806**	0.923***	1.984***	-0.471	-0.032	0.523	0.810**	1.898***	2.042***	-0.124	0.454	0.851*	1.962***	0.781*

total_mass_kg		-0.166***				-0.165***	-0.122***	-0.222***				-0.121***	-0.220***	-0.179***		-0.177***
IPAQ			0.000			0.000			0.000	0.000		0.000	0.000		0.000	0.000
sexM				-8.187***			-5.136**		-8.107***		-9.376***	-5.115**		-5.127**	-9.308***	-5.154**
diet_kcal					0.004***			0.006***		0.005***	0.005***		0.006***	0.006***	0.005***	0.005***
no. of observations	598	595	577	598	560	577	595	557	577	541	560	577	541	557	541	541
AIC	4426	4382.3	4291.2	4398.3	4098.9	4268	4369.8	4026.8	4263.9	3984.4	4065.1	4255.6	3938.8	4014.4	3951	3926.5

LDL CHOLESTEROL

Figure 51. Distribution of LDL CHOLESTEROL in time - linear fit

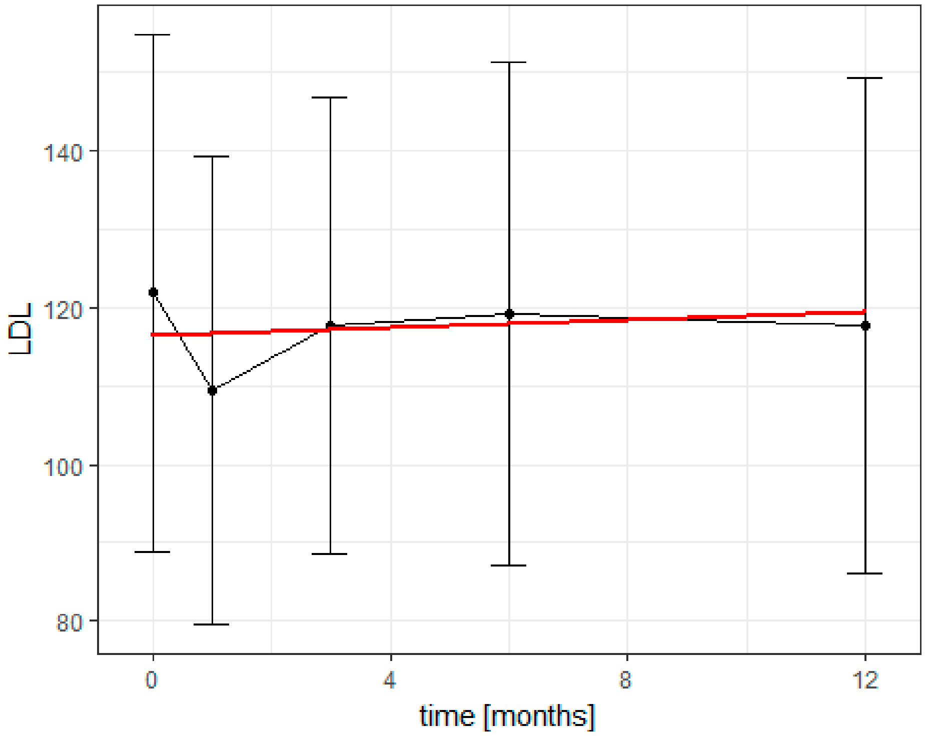

Table 51. Results of linear models for LDL CHOLESTEROL

variable	without covariates	model 1	model 2	model 3	model 4	model 5	model 6	model 7	model 8	model 9	model 10	model 11	model 12	model 13	model 14	model 15
(Intercept)	116.4***	111.7***	115.3***	117.4***	111.4***	110.1***	110.6***	117.7***	116.2***	110.4***	112.9***	108.9***	116***	116.8***	111.8***	115***
time	0.260	0.361	0.161	0.261	0.427	0.283	0.427	0.253	0.162	0.332	0.428	0.349	0.203	0.299	0.334	0.253
total_mass_kg		0.037				0.041	0.059	-0.055				0.063	-0.047	-0.039		-0.029
IPAQ			0.000			0.000			0.000	0.000		0.000	0.000		0.000	0.000
sexM				-2.233			-3.627		-2.060		-3.397	-3.625		-2.423	-3.312	-2.653
diet_kcal					0.004**			0.005**		0.004**	0.004**		0.005**	0.005**	0.004**	0.005**

no. of observations	602	599	581	602	563	581	599	560	581	544	563	581	544	560	544	544
AIC	5572.7	5551.4	5399.8	5567.6	5223.2	5403	5545.8	5200.2	5394.8	5070.5	5217.7	5397.4	5073.4	5195	5065.1	5068.1

Figure 52. Distribution of LDL CHOLESTEROL in time - quadratic fit

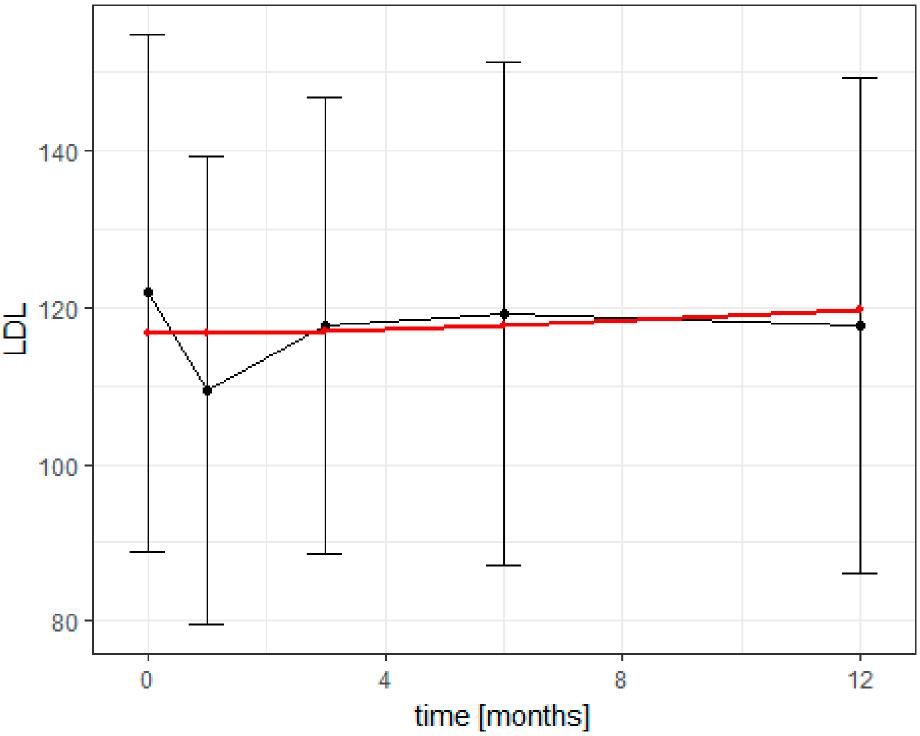

Table 52. Results of quadratic models for LDL CHOLESTEROL

variable	without covariates	model 1	model 2	model 3	model 4	model 5	model 6	model 7	model 8	model 9	model 10	model 11	model 12	model 13	model 14	model 15
(Intercept)	116.5***	111.3***	115.5***	117.6***	109.3***	110***	108.9***	110.6***	116.5***	108.2***	110.8***	107.6***	109.3***	108.2***	109.7***	106.7***
time_sq	0.013	-0.005	0.017	0.013	-0.089	-0.001	-0.018	-0.091	0.017	-0.092	-0.091	-0.014	-0.089	-0.104	-0.094	-0.103
time	0.109	0.423	-0.030	0.110	1.477	0.294	0.671	1.443	-0.030	1.421	1.503	0.536	1.365	1.687	1.450	1.618
total_mass_kg		0.040				0.042	0.072	-0.014				0.073	-0.009	0.018		0.025

IPAQ			0.000			0.000			0.000	0.000		0.000	0.000		0.000	0.000
sexM				-2.231			-3.942		-2.061		-3.650	-3.858		-4.011	-3.580	-4.167
diet_kcal					0.005***			0.006***		0.005**	0.005***		0.005**	0.006***	0.005**	0.005**
no. of observations	602	599	581	602	563	581	599	560	581	544	563	581	544	560	544	544
AIC	5576.5	5554.9	5403.5	5571.4	5224.9	5406.5	5549.3	5202	5398.5	5072.2	5219.4	5400.8	5075.3	5196.3	5066.7	5069.5

ASPARTATE TRANSAMINASE

Figure 53. Distribution of ASPARTATE TRANSAMINASE in time - linear fit

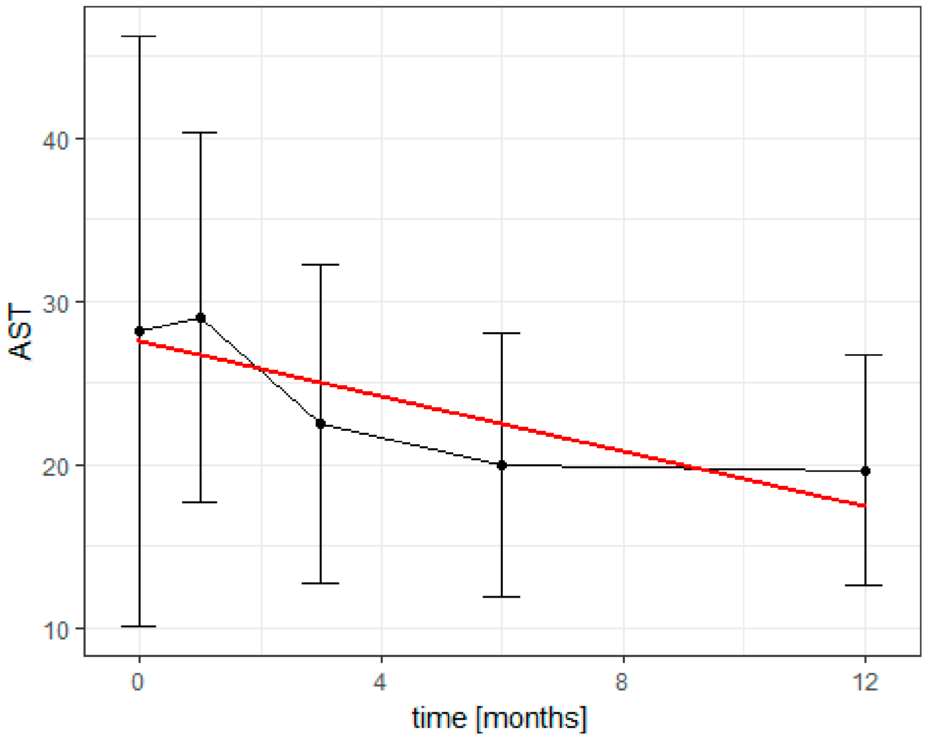

Table 53. Results of linear models for ASPARTATE TRANSAMINASE

variable	without covariates	model 1	model 2	model 3	model 4	model 5	model 6	model 7	model 8	model 9	model 10	model 11	model 12	model 13	model 14	model 15
(Intercept)	27.5***	12.2***	27.1***	25.3***	27.1***	12.6***	13.6***	11.9***	25.2***	27.3***	24.9***	13.9***	12.7***	13.6***	25.4***	14.4***
time	-0.843***	-0.470***	-0.796***	-0.844***	-0.783***	-0.463***	-0.523***	-0.404***	-0.796***	-0.735***	-0.787***	-0.513***	-0.401***	-0.471***	-0.740***	-0.466***
total_mass_kg		0.118***				0.114***	0.100***	0.133***				0.097***	0.123***	0.110***		0.100***
IPAQ			-0.000			-0.000			-0.000	-0.000		-0.000	-0.000		-0.000	-0.000
sexM				4.843***			1.993		4.273**		5.258***	1.873		2.586	4.762***	2.523

diet_kcal					-0.000			-0.002**		-0.000	-0.000		-0.001*	-0.002**	-0.001	-0.001*
no. of observations	600	597	579	600	561	579	597	558	579	542	561	579	542	558	542	542
AIC	4614.1	4541.6	4434.5	4600.3	4117.2	4418.7	4537.2	4025.6	4421.3	3937.9	4100	4414.5	3916.8	4019.6	3919.9	3911

Figure 54. Distribution of ASPARTATE TRANSAMINASE in time - quadratic fit

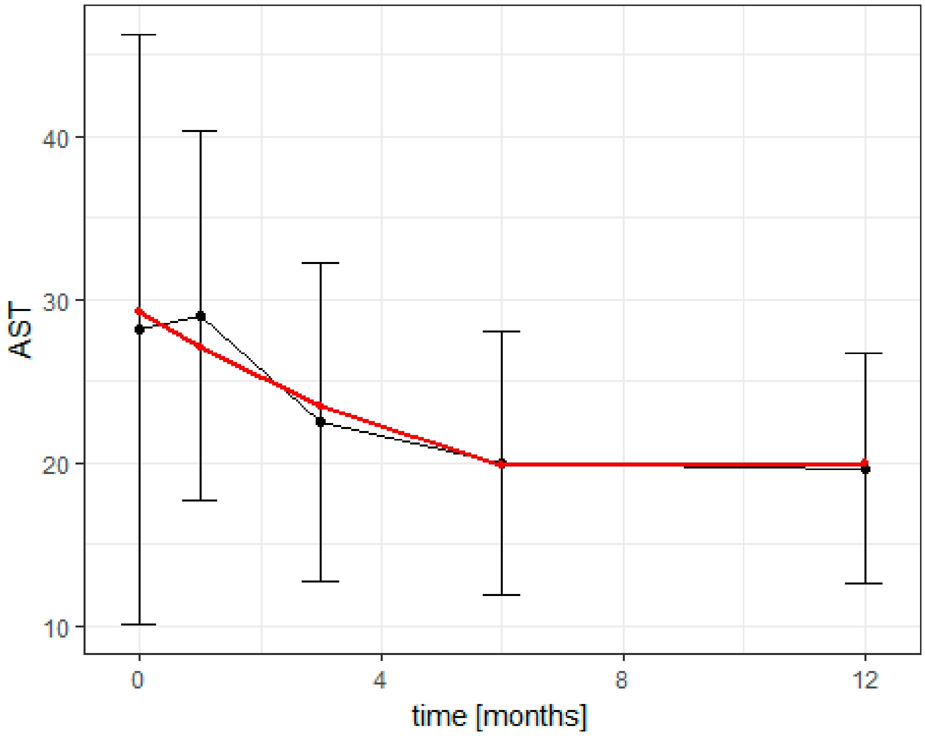

Table 54. Results of quadratic models for ASPARTATE TRANSAMINASE

variable	without covariates	model 1	model 2	model 3	model 4	model 5	model 6	model 7	model 8	model 9	model 10	model 11	model 12	model 13	model 14	model 15
(Intercept)	29.3***	16.6***	28.7***	27.1***	30.3***	17***	19.1***	17.9***	26.9***	30.5***	28***	19.5***	18.3***	21***	28.5***	21.4***
time_sq	0.131***	0.083**	0.122***	0.131***	0.135***	0.085**	0.095**	0.105***	0.122***	0.132***	0.139***	0.097**	0.102***	0.121***	0.137***	0.117***
time	-2.357***	-1.505***	-2.209***	-2.359***	-2.379***	-1.526***	-1.730***	-1.724***	-2.203***	-2.296***	-2.430***	-1.740***	-1.684***	-2.011***	-2.360***	-1.961***

total_mass_kg		0.093***				0.089***	0.064*	0.104***				0.060*	0.097***	0.067*		0.061*
IPAQ			-0.000			0.000			-0.000	-0.000		-0.000	-0.000		-0.000	-0.000
sexM				4.855***			2.906		4.255**		5.647***	2.766		3.884**	5.152***	3.735**
diet_kcal					-0.001*			-0.003***		-0.002**	-0.001*		-0.002***	-0.003***	-0.002***	-0.002***
no. of observations	600	597	579	600	561	579	597	558	579	542	561	579	542	558	542	542
AIC	4598.3	4539.2	4421.9	4584.5	4095.2	4416	4532.7	4014.6	4408.8	3916.8	4076.2	4409.9	3906.8	4004.3	3896.5	3896.9

ALANINE TRANSAMINASE

Figure 55. Distribution of ALANINE TRANSAMINASE in time - linear fit

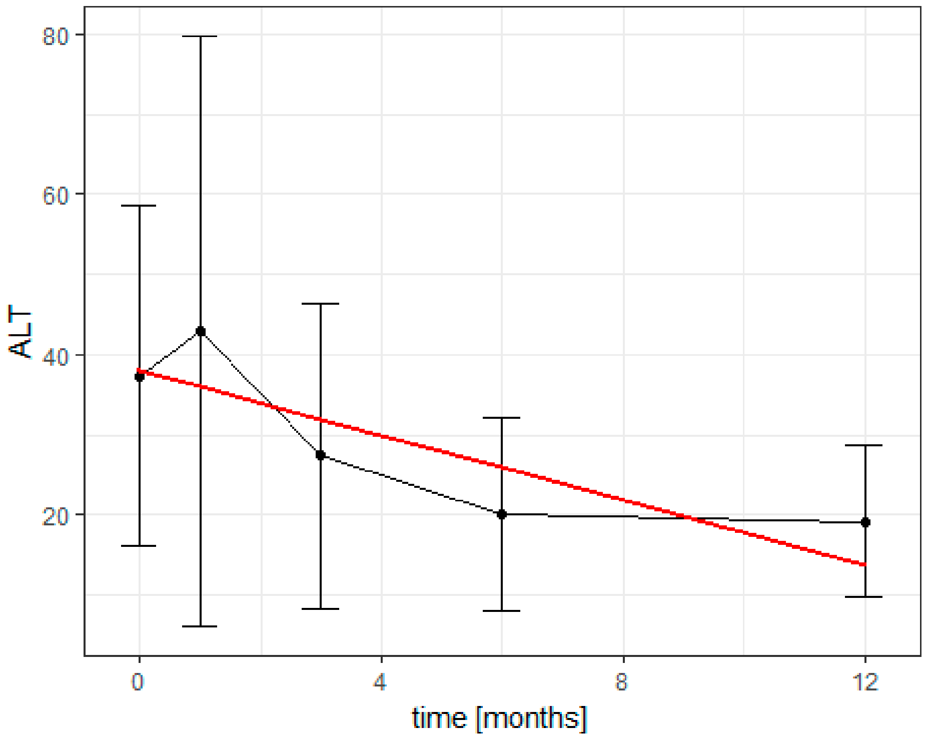

Table 55. Results of linear models for ALANINE TRANSAMINASE

variable	without covariates	model 1	model 2	model 3	model 4	model 5	model 6	model 7	model 8	model 9	model 10	model 11	model 12	model 13	model 14	model 15
(Intercept)	38***	4.3	38.6***	32.4***	39***	4.1	9.5	4.9	33.3***	39.5***	34.1***	9.3	4.7	10.2	34.9***	9.8
time	-2.014***	-1.223***	-1.937***	-2.014***	-2.035***	-1.142***	-1.425***	-1.217***	-1.936***	-1.956***	-2.048***	-1.334***	-1.155***	-1.420***	-1.976***	-1.346***
total_mass_kg		0.265***				0.272***	0.198***	0.290***				0.206***	0.293***	0.221***		0.228***
IPAQ			-0.000			-0.000			-0.000	-0.000		-0.000	-0.000		-0.000	-0.000
sexM				12.322***			7.348**		12.095***		12.352***	6.983*		7.227*	11.972***	6.748*

diet_kcal					-0.001			-0.003*		-0.001	-0.001		-0.003	-0.003*	-0.001	-0.003
no. of observations	602	599	581	602	563	581	599	560	581	544	563	581	544	560	544	544
AIC	5451.9	5400.6	5276.8	5425.1	5131.1	5250.4	5389.5	5077.9	5250.7	4972.3	5105.1	5240	4945.4	5067.4	4947.6	4935.6

Figure 56. Distribution of ALANINE TRANSAMINASE in time - quadratic fit

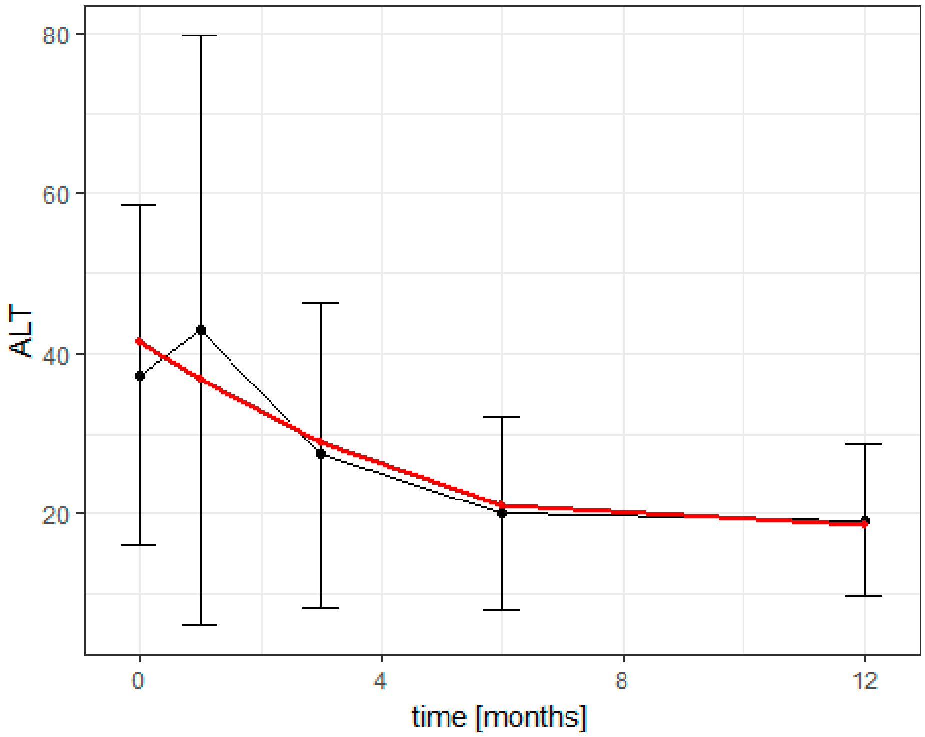

Table 56. Results of quadratic models for ALANINE TRANSAMINASE

variable	without covariates	model 1	model 2	model 3	model 4	model 5	model 6	model 7	model 8	model 9	model 10	model 11	model 12	model 13	model 14	model 15
(Intercept)	41.4***	11.5	41.8***	35.8***	46.3***	10.7	19.4**	15.1*	36.5***	46.7***	41.4***	18.5*	14*	23.6**	42.2***	22.2**
time_sq	0.247***	0.153*	0.237***	0.249***	0.309***	0.143*	0.191**	0.235***	0.238***	0.298***	0.328***	0.178**	0.221**	0.280***	0.318***	0.263***
time	-4.865***	-3.108***	-4.683***	-4.888***	-5.677***	-2.898***	-3.816***	-4.092***	-4.689***	-5.481***	-5.922***	-3.564***	-3.861***	-4.901***	-5.741***	-4.614***

total_mass_kg		0.225***				0.235***	0.135*	0.251***				0.148**	0.259***	0.157**		0.170**
IPAQ			-0.000			-0.000			-0.000	-0.000		-0.000	-0.000		-0.000	-0.000
sexM				12.360***			8.935**		12.065***		13.248***	8.407**		9.468**	12.821***	8.774**
diet_kcal					-0.004*			-0.005***		-0.003*	-0.004**		-0.005**	-0.005***	-0.004**	-0.005**
no. of observations	602	599	581	602	563	581	599	560	581	544	563	581	544	560	544	544
AIC	5437.8	5398.2	5264.6	5410.6	5113.7	5248.9	5384	5069.9	5238.4	4957.2	5084.8	5235.8	4939	5055	4929.7	4925.5

C REACTIVE PROTEIN

Figure 57. Distribution of C REACTIVE PROTEIN in time - linear fit

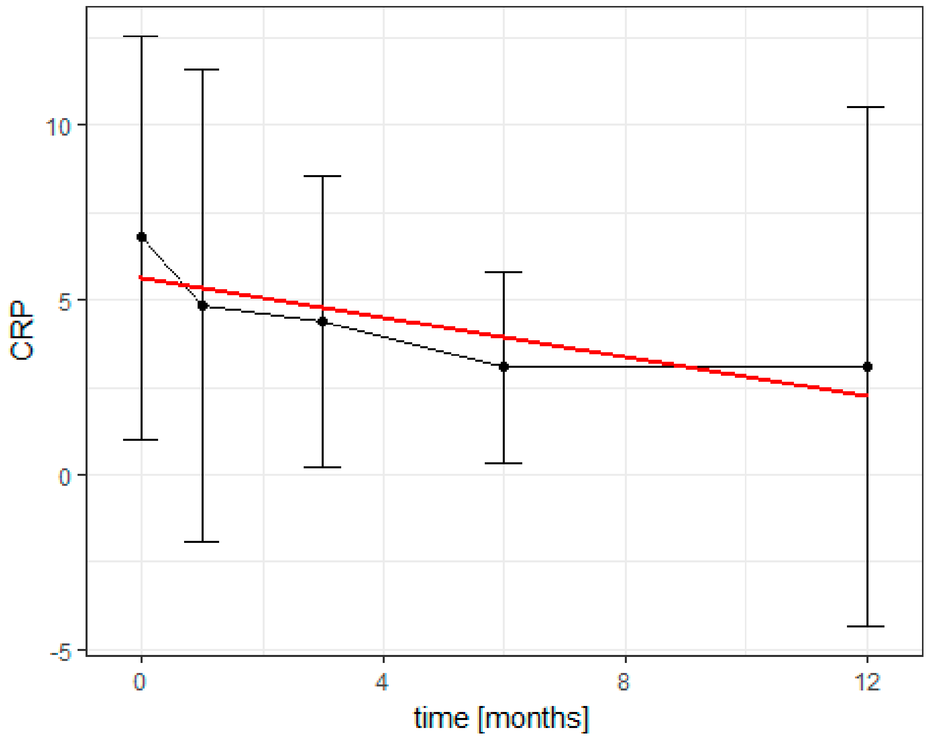

Table 57. Results of linear models for C REACTIVE PROTEIN

variable	without covariates	model 1	model 2	model 3	model 4	model 5	model 6	model 7	model 8	model 9	model 10	model 11	model 12	model 13	model 14	model 15
(Intercept)	5.6***	0.4	5.6***	5.6***	4.4***	0.1	-0.6	0.4	5.7***	4.7***	4.5***	-1	0.3	-0.6	4.8***	-0.8
time	-0.281***	-0.148*	-0.270***	-0.281***	-0.250***	-0.145*	-0.111	-0.141*	-0.270***	-0.240***	-0.249***	-0.103	-0.138*	-0.104	-0.239***	-0.097
total_mass_kg		0.041***				0.043***	0.053***	0.036**				0.057***	0.037**	0.049***		0.052***
IPAQ			0.000			0.000			0.000	-0.000		0.000	-0.000		-0.000	-0.000
sexM				0.038			-1.364*		-0.141		-0.204	-1.550*		-1.326	-0.320	-1.485*
diet_kcal					0.001***			0.001		0.001*	0.001***		0.001	0.000	0.001*	0.001

no. of observations	598	595	577	598	559	577	595	556	577	540	559	577	540	556	540	540
AIC	3703.5	3665.7	3587.8	3702.6	3477.5	3581.8	3660.6	3449.9	3586.8	3378.6	3476.5	3575.7	3377	3445	3377.4	3371.3

Figure 58. Distribution of C REACTIVE PROTEIN in time - quadratic fit

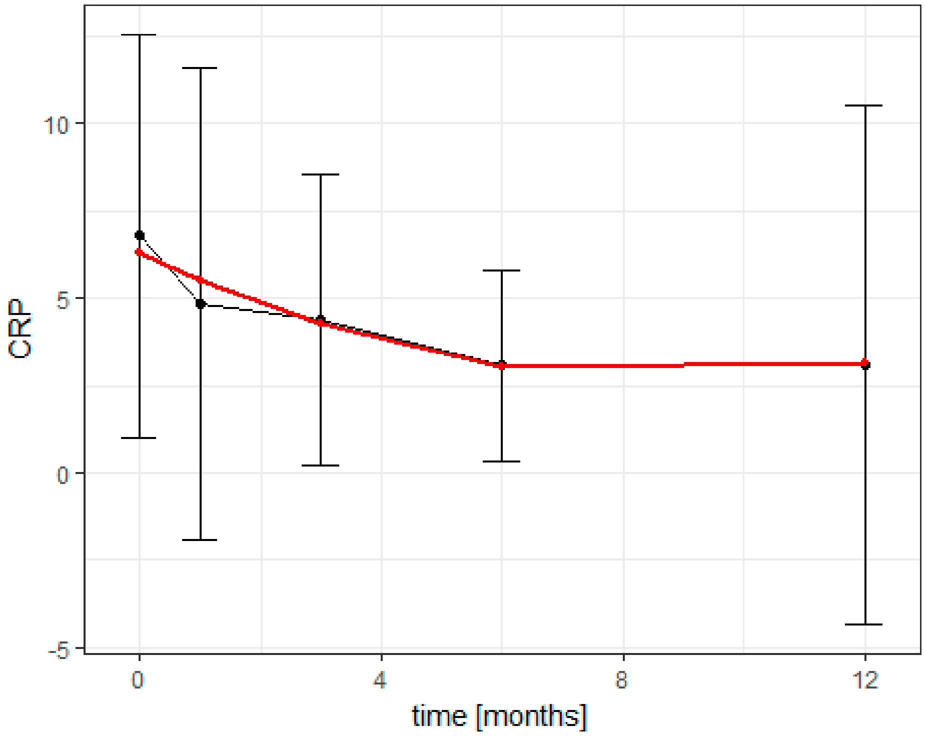

Table 58. Results of quadratic models for C REACTIVE PROTEIN

variable	without covariates	model 1	model 2	model 3	model 4	model 5	model 6	model 7	model 8	model 9	model 10	model 11	model 12	model 13	model 14	model 15
(Intercept)	6.3***	1.9	6.2***	6.3***	5.1***	1.8	0.9	1.5	6.3***	5.5***	5.1***	0.6	1.4	0.4	5.6***	0.2
time_sq	0.047***	0.032*	0.047***	0.047***	0.029	0.033*	0.027	0.025	0.047***	0.036*	0.029	0.028	0.027	0.019	0.036*	0.020
time	-0.820***	-0.541**	-0.817***	-0.820***	-0.590**	-0.559**	-0.450*	-0.443*	-0.817***	-0.665***	-0.587**	-0.455*	-0.465*	-0.341	-0.660**	-0.352
total_mass_kg		0.032**				0.033**	0.044**	0.032*				0.047**	0.033*	0.044**		0.046**
IPAQ			0.000			0.000			0.000	-0.000		0.000	-0.000		-0.000	-0.000

sexM				0.044			-1.124		-0.148		-0.130	-1.301		-1.170	-0.231	-1.319
diet_kcal					0.001*			0.000		0.001	0.001*		0.000	0.000	0.001	0.000
no. of observations	598	595	577	598	559	577	595	556	577	540	559	577	540	556	540	540
AIC	3699.2	3667.7	3583.4	3698.3	3480.7	3583.5	3664	3454	3582.4	3380.1	3479.7	3579	3380.8	3450.1	3379	3376.2

MEAN INSULIN CONCENTRATION (OGTT)

Figure 59. Distribution of MEAN INSULIN CONCENTRATION (OGTT) in time - linear fit

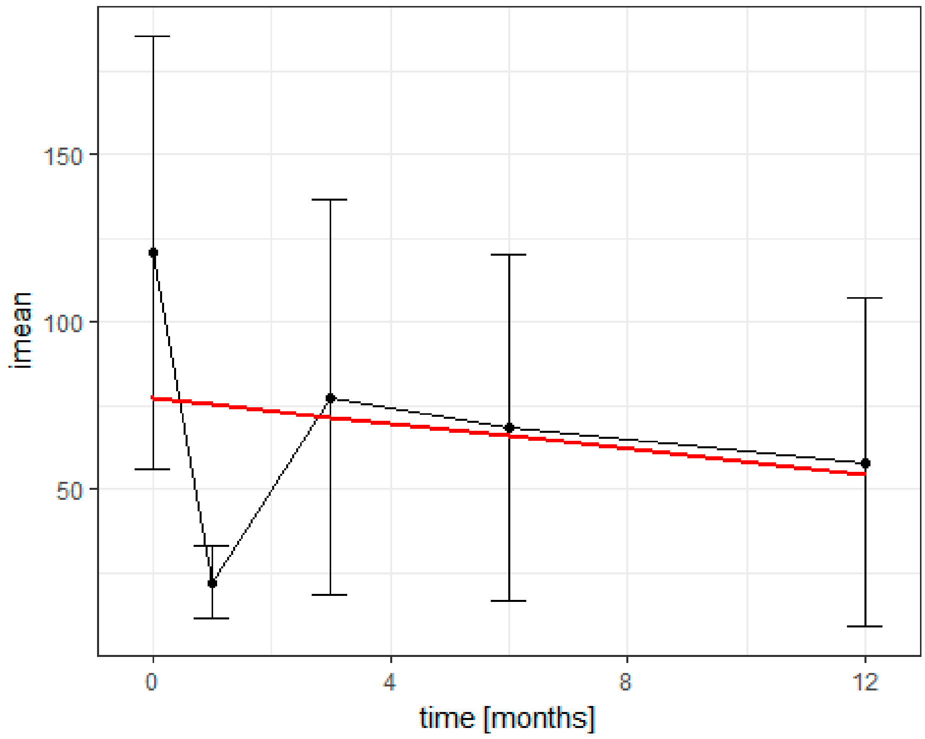

Table 59. Results of linear models for MEAN INSULIN CONCENTRATION (OGTT)

variable	without covariates	model 1	model 2	model 3	model 4	model 5	model 6	model 7	model 8	model 9	model 10	model 11	model 12	model 13	model 14	model 15
(Intercept)	77.5***	-23.9	77***	67***	32.9***	-32.6	-20.4	-9	65.9***	31.2***	27.5***	-28.3	-11.2	-3.7	25.3***	-5
time	-1.951***	0.378	-2.224***	-1.966***	-0.954	0.278	0.240	-0.093	-2.220***	-1.145*	-0.977	0.123	-0.163	-0.311	-1.169*	-0.405
total_mass_kg		0.800***				0.862***	0.755***	0.332*				0.809***	0.361**	0.259		0.277*
IPAQ			0.000			0.000			0.000	0.000		0.000	0.000		0.000	0.000
sexM				23.096***			4.773		25.528***		13.720*	5.589		8.180	15.515*	9.282

diet_kcal					0.041***			0.042***		0.044***	0.040***		0.041***	0.042***	0.043***	0.041***
no. of observations	598	595	578	598	559	578	595	556	578	541	559	578	541	556	541	541
AIC	6551.8	6482.1	6350.2	6533.5	5974.8	6308.2	6475.9	5927.3	6330.2	5794.6	5964.5	6301.8	5789	5920.2	5783.3	5781.5

Figure 60. Distribution of MEAN INSULIN CONCENTRATION (OGTT) in time - quadratic fit

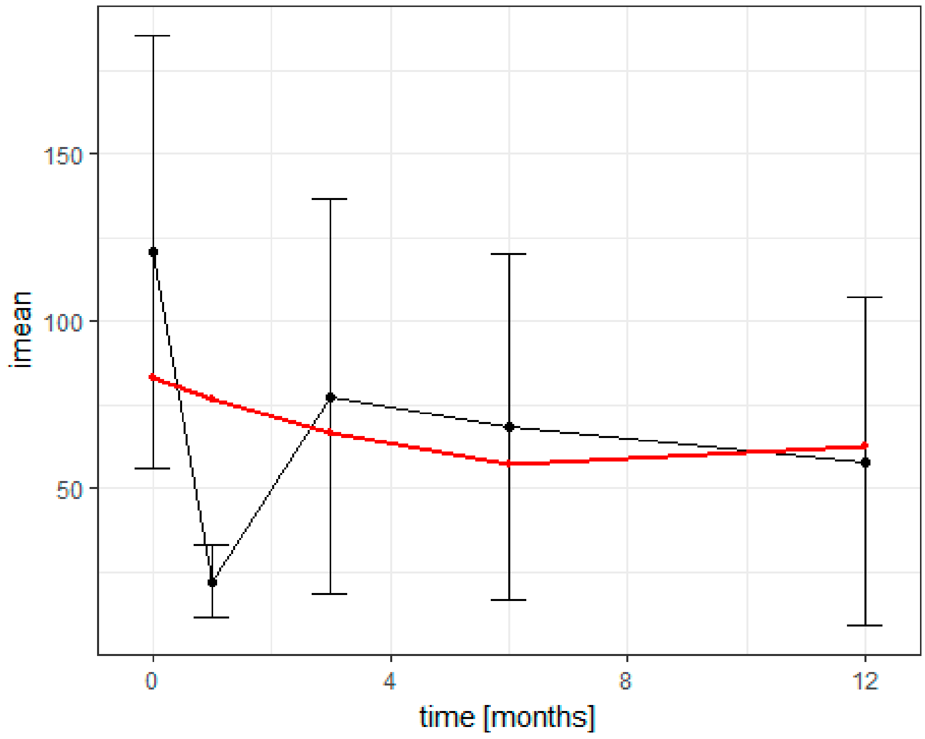

Table 60. Results of quadratic models for MEAN INSULIN CONCENTRATION (OGTT)

variable	without covariates	model 1	model 2	model 3	model 4	model 5	model 6	model 7	model 8	model 9	model 10	model 11	model 12	model 13	model 14	model 15
(Intercept)	83.3***	-18.4	82.6***	72.8***	21.3***	-28.7	-12.5	-42*	71.5***	18**	16.8**	-22.2	-44.7*	-40.4*	13.1	-42.1*
time_sq	0.430**	0.112	0.419**	0.431**	-0.495***	0.073	0.139	-0.687***	0.416**	-0.554***	-0.476**	0.101	-0.701***	-0.679***	-0.534***	-0.688***
time	-6.902***	-0.999	-7.0	-6.930***	4.861**	-0.625	-1.507	8.367***	-7.029***	5.396**	4.625*	-1.163	8.492***	8.214***	5.140**	8.254***

			68***													
total_mass_kg		0.768***				0.839***	0.704***	0.472***				0.769***	0.502***	0.453**		0.472**
IPAQ			0.000			0.000			0.000	0.000		0.000	0.000		0.000	0.000
sexM				23.143***			6.070		25.485***		12.415*	6.573		1.914	14.051*	2.987
diet_kcal					0.045***			0.047***		0.049***	0.045***		0.046***	0.047***	0.048***	0.046***
no. of observations	598	595	578	598	559	578	595	556	578	541	559	578	541	556	541	541
AIC	6545.9	6483.4	6344.9	6527.6	5965.6	6309.8	6476.9	5908.7	6325	5782.9	5956.3	6303.2	5770.2	5902.9	5772.6	5764.2

MEAN GLUCOSE CONCENTRATION (OGTT)

Figure 61. Distribution of MEAN GLUCOSE CONCENTRATION (OGTT) in time - linear fit

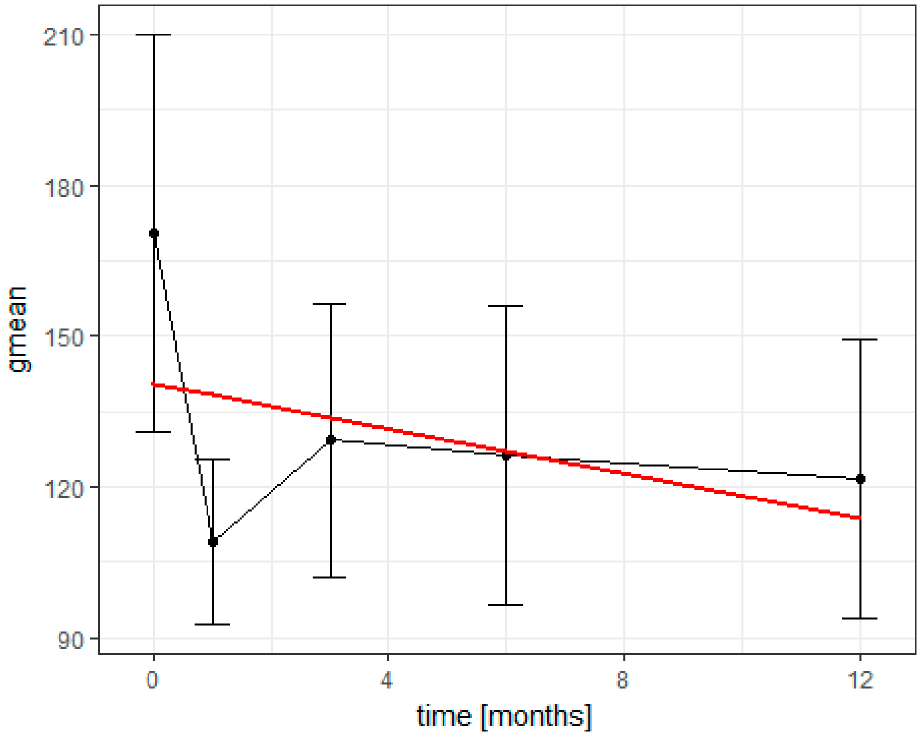

Table 61. Results of linear models for MEAN GLUCOSE CONCENTRATION (OGTT)

variable	without covariates	model 1	model 2	model 3	model 4	model 5	model 6	model 7	model 8	model 9	model 10	model 11	model 12	model 13	model 14	model 15
(Intercept)	140.5***	86.6***	140.7***	138.5***	111.6***	82.9***	80***	96.3***	138.4***	110.5***	112.4***	76***	96***	93.4***	111.2***	92.9***
time	-2.246***	-1.038**	-2.361***	-2.247***	-1.625***	-1.074**	-0.798	-1.349***	-2.361***	-1.721***	-1.624***	-0.838*	-1.391***	-1.222***	-1.720***	-1.263***
total_mass_kg		0.426***				0.453***	0.506***	0.115				0.535***	0.122	0.159		0.167
IPAQ			0.000			0.000			0.000	0.000		0.000	0.000		0.000	0.000
sexM				4.322			-7.964		5.078		-1.957	-8.121		-5.294	-1.630	-5.400

variable	without covariates	model 1	model 2	model 3	model 4	model 5	model 6	model 7	model 8	model 9	model 10	model 11	model 12	model 13	model 14	model 15
diet_kcal					0.027***			0.028***		0.029***	0.027***		0.028***	0.028***	0.029***	0.028***
no. of observations	602	599	581	602	563	581	599	560	581	544	563	581	544	560	544	544
AIC	5959.6	5905.8	5771	5953.8	5379.8	5745.2	5898.2	5338	5764.7	5206.8	5374.9	5737.5	5207.6	5331.8	5202	5201.4

Figure 62. Distribution of MEAN GLUCOSE CONCENTRATION (OGTT) in time - quadratic fit

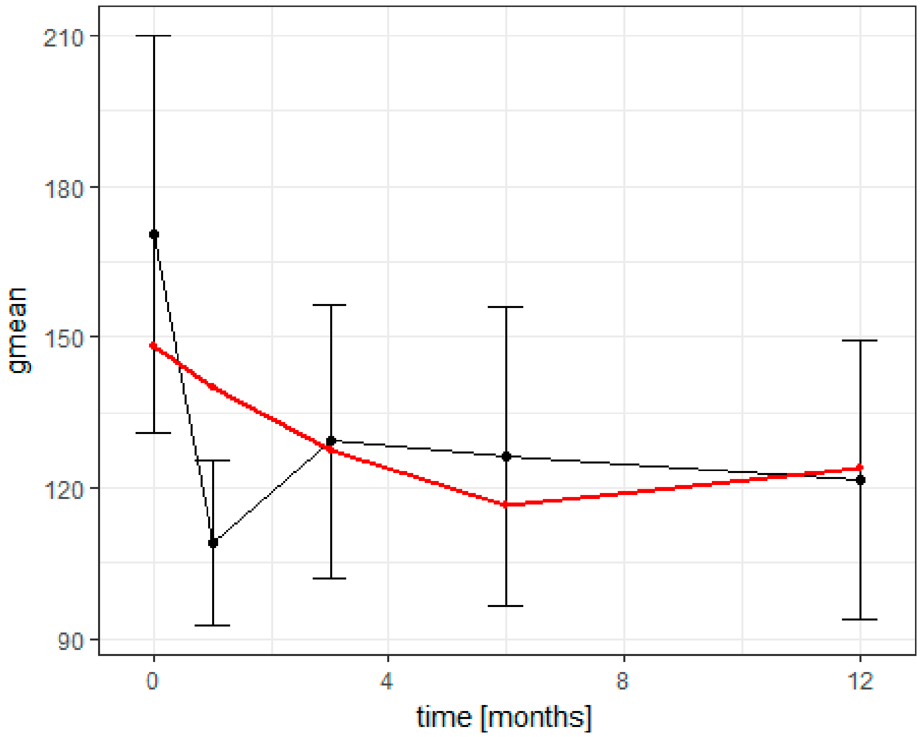

Table 62. Results of quadratic models for MEAN GLUCOSE CONCENTRATION (OGTT)

variable	without covariates	model 1	model 2	model 3	model 4	model 5	model 6	model 7	model 8	model 9	model 10	model 11	model 12	model 13	model 14	model 15
(Intercept)	148***	111.5***	148***	146***	112.5***	108.4***	108.4***	94.9***	145.8***	111.3***	113.2***	104.9***	95.6***	90.3***	111.8***	90.9***
time_sq	0.547***	0.431***	0.551***	0.548***	0.040	0.428***	0.416***	-0.024	0.550***	0.030	0.037	0.413***	-0.008	-0.047	0.028	-0.031
time	-8.568***	-6.437***	-8.740***	-8.572***	-2.093*	-6.461***	-6.170***	-1.049	-8.737***	-2.070*	-2.066*	-6.186***	-1.290	-0.613	-2.048*	-0.865

total_mass_kg		0.276***				0.298***	0.310***	0.122				0.335***	0.124	0.177		0.179
IPAQ			0.000			0.000			0.000	0.000		0.000	0.000		0.000	0.000
sexM				4.399			-3.064		5.009		-1.854	-3.231		-5.842	-1.553	-5.748
diet_kcal					0.027***			0.029***		0.029***	0.027***		0.028***	0.029***	0.029***	0.028***
no. of observations	602	599	581	602	563	581	599	560	581	544	563	581	544	560	544	544
AIC	5924.9	5887.4	5736.9	5919.1	5382.7	5727.6	5882.1	5341.1	5730.7	5209.8	5377.9	5722.2	5210.7	5334.6	5205	5204.3

MATSUDA INDEX

Figure 63. Distribution of MATSUDA INDEX in time - linear fit

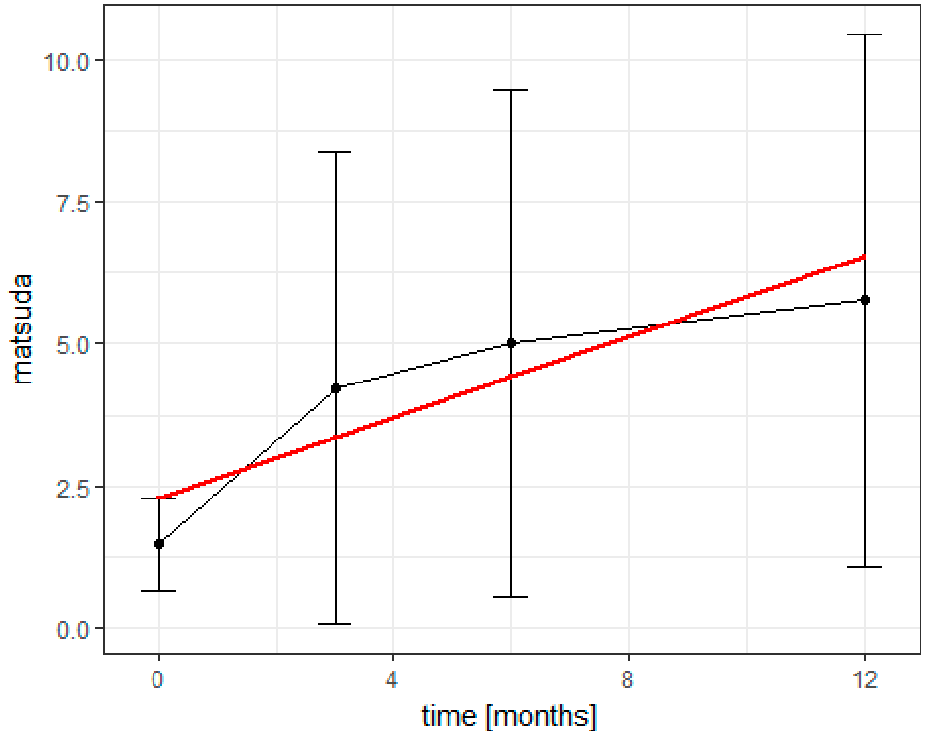

Table 63. Results of linear models for MATSUDA INDEX

variable	without covariates	model 1	model 2	model 3	model 4	model 5	model 6	model 7	model 8	model 9	model 10	model 11	model 12	model 13	model 14	model 15
(Intercept)	2.3***	9.7***	2.1***	3.3***	4.1***	9.7***	9.2***	9.7***	3.1***	4.1***	4.7***	9.1***	9.6***	9.1***	4.7***	9***
time	0.352***	0.175***	0.348***	0.353***	0.284***	0.168***	0.200***	0.163***	0.350***	0.277***	0.294***	0.193***	0.158***	0.190***	0.288***	0.185***
total_mass_kg		-0.058***				-0.059***	-0.051***	-0.048***				-0.051***	-0.049***	-0.040***		-0.040***
IPAQ			0.000			0.000			0.000	0.000		0.000	0.000		0.000	0.000
sexM				-2.124***			-0.889		-2.205***		-1.873***	-0.946		-1.006*	-1.906***	-1.020*
diet_kcal					-0.0			-0.0		-0.0	-0.0		-0.0	-0.0	-0.0	-0.0

					01***			01***		01***	01***		01**	01***	01***	01**
no. of observations	462	460	450	462	427	450	460	425	450	417	427	450	417	425	417	417
AIC	2458.9	2408.5	2421.8	2438	2291.3	2381.6	2404.9	2258.1	2399.9	2261.8	2275.6	2377.6	2242.1	2253.6	2245.8	2237.6

Figure 64. Distribution of MATSUDA INDEX in time - quadratic fit

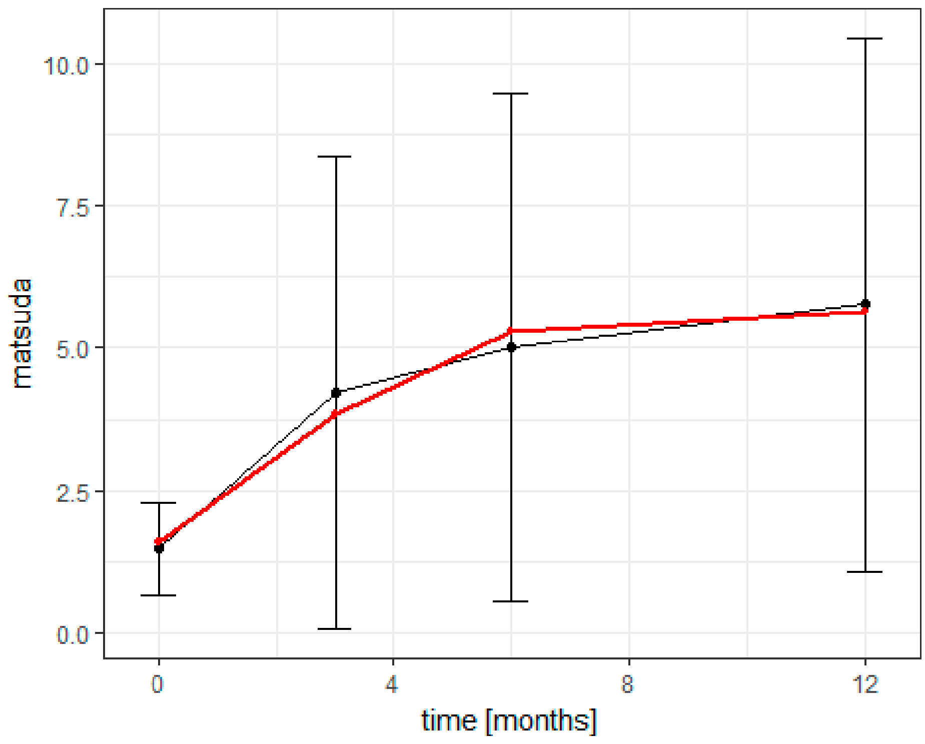

Table 64. Results of quadratic models for MATSUDA INDEX

variable	without covariates	model 1	model 2	model 3	model 4	model 5	model 6	model 7	model 8	model 9	model 10	model 11	model 12	model 13	model 14	model 15
(Intercept)	1.6***	8***	1.4***	2.6***	2.9***	8***	6.9***	9.1***	2.4***	3***	3.4***	6.9***	8.9***	7.9***	3.5***	7.7***
time_sq	-0.047***	-0.026**	-0.046***	-0.047***	-0.030**	-0.025**	-0.031***	-0.011	-0.046***	-0.026*	-0.035**	-0.030**	-0.011	-0.018	-0.032**	-0.019
time	0.895***	0.507***	0.885***	0.897***	0.662***	0.489***	0.611***	0.308	0.884***	0.614***	0.742***	0.595***	0.307	0.439*	0.696***	0.438*
total_mass_kg		-0.048***				-0.049***	-0.035***	-0.046***				-0.036***	-0.047***	-0.035***		-0.035**

IPAQ			0.000			0.000			0.000	0.000		0.000	0.000		0.000	0.000
sexM				-2.126***			-1.271*		-2.192***		-2.066***	-1.313*		-1.218*	-2.077***	-1.232*
diet_kcal					-0.001*			-0.001*		-0.001**	-0.001		-0.001*	-0.001*	-0.001*	-0.001
no. of observations	462	460	450	462	427	450	460	425	450	417	427	450	417	425	417	417
AIC	2436.3	2408.2	2401.2	2415.3	2291.8	2382.1	2401.4	2264.3	2379.5	2263.9	2273.3	2375	2248.3	2258.3	2245.6	2242.3

GLUCOSE AREA UNDER THE CURVE (OGTT)

Figure 65. Distribution of GLUCOSE AREA UNDER THE CURVE (OGTT) in time - linear fit

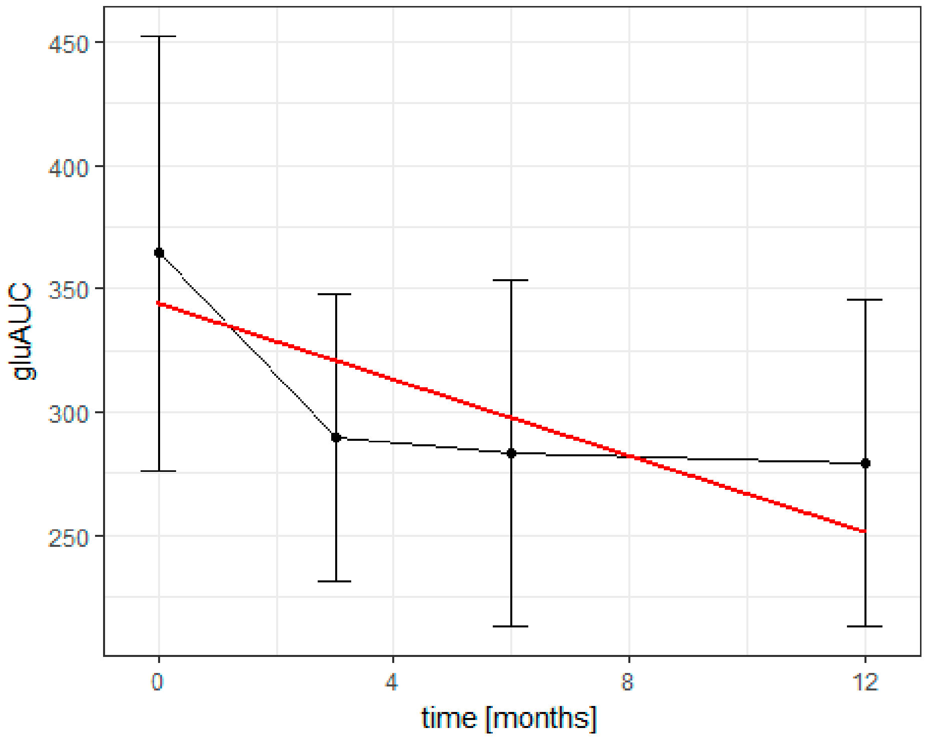

Table 65. Results of linear models for GLUCOSE AREA UNDER THE CURVE (OGTT)

variable	without covariates	model 1	model 2	model 3	model 4	model 5	model 6	model 7	model 8	model 9	model 10	model 11	model 12	model 13	model 14	model 15
(Intercept)	343.8***	216.4***	348.3***	346.5***	295***	224.4***	201***	225.3***	351.2***	294.6***	300***	207.7***	233.5***	213.1***	299.9***	220.2***
time	-7.727***	-4.659***	-7.614***	-7.693***	-5.480***	-4.622***	-3.709***	-3.961***	-7.577***	-5.326***	-5.377***	-3.660***	-3.969***	-3.223**	-5.219***	-3.201**
total_mass_kg		0.981***				0.945***	1.224***	0.561**				1.196***	0.521*	0.767***		0.739**
IPAQ			-0.000			-0.000			-0.000	-0.000		-0.000	-0.000		-0.000	-0.000
sexM				-5.809			-34.816**		-6.170		-12.546	-34.860*		-28.383*	-12.909	-29.021*

diet_kcal					0.034***			0.032***		0.037***	0.034***		0.032***	0.031***	0.037***	0.031***
no. of observations	382	380	375	382	351	375	380	349	375	345	351	375	345	349	345	345
AIC	4314.9	4274.5	4251.8	4308	3931.1	4235.7	4260.7	3900	4244.8	3874.7	3923.2	4221.9	3870.9	3888.3	3866.8	3858.9

Figure 66. Distribution of GLUCOSE AREA UNDER THE CURVE (OGTT) in time - quadratic fit

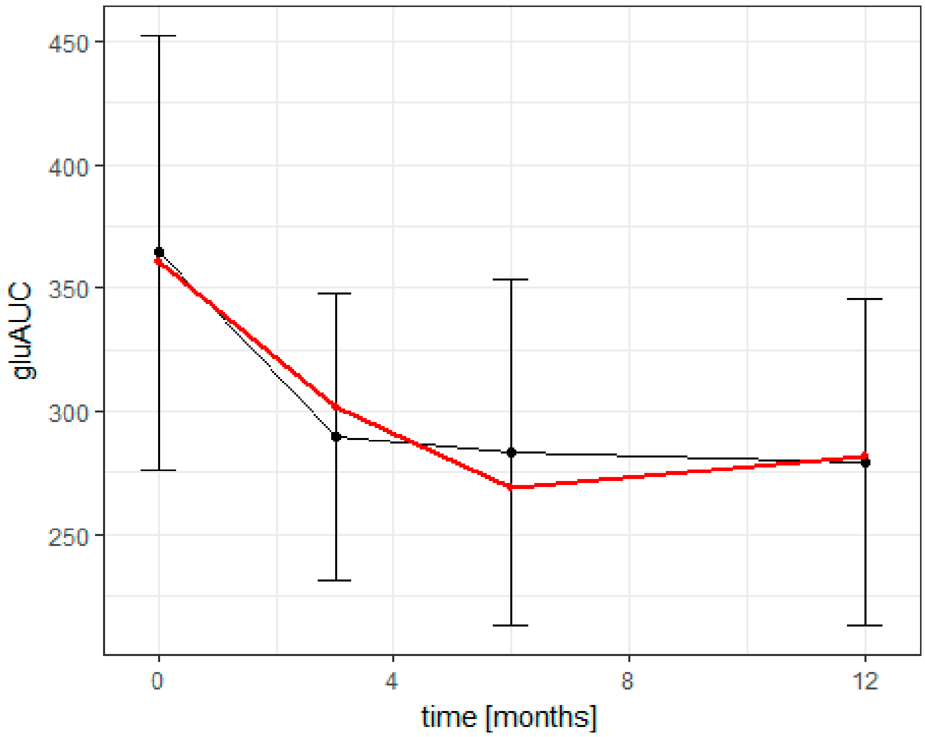

Table 66. Results of quadratic models for GLUCOSE AREA UNDER THE CURVE (OGTT)

variable	without covariates	model 1	model 2	model 3	model 4	model 5	model 6	model 7	model 8	model 9	model 10	model 11	model 12	model 13	model 14	model 15
(Intercept)	360.9***	328.6***	365.3***	362.2***	340.5***	338.5***	320.6***	306.5***	366.9***	343.1***	341.8***	330.2***	319.5***	297.2***	344.7***	309.6***
time_sq	1.452***	1.342***	1.457***	1.451***	1.246***	1.374***	1.297***	1.113***	1.456***	1.242***	1.236***	1.329***	1.185***	1.055***	1.230***	1.124***
time	-24.044***	-22.099***	-24.033***	-24.020***	-20.925***	-22.469***	-21.237***	-18.678***	-24.004***	-20.747***	-20.775***	-21.597***	-19.568***	-17.590***	-20.558***	-18.453***

total_mass_kg		0.240				0.197	0.336	0.229				0.295	0.184	0.333		0.292
IPAQ			-0.000			-0.000			-0.000	-0.000		-0.000	-0.000		-0.000	-0.000
sexM				-2.828			-10.805		-3.607		-4.143	-10.886		-11.985	-5.301	-12.275
diet_kcal					0.014*			0.016**		0.016*	0.014*		0.015*	0.016**	0.016*	0.015*
no. of observations	382	380	375	382	351	375	380	349	375	345	351	375	345	349	345	345
AIC	4247.5	4227	4186.7	4240.8	3898.8	4187.2	4219.3	3876.6	4180	3844.7	3892	4179.5	3845.2	3868.8	3837.7	3837.4

INSULIN AREA UNDER THE CURVE (OGTT)

Figure 67. Distribution of INSULIN AREA UNDER THE CURVE (OGTT) in time - linear fit

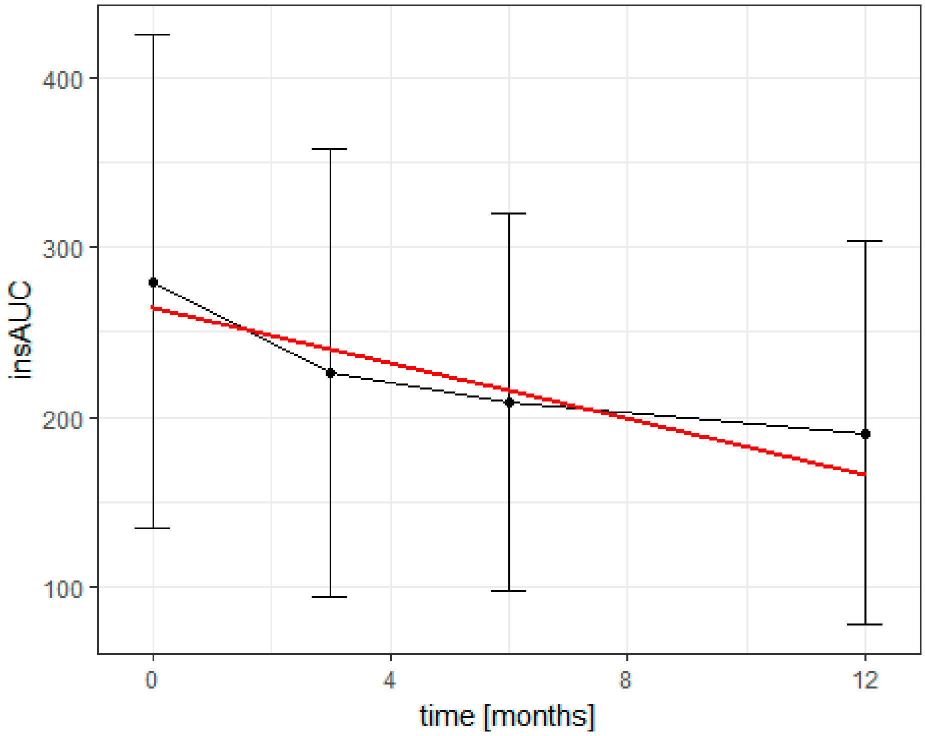

Table 67. Results of linear models for INSULIN AREA UNDER THE CURVE (OGTT)

variable	without covariates	model 1	model 2	model 3	model 4	model 5	model 6	model 7	model 8	model 9	model 10	model 11	model 12	model 13	model 14	model 15
(Intercept)	265***	75.9	275.2***	249.5***	220.5***	87*	75.3	86.9*	259.1***	224.8***	208.6***	86.7*	100.3*	89.8*	212.5***	103.3*
time	-8.217***	-3.637*	-7.939***	-8.373***	-6.217***	-3.376*	-3.614*	-3.367*	-8.105***	-5.640***	-6.439***	-3.380*	-2.945	-3.563*	-5.868***	-3.143
total_mass_kg		1.457***				1.438***	1.463***	1.111**				1.438***	1.080**	1.056**		1.025**
IPAQ			-0.001			-0.001			-0.001	-0.001		-0.001	-0.001		-0.001	-0.001
sexM				33.409			-0.557		34.626		29.466	0.456		8.008	30.008	7.953
diet_kcal					0.030***			0.024**		0.036***	0.029***		0.025**	0.025**	0.035***	0.025**

no. of observations	379	377	372	379	348	372	377	346	372	342	348	372	342	346	342	342
AIC	4632.2	4589	4562.1	4621.4	4246.2	4542.6	4581	4210.2	4551.1	4183.6	4236.2	4534.7	4174.6	4202.1	4173.4	4166.5

Figure 68. Distribution of INSULIN AREA UNDER THE CURVE (OGTT) in time - quadratic fit

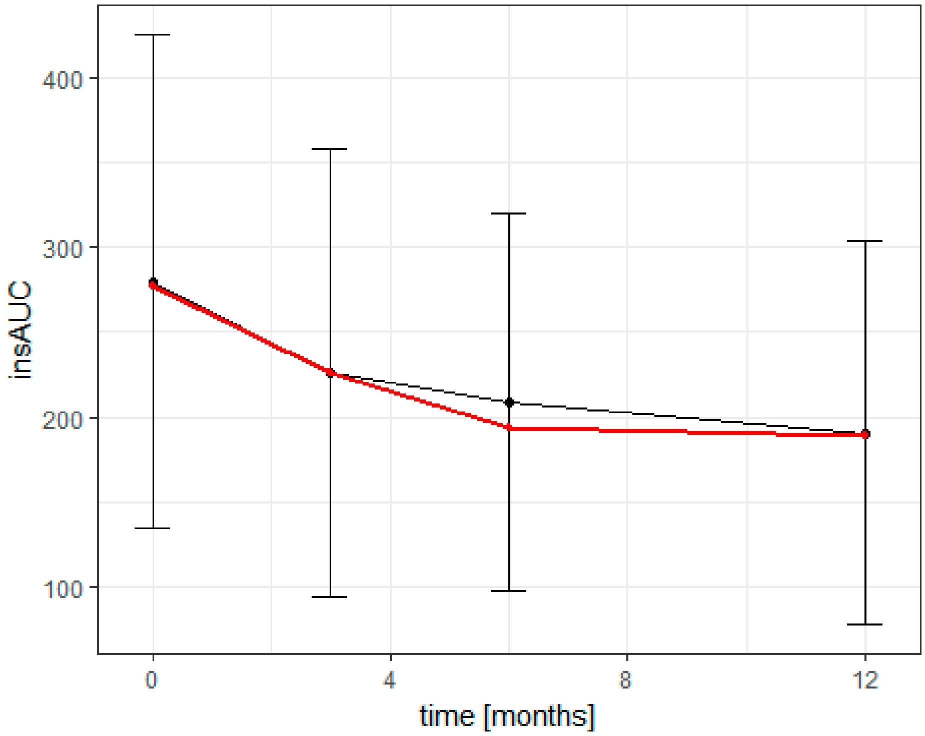

Table 68. Results of quadratic models for INSULIN AREA UNDER THE CURVE (OGTT)

variable	without covariates	model 1	model 2	model 3	model 4	model 5	model 6	model 7	model 8	model 9	model 10	model 11	model 12	model 13	model 14	model 15
(Intercept)	277.7***	125.2*	288***	261.3***	247.6***	138.4**	132.3*	107.4*	271.2***	248.9***	236.4***	146.6**	122*	116.9*	237.9***	131.6*
time_sq	1.086***	0.605	1.104***	1.099***	0.728*	0.636*	0.647*	0.292	1.115***	0.611	0.808*	0.683*	0.312	0.355	0.688	0.375
time	-20.444***	-11.471**	-20.405***	-20.737***	-15.296**	-11.612**	-12.284**	-7.214	-20.690***	-13.278**	-16.541***	-12.521**	-7.045	-8.375	-14.486**	-8.200
total_mass_kg		1.133**				1.102**	1.044**	1.031**				1.000*	0.998**	0.923*		0.889*

IPAQ			-0.001			-0.001			-0.001	-0.001*		-0.001	-0.001		-0.001*	-0.001
sexM				35.544			10.646		36.493		34.873	11.930		13.156	34.189	13.137
diet_kcal					0.019			0.020		0.025*	0.016		0.020	0.019	0.022*	0.020
no. of observations	379	377	372	379	348	372	377	346	372	342	348	372	342	346	342	342
AIC	4618.3	4585.8	4548.4	4607.1	4242.5	4539.2	4577.6	4209.7	4537	4181.2	4231.6	4530.9	4174	4201.3	4170.3	4165.6
